# Design and synthesis of hydrolyzable coumarin–naphthalimide dual probes for NIR fluorescence detection and selective photodynamic activity in cancer cells

**DOI:** 10.1039/d6ra08240h

**Published:** 2026-10-19

**Authors:** Benjamín Zúñiga-Salazar, Paula S. Rivero-Jerez, Sofía Pérez-Del Pino, Francisco Mura, Pablo Barrias, Martín Canals Díaz, José Robinson-Duggon, Alexis Aspée, Denis Fuentealba, Daniel Zúñiga-Núñez

**Affiliations:** a Laboratorio de Cinética y Fotoquímica, Facultad de Química y Biología, Universidad de Santiago de Chile Santiago Chile daniel.zunigan@usach.cl; b Laboratorio de Química Supramolecular y Fotobiología, Departamento de Química Física, Escuela de Química, Facultad de Química y de Farmacia, Pontificia Universidad Católica de Chile Santiago Chile dlfuente@uc.cl; c Universidad Mayor, Facultad de Ciencias, Ingeniería y Tecnología, Centro de Nanotecnología Aplicada Camino La Pirámide 5750 Huechuraba 8580745 Santiago Chile; d Universidad de Panamá, Facultad de Ciencias Naturales, Exactas y Tecnología, Departamento de Bioquímica Panamá 0824 República de Panamá; e Sistema Nacional de Investigación (SNI), Secretaría Nacional de Ciencia, Tecnología e Innovación (SENACYT) Panamá República de Panamá

## Abstract

Developing multifunctional agents capable of combining diagnostic and therapeutic functions remains a major challenge in cancer research. Herein, we report the design, synthesis, and photophysical characterization of a family of dual probes (C-NAF) based on coumarin–naphthalimide hybrids linked through an imine bond, combining near-infrared (NIR) fluorescence with light-induced cytotoxicity. C-NAF derivatives exhibit broad NIR emission and pronounced sensitivity to microenvironmental polarity, arising from locally excited (LE) and intramolecular charge-transfer (ICT) states modulated by solvent polarity. In their intact form, the C-NAF derivatives show negligible photodynamic activity. However, under polar protic conditions, imine hydrolysis generates photoactive naphthalimide aldehydes (NAF). Intracellular ROS detection and selective scavenger experiments indicate that the photodynamic response proceeds predominantly through a type I pathway involving radical-mediated ROS generation. Functionalization with butyl, morpholine, and biotin moieties was introduced to modulate physicochemical and molecular-recognition properties. *In vitro* studies in MCF-7 breast cancer cells showed that C-NAF derivatives are non-toxic in the dark and exhibit negligible phototoxicity under 540 nm irradiation, whereas significant phototoxicity occurs under 460 nm irradiation, consistent with photoactivity of the NAF hydrolysis products. C-NAF-B7 exhibited the strongest phototoxic response in MCF-7 cells, suggesting a possible contribution of the biotin moiety to cellular recognition or uptake. Conversely, negligible phototoxicity was observed in HEK-293 cells. Overall, these results identify C-NAF derivatives as promising dual probes combining NIR fluorescence with hydrolysis-activated, predominantly type I photodynamic activity and preferential photoactivity toward cancer cells under investigated conditions.

## Abstract 2

Developing multifunctional agents capable of combining diagnostic and therapeutic functions remains a major challenge in cancer research.

## Graphical abstract

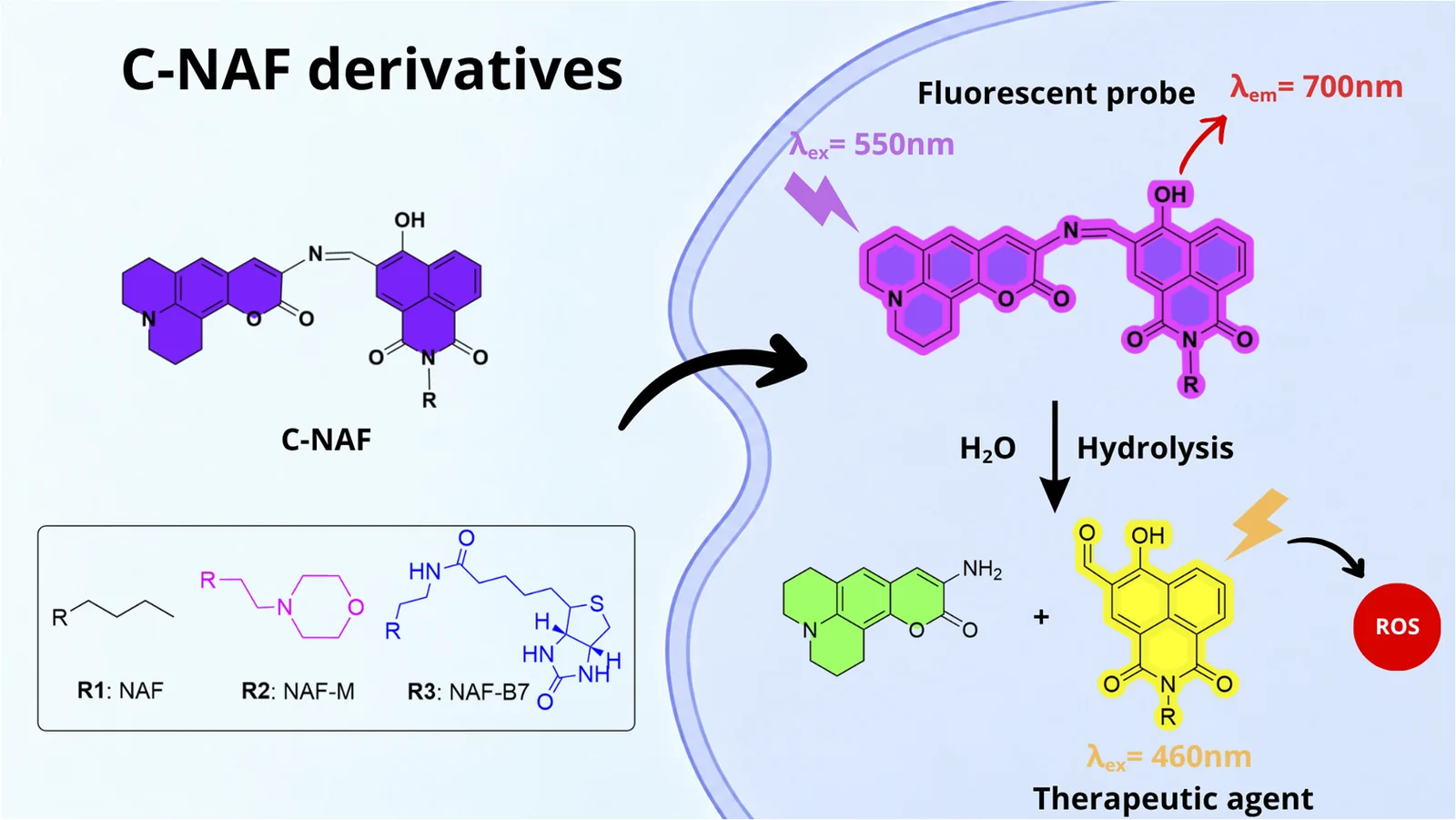

## 1 Introduction

Dual probes have emerged as a promising strategy in cancer detection and treatment, seamlessly integrating diagnostic and therapeutic functions.^1,2^ By enabling both biodistribution and real-time monitoring of drug administration, these agents provide valuable insights into treatment response and contribute to advancements in precision medicine. Notably, the combination of photodynamic therapy (PDT) and optical fluorescence detection, achieved through innovative strategies, has demonstrated a high ability to enhance therapeutic efficacy and improve cancer treatment outcomes.^3,4^

In these strategies, PDT offers a minimally invasive approach for treating cancerous tissues by employing a photosensitizer (PS), molecular oxygen, and light of an appropriate wavelength. Following administration and accumulation at the target site, the PS is activated by light exposure. In the presence of oxygen, this activation generates reactive oxygen species (ROS), which oxidize key biomacromolecules, such as amino acid residues in proteins, nitrogen bases in nucleic acids, and lipids in the cell membrane. The resulting extensive oxidative processes induce cell death through autophagy, necrosis, or apoptosis.^5,6^ On the other hand, the incorporation of fluorescent probes offers the possibility of employing bioimaging techniques for modern biomedical applications, enabling high-resolution tracking and diagnosis of cellular processes both *in vitro* and *in vivo*.^7–9^ In particular, near-infrared-emitting (NIR) fluorescent probes allow for deeper tissue penetration, minimizing biological photodamage of normal cells and reducing interference from autofluorescence enhancing spatial and temporal resolution, improving the accuracy of imaging detection in cancer tissues. The combination of PDT and fluorescence detection therefore provides a useful framework for development of dual-function photochemical probes. In addition, recent research has focused extensively on developing novel and more effective photosensitizers (PSs), along with the evaluation of their therapeutic potential *in vitro*, *in vivo* and preclinical studies aimed at assessing the of PDT.^10–12^ A critical challenge in optimizing PDT strategies is ensuring precise biodistribution and bioaccumulation of the PS within the targeted tumor or cancerous lesions.^13,14^

One strategy to increase selectivity involves conjugating PSs to molecular targets such as vitamins, antibodies, liposomes, macrocyclic compounds, and peptides, among others.^15,16^ Given the high metabolic activity of cancer cells, which drives rapid proliferation and increased demand for essential vitamins, the overexpression of vitamin-specific receptors on their surface provides an effective strategy. Sodium-dependent multivitamin transporter (SMVT) are significantly upregulated in a wide range of tumor, including breast, lung, ovarian, and colon cancers, enabling receptor-mediated uptake of biotin-conjugated molecules.^17^ Consequently, biotin functionalization has been explored as a strategy to enhance the interaction and uptake of photosensitizers in cancer cells. Several biotin-conjugated photosensitizers have shown improved cellular accumulation and photodynamic responses, although the underlying uptake mechanisms may depend on the molecular architecture and cellular model. Recent studies further demonstrate that biotin-functionalized PSs enhance tumor selectivity, and reduced toxicity in normal tissues, improving the precision and efficacy of PDT.^18,19^ Another strategy to increase the selectivity of photosensitizers (PSs) involves exploiting the ability of specific structural moieties to direct these compounds toward subcellular targets within cancer cells.^20,21^ For instance, it has been shown that the morpholine moiety is selectively accumulated in lysosomes, promoting the lysosomal membrane permeabilization (LMP), leading to the release of cathepsins into the cytosol and the activation of cell death pathways such as apoptosis or necrosis.^22,23^ Moreover, given the central role of lysosomes in regulating autophagy and cellular homeostasis, their disruption enhances therapeutic efficacy and amplifies pro-death signalling, making it a key strategy in the design of PS. In addition to receptor-targeting and subcellular localization strategies, structural modulation through the incorporation of alkyl chains has proven to be an effective approach to enhance the biological performance of photosensitizers. The introduction of alkyl moieties increases the hydrophobic character of PSs, promoting stronger interactions with cellular membranes and facilitating passive diffusion across lipid bilayers. This modification can significantly improve cellular uptake and intracellular retention, particularly when an optimal hydrophilic–lipophilic balance is achieved.^24,25^

In addition to employing cellular targeting strategies to improve cancer selectivity, the rational selection of an appropriate PS is essential for development and evaluation of PDT studies. In this context, naphthalimide-based compounds have emerged as promising PS in PDT due to their favourable photophysical and biological properties. This compound has demonstrated selective accumulation in cancer cells, attributed to its ability to localize within specific organelles. Furthermore, certain naphthalimide derivatives exhibit intrinsic anticancer activity independent of light-induced ROS generation, expanding their potential as effective therapeutic agents. Moreover, their structural versatility further allows functionalization with targeting moieties, improving selectivity and biocompatibility for PDT studies. Recent studies highlight their potential in both cancer therapy and imaging-guided PDT, as well as the development of type I and type II photosensitizers with enhanced antitumor efficacy.^26–28^

On the other hand, coumarin-based fluorescent probes have proven valuable tools for intracellular biological detection. Due to their favourable photophysical properties, such as high quantum yield, excellent photostability, and tuneable emission wavelengths, they are ideal candidates for biomedical applications. Coumarin-based fluorescent probes have been extensively investigated for sensing biologically relevant analytes and microenvironmental changes, including polarity, viscosity, pH, and reactive species. In this context, their ability to selectively target specific biomolecules or organelles further enhances the detection accuracy, allowing for more accurate tumor localization.^29,30^ However, coumarins dye primarily emit in the green region of the electromagnetic spectrum, which limits their application in deep biological imaging. To enhance their effectiveness, it is essential to shift their emission towards the red or near-infrared regions, where light penetration is greater and absorption by biomolecules is minimized, reducing interference from autofluorescence.

Herein, we designed and synthesized dual fluorescence probe-photosensitizers (1a–c, C-NAF) based on 7-aminocoumarin–naphthalimide hybrids, linked through an imine bond (Scheme 1). Although coumarin–naphthalimide conjugates have previously been reported,^31,32^ the molecular design of our molecules was conceived to promote a donor–acceptor architecture in which the 7-amino coumarin and naphthalimide units act as electron-donating and electron-accepting moieties, respectively, favouring intramolecular charge transfer (ICT) and shifting their fluorescence emission to the NIR region. On the other hand, the imine bond in the 1a–c derivatives was deliberately introduced due to its susceptibility to hydrolysis in polar protic media, leading to the formation of naphthalimide aldehyde derivatives (2a–c, NAF) with the concomitant ability for acting as a PS. In addition, butyl, morpholine, and biotin substituents were introduced to modulate the physicochemical and biological properties of the C-NAF platform, including hydrophobicity, potential subcellular interactions, and molecular-recognition behaviour. To evaluate the biological performance of the C-NAF derivatives, MCF-7 breast cancer cells were selected as a representative malignant cell model to assess the photodynamic behaviour of the different derivatives. In parallel, HEK-293 cells were included as a non-neoplastic comparison model to evaluate the cellular response to the C-NAF derivatives under the same experimental conditions and to determine whether their photodynamic effects differ.

**Scheme 1:**
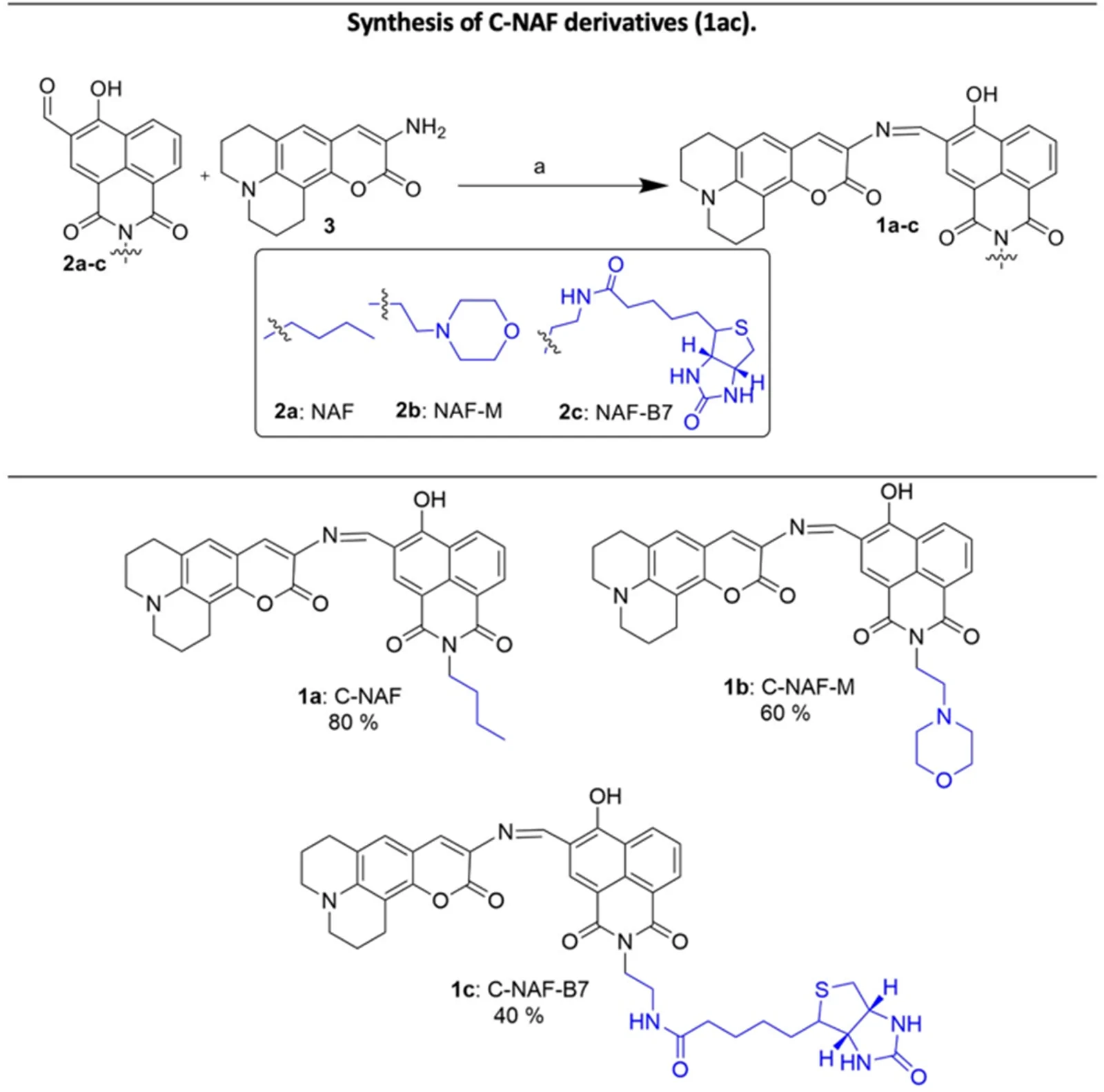
Molecular structures for coumarin–naphthalimide derivatives. 1a: *N*-butyl (C-NAF), 1b: *N*-morpholine (C-NAF-M), and 1c: *N*-biotin (C-NAF-B7). General reaction conditions: (a) 2a–c (0.30 mmol) and 3 (0.20 mmol), EtOH (3 mL), 80 °C, 6 h. Yields of 1a–c correspond to the isolated product.

## 2 Results and discussion

### 2.1 Synthesis of C-NAF derivatives (1a–c)

The C-NAF derivatives were obtained through a Schiff-base condensation reaction between the coumarin amine (3) with naphthalimide derivatives 2a–c, resulting in C-NAF, CNAF-M and C-NAF-B7, respectively (Scheme 1). The characterization of all final compounds was confirmed by ^1^H and ^13^C NMR studies, mass spectrometry, and analytical HPLC analysis, is available in the Sections S1 and S2 in the SI.

### 2.2 Photophysical properties of C-NAF derivatives in homogeneous media

The absorption and emission spectra of the C-NAF derivatives in different solvents are shown in Fig. 1. Notably, in all solvents, the maxima absorption is centred around 530 nm (Fig. 1A, C and E), while the fluorescence emission spectra exhibit a broad band extending from 550 nm to 800 nm with a pronounced red shift as solvent polarity increases. In contrast, to previously reported 7-aminocoumarins typically display absorption maxima in the range of 400–470 nm and emission spectra extended between 500 and 550 nm;^33–36^ the C-NAF derivatives show a significant bathochromic toward longer wavelength. This marked red shift and the solvent dependence are attributed to an enhanced intramolecular charge transfer (ICT) process from the coumarin moiety (electron donor) to the naphthalimide unit (electron acceptor). To further elucidate the origin of these optical properties, DFT and TD-DFT analysis was performed (Fig. 2). The frontier molecular orbital energy diagram shows relatively small HOMO–LUMO gaps (∼2.13–2.19 eV) for all derivatives, which is consistent with the experimentally observed red-shifted absorption and indicates that the incorporation of butyl, morpholine, and biotin fragments does not significantly alter the electronic properties of the chromophore core. In this regard, the molecular orbitals analysis of C-NAF-B7 reveals a clear donor–acceptor electronic structure, with the HOMO mainly localized on the coumarin unit and the LUMO distributed over the naphthalimide fragment, supporting the ICT character of the lowest-energy transition; a similar result was obtained for C-NAF and C-NAF-M (see section S3, Fig. S40 and S41 in SI). In agreement with this, the calculated absorption spectra showed an absorption maxima band in the 560–580 nm range, close to the experimental values. Moreover, calculations performed in different solvents reveal only minor variations depending on solvent polarity (Fig. 2B), reproducing the weak solvatochromic behaviour observed experimentally in absorption (Fig. 1).

**Fig. 1:**
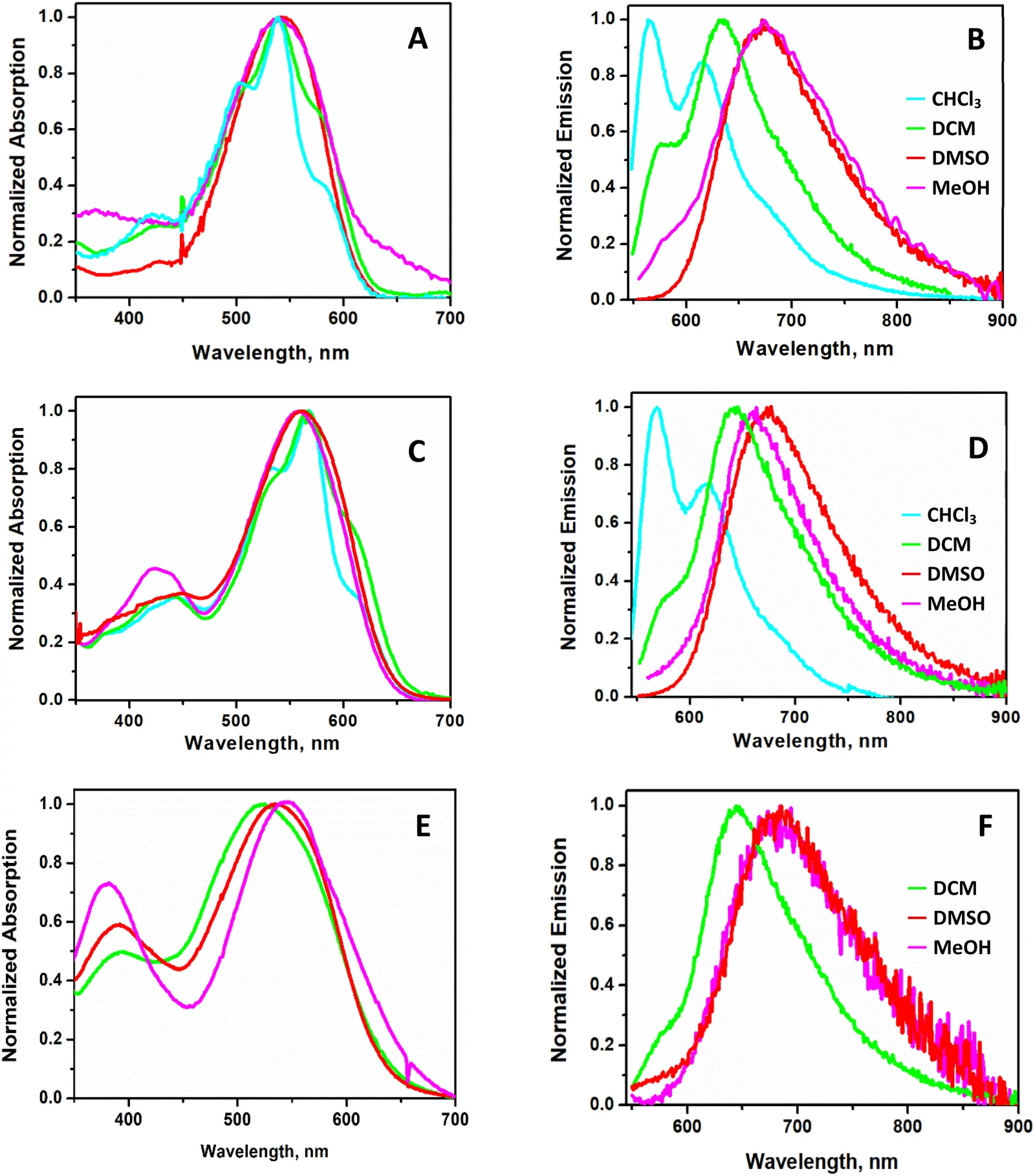
Normalized UV-vis absorption (A, C and E) and fluorescence emission spectra (B, D and F) of C-NAF (A and B), C-NAF-M (C and D), and C-NAF-B7 (E and F) in methanol (MeOH), dimethyl sulfoxide (DMSO), dichloromethane (DCM), and chloroform (CHCl_3_) at room temperature under air. The corresponding solvents are identified by the legend included in the fluorescence emission panel.

**Fig. 2:**
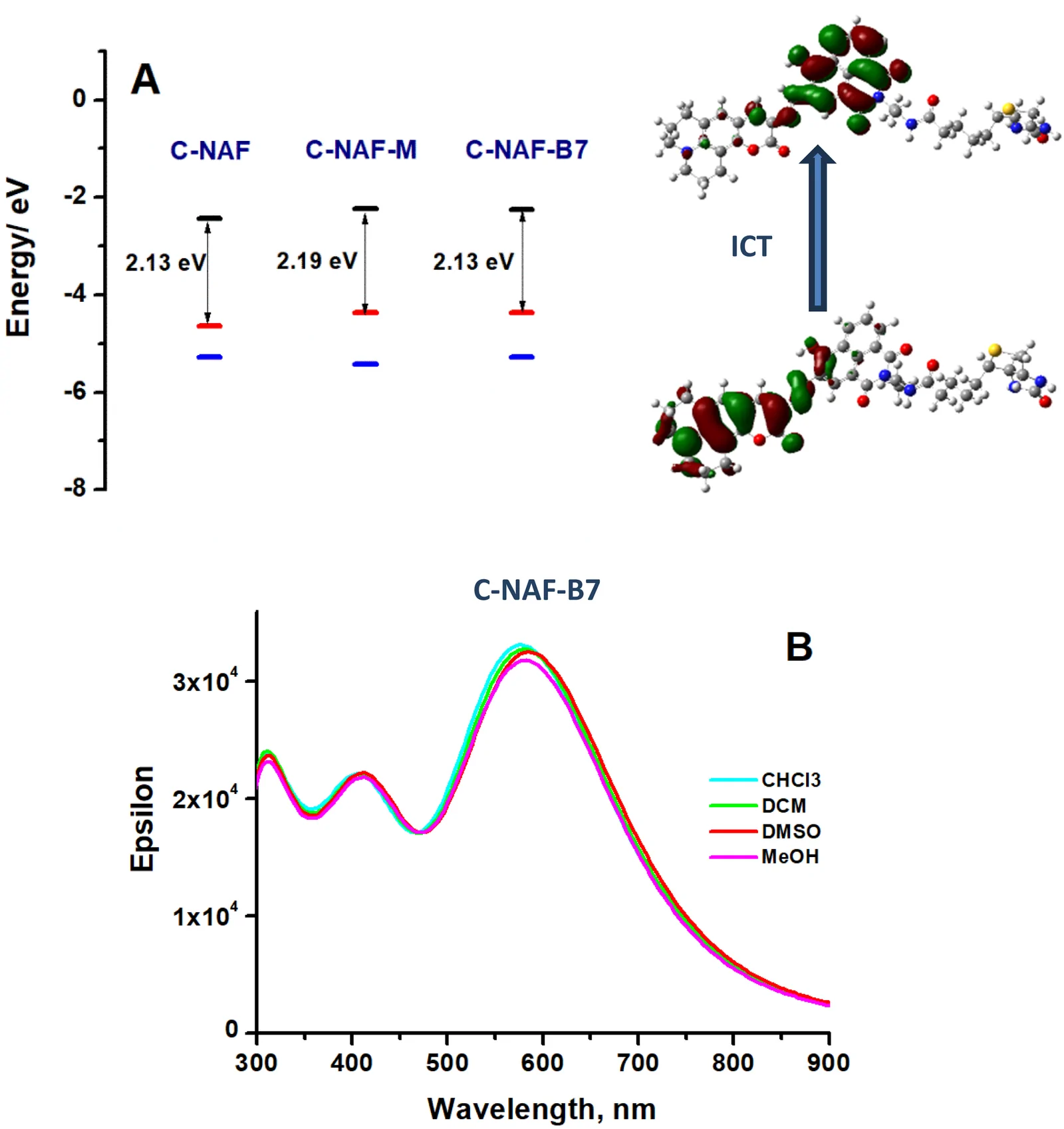
(A) Frontier molecular orbital energy diagram (HOMO–LUMO band gap) for C-NAF, C-NAF-M, and C-NAF-B7, together with the HOMO and LUMO isosurface representations of C-NAF-B7 (isovalue = 0.020 a.u.). (B) Theoretical absorption spectra of C-NAF-B7 in different solvents (CHCl_3_, DCM, DMSO, and MeOH) calculated using TD-DFT at the B97D/6-31G** level with the C-PCM solvation model (TD (NStates = 50)).

Overall, the theoretical results are consistent with the experimentally observed solvatochromic behaviour and the progressive red shift in fluorescence emission with increasing solvent polarity. As a result, the fluorescence emission spectral response is strongly shifted toward longer wavelengths, reaching the near-infrared (NIR) region. This is particularly advantageous for potential bioimaging applications, as this NIR fluorescence emission considerably reduces light scattering, and minimizes interference from biological autofluorescence, thereby improving imaging sensitivity and signal-to-noise ratio. These photophysical characteristics support the potential of the C-NAF derivatives as environment-sensitive fluorescent probes operating in the red-to-NIR spectral region.^37–39^

On the other hand, it is observed that the absorption and fluorescence spectra in chloroform and dichloromethane present a pattern of resolved spectral bands, suggesting that the emission originates predominantly from a locally excited (LE) state. In contrast, in more polar solvents such as dimethyl sulfoxide and methanol, the emission spectra evolve into broader, structureless bands, which can be attributed to the formation of an intramolecular charge transfer (ICT) excited state stabilized by increased solvent polarity (Fig. 1B, D and F).^33,40^ However, these structural modifications do influence the solubility of the compounds; for instance, C-NAF-B7 is insoluble in chloroform (*vide infra*).

Fluorescence lifetime measurements were performed to evaluate the relative contributions of LE, and ICT excited states in different homogeneous media. As summarized in Table 1, three fluorescence lifetimes are observed for C-NAF derivatives in polar solvents (methanol and DMSO). The shortest component (*τ*_1_ = 0.04–0.20 ns) corresponds to a fast deactivation process, which is attributed to non-radiative decay associated with free rotation around the imine bond. This behaviour is consistent with a tautomeric equilibrium between enol–imine and keto–enamine forms, facilitating efficient non-radiative relaxation pathways, as previously reported.^41,42^ Interestingly, this short lifetime observed for C-NAF derivatives is observed in solvents with large values of *β* (hydrogen bond acceptor) MeOH (*α* = 0.98, *β* = 0.62) and DMSO (*α* = 0, *β* = 0.76) in agreement with a stabilization of the enol–imine form of the imine by solvent intermolecular hydrogen bond, and in consequent contribute to non-radiative deactivation, in comparison with chloroform (*α* = 0.2, *β* = 0.1) and dichloromethane (*α* = 0.13, *β* = 0.1) that have small *β* values.^43^ For C-NAF-B7, the shortest lifetime component was not resolved in methanol. This behaviour can be associated with its comparatively low solubility and propensity to form molecular aggregate, inhibiting free rotation on the enol–imine form of even its formation. The second lifetime (*τ*_2_ = 0.20–1.14 ns) is attributed to a LE excited state, showing an increase in the contribution from methanol to chloroform for all C-NAF derivatives (*A*_2_ = 0.80–0.95). Finally, the longest lifetime (*τ*_3_ = 1.51–8.75 ns) is attributed to the ICT excited state from the 7-aminocoumarin (donor) fragment to the naphthalimide (acceptor). This component exhibits longer lifetimes in polar solvents such as methanol and DMSO, reflecting the stabilization of the ICT state by solvent polarity. As the solvent polarity decreases, *τ*_3_ is reduced, in agreement with previous reports for 7-aminocoumarin derivatives, and its relative contribution to the overall fluorescence emission also diminishes (*A*_3_ = 0.01–0.30).^33,44^

**Table 1:** Fluorescence lifetimes, pre-exponential factors and quantum yield of fluorescence of C-NAF, C-NAF-M and C-NAF-B7 in different solvents. Measurements were performed using a 507 nm laser diode as the excitation source. Emission decays were recorded at the maximum emission wavelength for each solvent, under air at room temperature. Rose of Bengal was used as standard *φ*_std_ = 0.08

Compound/solvents	*τ*_1_, ns	*A*_1_	*τ*_2_, ns	*A*_2_	*τ*_3_, ns	*A*_3_	*χ* ^2^	*Φ*_F_
**C-NAF**								
Chloroform	—	—	0.56	0.90	1.90	0.10	1.09	0.23
Dichloromethane	—	—	0.40	0.90	1.51	0.10	1.13	0.14
DMSO	0.26	0.10	0.47	0.83	4.41	0.03	1.07	0.03
Methanol	0.04	0.09	0.20	0.89	3.74	0.02	1.20	ND

**C-NAF-M**								
Chloroform	—	—	0.51	0.90	1.90	0.10	1.18	0.20
Dichloromethane	—	—	0.58	0.69	2.38	0.31	1.13	0.11
DMSO	0.38	0.10	1.14	0.87	6.63	0.03	1.13	0.05
Methanol	0.15	0.14	0.47	0.83	4.12	0.03	1.07	ND

**C-NAF-B7**								
Chloroform	—	—	—	—	—	—	—	
Dichloromethane	—	—	0.75	0.62	1.59	0.38	1.20	0.12
DMSO	0.30	0.61	0.90	0.34	8.75	0.05	1.20	0.08
Methanol	—	—	0.46	0.9l9	2.21	0.01	1.21	ND

Regarding the fluorescence quantum yield, Table 1 showed an increase from 0.03 to 0.23 as solvent polarity decreases for all C-NAF derivatives from DMSO, dichloromethane and chloroform. This behaviour can be attributed to a reduction in non-radiative deactivation pathways, particularly those associated with rotation and isomerization around the imine bond.^45–47^ Finally, singlet oxygen generation by the C-NAF derivatives was evaluated using an indirect method with 9,10-anthracenediyl-bis(methylene) dimalonic acid (ABMA) as a singlet oxygen scavenger and rose bengal as the reference photosensitizer (*Φ*_Δ_ = 0.70). The determined singlet oxygen quantum yields reveal that the C-NAF derivatives are poor generators of singlet oxygen (*Φ*_Δ_ < 0.001).

Due to the important photophysical properties of C-NAF derivatives in homogenous media, these compounds are promising candidates as near-infrared fluorescent probes (NIRF) for monitoring changes in the polarity of cell membranes. To assess their potential for fluorescence imaging applications under biologically relevant conditions, their photophysical behaviour was further evaluated in relevant biological media, as described in Section 2.4.

### 2.3 Hydrolysis of C-NAF derivatives in methanol as solvent

As mentioned at the outset, the design of these probes was based on an incorporation of an imine bond susceptible to hydrolysis, with the aim of enhancing the photosensitizing properties of naphthalimide core (see below). Accordingly, hydrolysis experiments of the C-NAF derivatives were conducted in methanol containing 0.02% water. Specifically, Fig. 3A–C illustrate the hydrolysis of the C-NAF derivatives over 90 min, showing a progressive decrease in the absorption maxima at 530–540 nm for all compounds, accompanied by a simultaneous increase in absorbance in the 390–400 nm region over time.

**Fig. 3:**
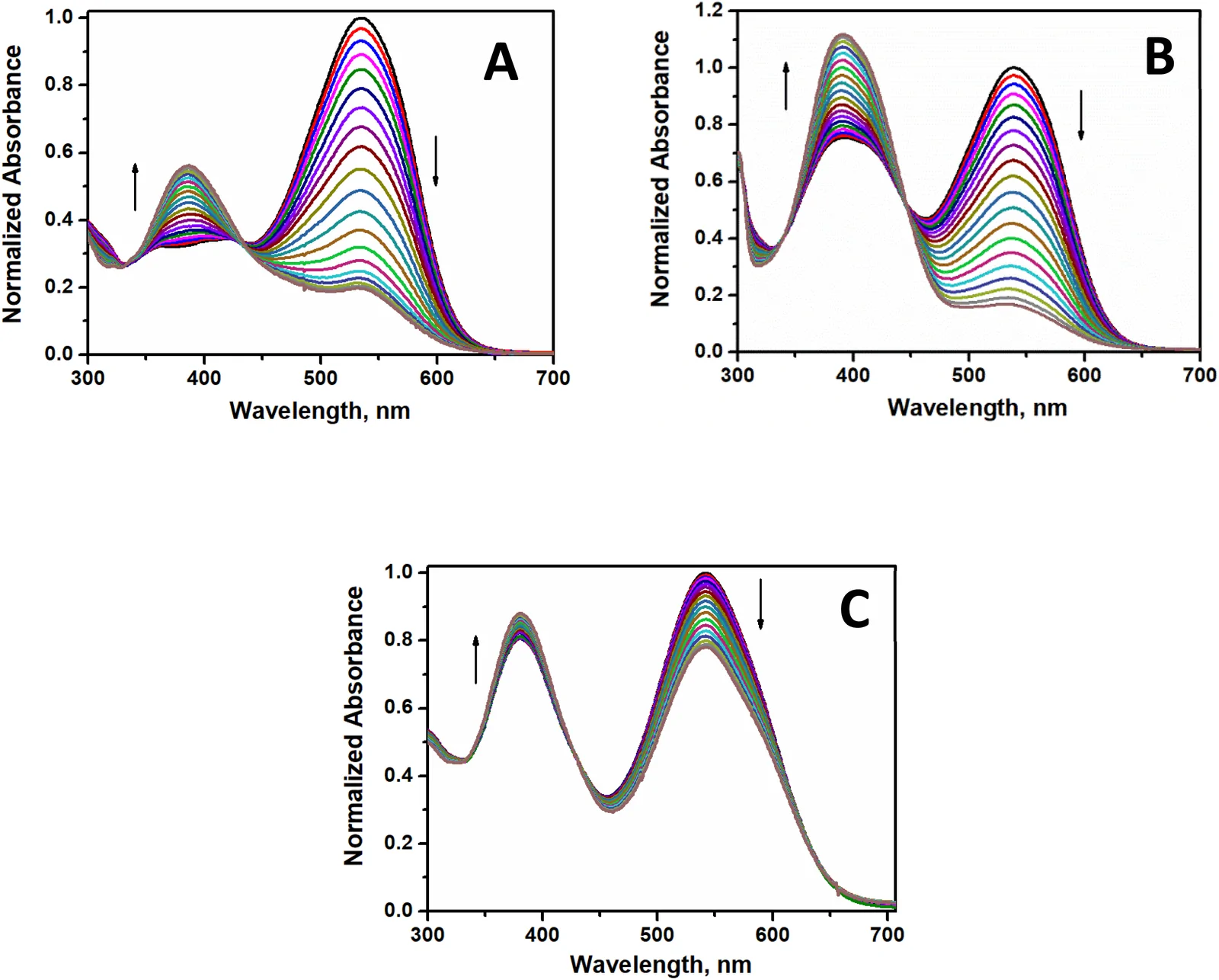
Normalized UV-visible spectral changes recorded during hydrolysis of 12 µM C-NAF (A), C-NAF-M (B) and C-NAF-B7 (C) derivatives in methanol containing 0.02% water at room temperature under air, recorded until 90 min.

This spectral behaviour is attributed to hydrolysis of the imine bond, leading to molecular fragmentation and the formation of the corresponding aldehyde and amine precursors—namely, the coumarin amine (3, C-NH_2_) and the naphthalimide aldehydes derivative (2a–c, NAF). The increase in absorbance near 400 nm is consistent with the absorption spectra of these precursors in methanol (see Fig. S42 in SI). The hydrolysis process of C-NAF derivatives was confirmed by UHPLC MS/MS spectrometry studies, confirming that the hydrolysis products correspond to the precursors in the synthesis of each derivative (see Fig. S43 and S45 in SI).

To quantitatively characterize the hydrolysis kinetics, the time-dependent absorbance changes at the absorption maximum of each C-NAF derivative were fitted directly to a pseudo-first-order monoexponential decay model, *A*_*t*_ = *A*_∞_ + (*A*_0_ − *A*_∞_)e^−*k*_obs_*t*^, where *A*_0_, *A*_*t*_, and *A*_∞_ represent the initial absorbance, the absorbance at time *t*, and the asymptotic absorbance after hydrolysis, respectively. The resulting *k*_obs_ values were 0.078 min^−1^, 0.054 min^−1^, and 0.047 min^−1^ for C-NAF, C-NAF-M, and C-NAF-B7, respectively, corresponding to hydrolysis half-lives (*t*_1/2_ = ln 2/*k*_obs_) of 8.89 min, 12.74 min, and 14.52 min, respectively (Fig. S46 and Table S1). Thus, the hydrolysis rate follows the order C-NAF > C-NAF-M > C-NAF-B7. These results demonstrate that all three derivatives undergo hydrolysis on a relatively short timescale under the experimental conditions, although the hydrolysis rate is modulated by the substituent attached to the naphthalimide moiety.

These kinetic results further support the hydrolysable nature of the C-NAF platform and provide quantitative evidence that the three derivatives undergo substantial transformation on the timescale of the experiments. According to the literature, both hydrolysis products can produce excited triplet capable of promoting ROS generation.^48,49^ In this context, singlet oxygen generation by the naphthalimide aldehyde derivatives (2a–c, NAF) and the coumarin amine (3, C-NH_2_) was evaluated using an indirect method with ABMA as a singlet oxygen scavenger and riboflavin as a standard (*Φ*_Δ_ = 0.61) in ultrapure water. The results show a clear ABMA consumption upon irradiation of the NAF derivatives, whereas only minimal ABMA degradation was observed under irradiation of C-NH_2_ (Fig. S47 in SI). The singlet-oxygen quantum yields summarized in Table 2 confirm that the NAF aldehyde derivatives efficiently produce singlet oxygen, while C-NH_2_ exhibits low generation yields—values that are nevertheless comparable to those previously reported to induce cell death in phototoxicity studies. As mentioned above, C-NAF derivatives do not generate singlet oxygen upon irradiation at 540 nm and therefore cannot be used as photosensitizers (*Φ*_Δ_ < 0.001), which is where they are irradiated to detect fluorescence emissions. However, after hydrolysis singlet oxygen generation is enhanced at least 20-times for NAF, 78-times for NAF-M and 71-times for NAF-B7. These results support hydrolysis of the imine linkage as an activation mechanism that converts the weakly photosensitizing intact C-NAF probes into more photoactive NAF species.

**Table 2:** Singlet oxygen quantum yields of NAF aldehyde derivatives and C-NH_2_ in ultrapure water under air at room temperature, upon LED irradiation at 450 nm

Compound	*Φ*_Δ_
C-NH_2_	<0.005
NAF	0.020
NAF-M	0.078
NAF-B7	0.071

### 2.4 Photostability and physicochemical behaviour of C-NAF derivatives in biological media

The practical application of fluorescent theranostic probes requires not only suitable photophysical properties but also sufficient stability under the experimental conditions employed for biological studies. Therefore, the photostability, behaviour in biological medium, concentration-dependent spectral response, and pH stability of the C-NAF derivatives were evaluated under conditions representative of the *in vitro* imaging and photodynamic therapy experiments. These studies were performed to verify that the probes remain spectroscopically stable and adequately dispersed throughout the experimental time window used for the biological assays. Since the C-NAF derivatives are not directly soluble in aqueous media, stock solutions were prepared by dissolving 2 mg of the C-NAF derivative in 1 mL of DMSO. These stock solutions were subsequently diluted into Dulbecco's Modified Eagle Medium (DMEM), maintaining the final DMSO concentration below 1% (v/v), following the same protocol employed in all biological experiments.

To assess the behaviour of the C-NAF derivatives under biologically relevant conditions, UV-vis absorption spectra were recorded at increasing probe concentrations in physiological medium. As shown in Fig. 4, all derivatives exhibited a progressive increase in absorbance with concentration while preserving the spectral profile and the position of the absorption maximum, no spectral broadening, band shifts, or baseline distortions were observed, indicating the absence of detectable aggregation or precipitation within the investigated concentration range. Furthermore, the Beer–Lambert plots displayed excellent linearity (*R*^2^ > 0.99), confirming that the probes remained molecularly dispersed. Notably, the comparable *ε* values obtained for the three derivatives indicate that substitution with butyl, morpholine, or biotin does not substantially alter the absorption properties of the coumarin–naphthalimide chromophore core. Importantly, the concentration used for all biological experiments (1.0 µM) lies well within the linear range, demonstrating that the C-NAF derivatives possess adequate aqueous compatibility for the *in vitro* imaging and photodynamic therapy studies described herein.

**Fig. 4:**
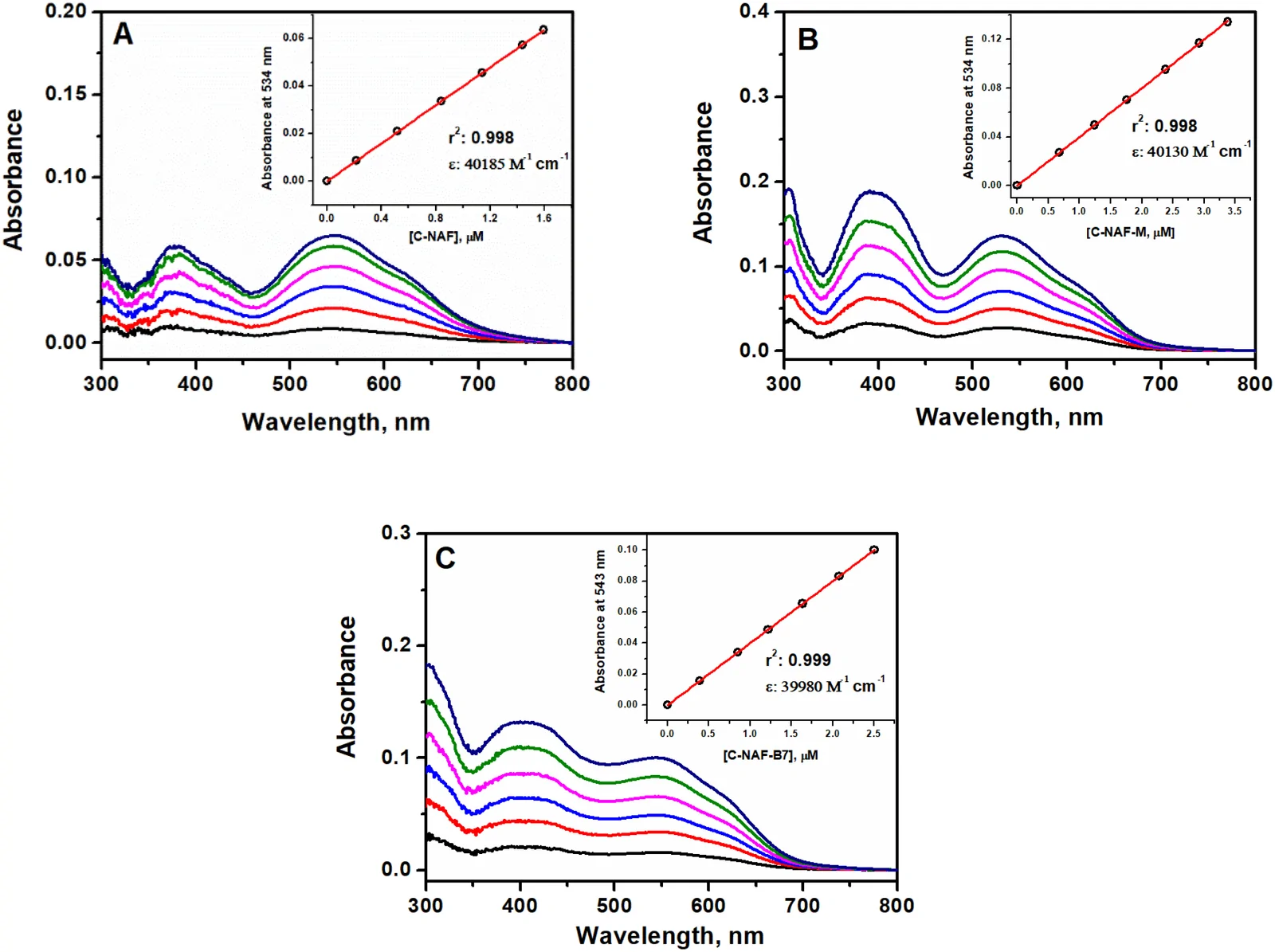
Evaluation of the aqueous compatibility of the C-NAF derivatives in DMEM. UV-vis absorption spectra of (A) C-NAF, (B) C-NAF-M, and (C) C-NAF-B7 recorded at increasing concentrations in Dulbecco's Modified Eagle Medium (DMEM) containing less than 1% (v/v) DMSO. Insets show the corresponding Beer–Lambert calibration plots obtained by monitoring the absorbance at 534 nm. The *R*^2^ values, and molar absorption coefficients (*ε*) are indicated in each inset.

On the other hand, to evaluate the suitability of the C-NAF derivatives for fluorescence imaging, their photostability was investigated by monitoring the normalized absorbance (*A*/*A*_0_) under continuous irradiation with a 540 nm LED and under dark conditions in both DMSO and DMEM (Fig. 5).

**Fig. 5:**
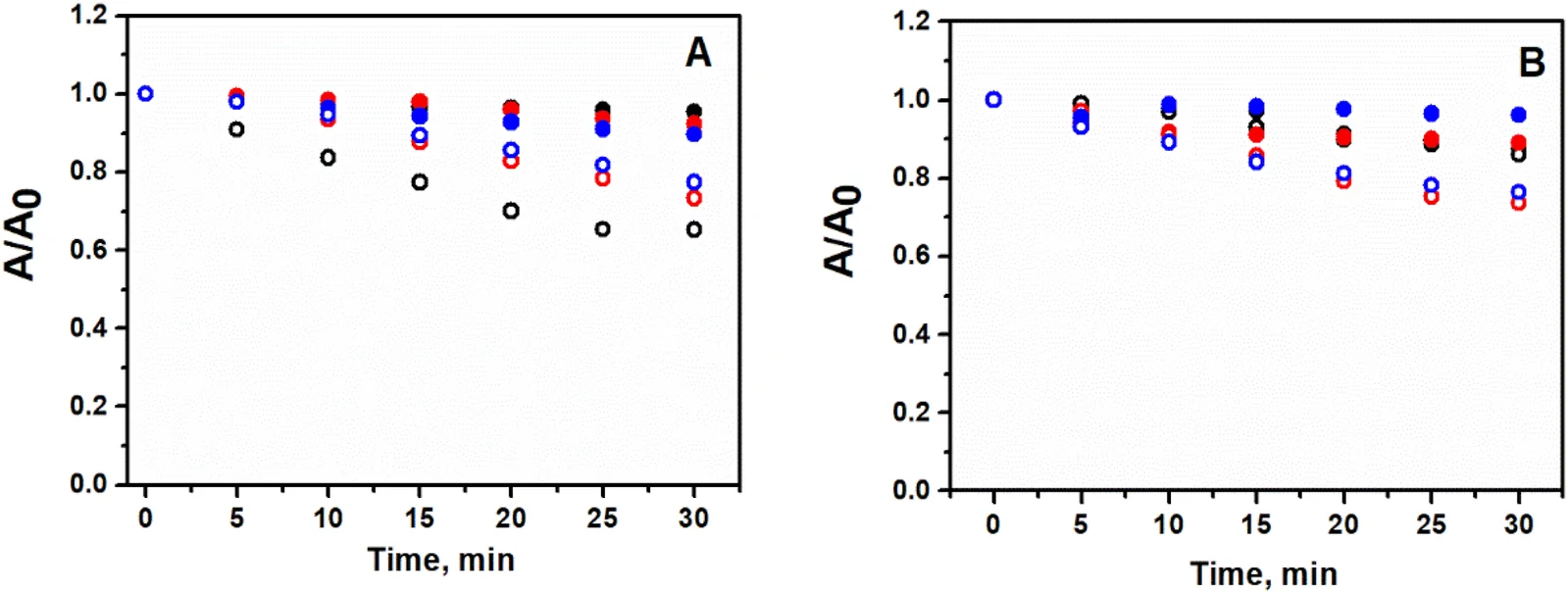
Photostability evaluation of the C-NAF derivatives. Normalized absorbance (*A*/*A*_0_) of C-NAF (black), C-NAF-M (red), and C-NAF-B7 (blue) measured under dark conditions (filled circles) and upon continuous irradiation with a 540 nm LED (open circles) for 30 min in (A) DMSO and (B) DMEM.

In the absence of irradiation, the three derivatives (C-NAF, C-NAF-M, and C-NAF-B7) exhibited excellent stability during the 30 minute experimental period, with minimal changes in absorbance in both solvents (Fig. 5A and B, filled circles). In contrast, continuous exposure to light induced a gradual decrease in absorbance intensity (empty circles), indicating that the compounds undergo photoinduced degradation. Interestingly, the magnitude of the absorbance decrease depended on the solvent. The C-NAF derivatives showed greater photostability in DMSO, while a more pronounced decrease was observed in DMEM, suggesting that the presence of water and other components of the biological medium promotes a photoinduced hydrolysis process. This behaviour is consistent with the known susceptibility of the imine linkage of the coumarin–naphthalimide backbone to irradiation and indicates that absorbance decrease occurs predominantly through a spontaneous hydrolysis process. It is important to note that, despite this gradual decrease, the probes remained between 70% and 90% of their initial absorbance after 30 minutes of irradiation, depending on the derivative and the medium. This demonstrates sufficient stability for fluorescence imaging applications.

This behaviour was corroborated by evaluating the fluorescence emission spectra of the C-NAF derivatives under 540 nm irradiation. Fig. S48 in SI shows that the fluorescence emission of the C-NAF derivatives decreases slightly during continuous irradiation in DMEM. The reduction in fluorescence intensity is most pronounced for C-NAF-M, while C-NAF and C-NAF-B7 retain most of their initial emission after 30 minutes of irradiation. These results are consistent with photoinduced hydrolysis inferred from the absorption measurements (Fig. 5) and demonstrate that the probes maintain sufficient fluorescence stability during the irradiation period used in the biological assays.

Finally, the influence of pH on the spectral stability of the probes was evaluated by recording their absorption spectra at pH 5.0, 7.0, and 8.0. No significant changes in either the absorption maxima or the spectral profiles were observed over this pH range (Fig. S49, in SI), demonstrating that the electronic structure of the C-NAF derivatives remains essentially unaffected under acidic, neutral, and slightly basic conditions relevant to biological environments.

### 2.5 Phototoxicity against MCF-7 and HEK-293 cells *in vitr*o

To evaluate the potential of C-NAF derivatives as photosensitizers, *in vitro* assays were performed to assess both cytotoxicity and phototoxicity in MCF-7 breast cancer and HEK-293 non-neoplastic cells. Considering the hydrolysed behaviour of these dual probes, cell viability assays were conducted under irradiation at 540 nm and 460 nm.

In the dark, none of the C-NAF derivatives showed any cytotoxicity (see Fig. S50 in SI). Under irradiation at 540 nm, C-NAF derivatives are not phototoxic, consistent with the negligible singlet oxygen generation observed for the intact C-NAF derivatives (*Φ*_Δ_ < 0.001). In contrast, hydrolysis leads to the formation of the corresponding NAF aldehyde derivatives, which display increased ROS-generating ability (Table 2). Therefore, the phototoxicity of the C-NAF derivatives in MCF-7 cells was also evaluated under irradiation at 460 nm, where the hydrolysis products absorb. As shown in Fig. 6A, significant phototoxicity was observed under irradiation at this wavelength, supporting that hydrolysis plays a fundamental role in the therapeutic effect of the probes. Specifically, C-NAF-B7 shows greater phototoxicity than the other C-NAF derivatives, which is likely related to the higher cellular uptake attributed to the biotin fragment, which interacts with membrane receptors that facilitate internalization, the specific mechanism responsible was not directly investigated in the present study, but has been supported in the literature.^18^

**Fig. 6:**
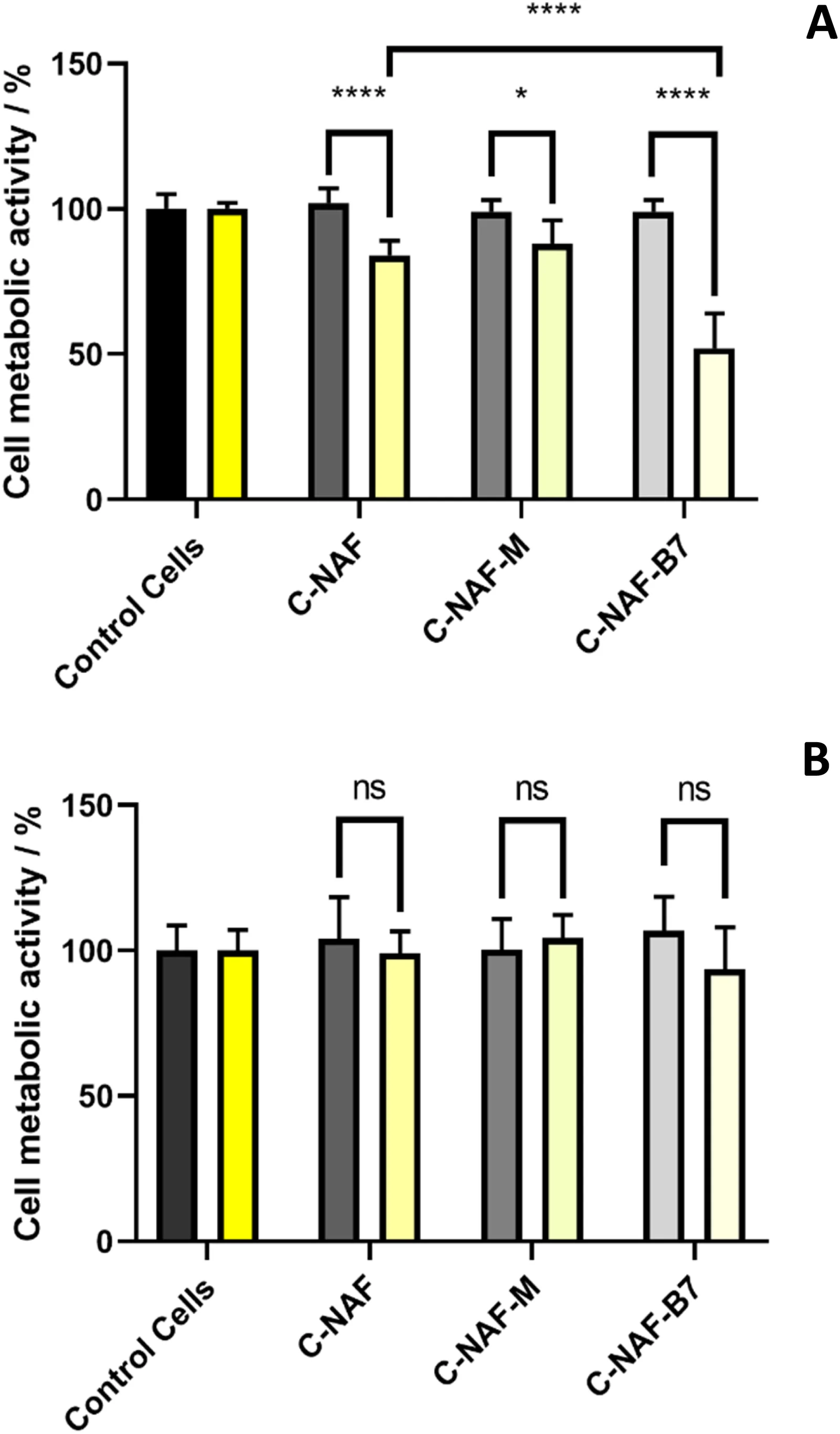
Dark toxicity (grey) and phototoxicity (yellow) of C-NAF derivatives (1.0 µM) on MCF-7 cancer cells (A) and HEK-293 non-neoplastic cells (B). Incubation for 90 min followed by irradiation with 460 nm LEDs for 60 min. Statistical significance of *p* < 0.0001 (****). NS: no significance.

To verify the relationship between hydrolysis and the observed phototoxicity, control experiments were performed using a shorter incubation time of 15 min, which was expected to limit the extent of probe hydrolysis compared with the 90 min incubation used in the standard phototoxicity assay for the C-NAF derivatives. These results show that a 15 min incubation does not induce phototoxicity, either in the dark or under 460 nm irradiation (see Fig. S51 in SI), whereas a pronounced phototoxic response was observed after the 90 min incubation (Fig. 6A). The hydrolysis kinetics provide additional insight into this incubation-time dependence. In specific, the experimentally determined half-lives of C-NAF, C-NAF-M, and C-NAF-B7 were 8.82, 19.81, and 13.95 min, respectively, under the hydrolysis conditions described above. Notably, these half-lives are comparable to the short 15 min incubation period used in the control experiment, after which no significant phototoxicity was observed. This finding suggests that, under the cellular assay conditions, a longer incubation period is required to allow sufficient formation and/or accumulation of the photoactive hydrolysis products to produce a detectable phototoxic response. Consistently, pronounced phototoxicity was observed after the 90 min incubation. Taken together, the hydrolysis kinetics and the incubation-time-dependent phototoxicity support the interpretation that formation of the photoactive NAF hydrolysis products contributes to the observed photodynamic activity.

The selectivity of the probes for cancer cells was tested by comparing their phototoxicity with a non-neoplastic cell model of HEK-293 cell line. Fig. 6B shows that both the cytotoxicity in the dark and phototoxicity of the C-NAF derivatives under 460 nm irradiation in HEK-293 cells are negligible. These results suggest that under the experimental conditions investigated, the C-NAF derivatives exhibited a preferential phototoxic response in MCF-7 cells compared with HEK-293 cells, thus suggesting their potential as photoactive probes for PDT.

To verify that the observed photocytotoxicity was associated with intracellular reactive oxygen species (ROS) production, ROS generation was tested using DCFH-DA as fluorescent probe both in the dark and irradiated with 460 nm LEDs. As shown in Fig. 7, dark control experiments showed little increase in fluorescence signal (Fig. 7A), while irradiation of MCF-7 cells treated with C-NAF derivatives produced a pronounced increase in intracellular fluorescence compared with untreated irradiated cells (Fig. 7B), confirming the presence of light-induced ROS generation.

**Fig. 7:**
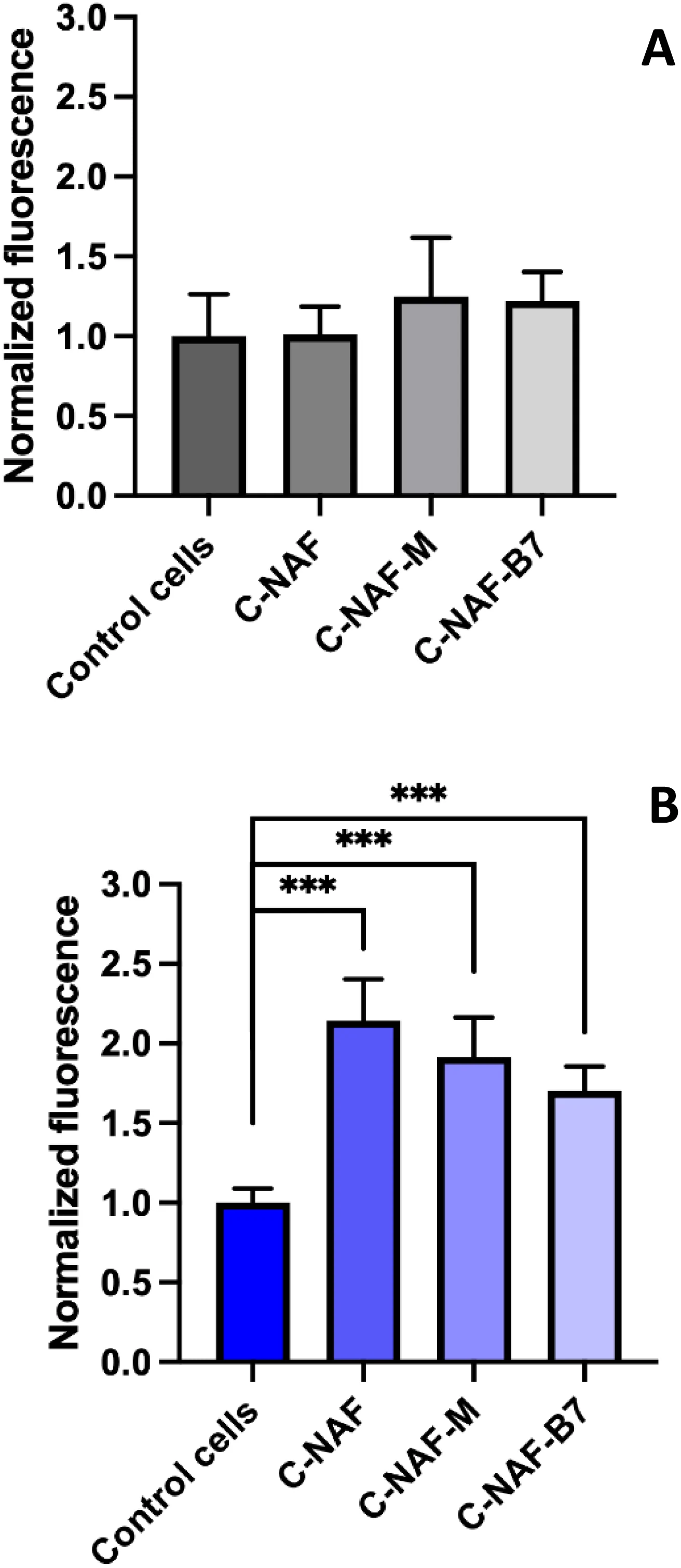
Intracellular ROS generation induced by C-NAF derivatives. Intracellular ROS levels were evaluated in MCF-7 cells using the DCFH-DA fluorescent probe after treatment with C-NAF, C-NAF-M, or C-NAF-B7 (1.0 µM) for 90 min, under dark conditions (A) or following irradiation with a 460 nm LED for 60 min (B). Fluorescence intensity was normalized to the corresponding control cells. Data is expressed as mean ± SD (*n* = 8). Statistical analysis was carried out using one-way ANOVA, and statistical significance was defined as *p* < 0.001 (***).

To identify the predominant ROS responsible for the photodynamic response, scavenger inhibition experiments were subsequently performed using sodium azide (NaN_3_), a widely known singlet oxygen (^1^O_2_) quencher, and Tiron, a known scavenger for superoxide anion radicals (O_2_˙^−^). These experiments were only performed for the C-NAF-B7 derivative, which showed the highest phototoxicity. The corresponding cell viability results are presented in Fig. 8. The addition of sodium azide did not modify the cell viability after irradiation compared to the control without azide, indicating that singlet oxygen is not the major cytotoxic species involved in the photodynamic response. In contrast, treatment with Tiron significantly recovered the cell viability, suggesting that superoxide radicals are major contributors to the photocytotoxic effect.

**Fig. 8:**
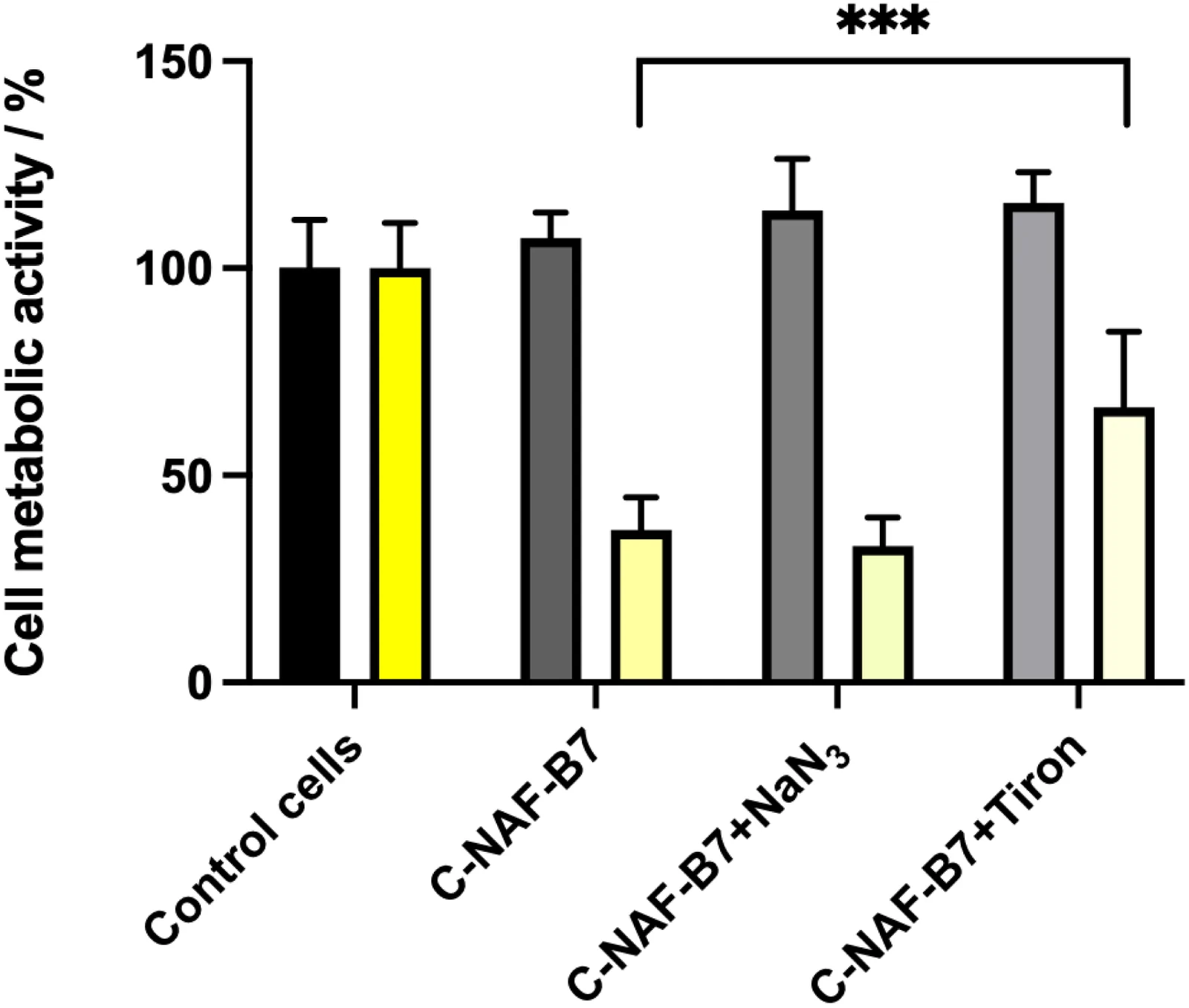
Cell metabolic activity of MCF-7 cells under dark (grey) and light (yellow) irradiation conditions treated with C-NAF-B7 (1.0 µM). Scavenger concentrations were 100 mM for both Tiron and sodium azide. Metabolic activity was assessed and expressed as a percentage relative to untreated control cells. Data are presented as mean ± standard deviation (SD) of *n* = 8 independent experiments. Statistical analysis was performed using one-way ANOVA followed by Tukey's *post hoc* test. Asterisks indicate statistically significant differences (***p < 0.001).

These results support a predominant contribution of radical-mediated ROS generation and are consistent with a type I photodynamic mechanism under the experimental conditions investigated. Although the involvement of secondary ROS derived from superoxide cannot be completely excluded, the marked protective effect of Tiron compared with the negligible effect of NaN_3_ indicates that singlet oxygen is unlikely to be the dominant cytotoxic ROS. Importantly, these biological observations are fully consistent with the photophysical characterization described above. C-NAF derivatives exhibit negligible singlet oxygen quantum yields (*Φ*_Δ_ < 0.001), whereas their hydrolysis products show increased but still relatively low *Φ*_Δ_ values. Thus, the combination of singlet oxygen measurements, intracellular ROS detection, and ROS-scavenging experiments supports radical-mediated photochemistry, rather than a predominantly type II pathway, as the major contributor to the observed photocytotoxicity.

## 3 Conclusions

In this work, we developed a family of coumarin–naphthalimide dual probes, combining NIR fluorescence with hydrolysis-activated photodynamic activity. The C-NAF derivatives exhibit broad NIR emission and pronounced sensitivity to microenvironmental polarity. Enhanced phototoxicity was observed after prolonged incubation, consistent with the proposed hydrolysis of C-NAF derivatives and subsequent formation of photoactive NAF species, as evidenced by the significant phototoxicity observed in MCF-7 cancer cells under 460 nm irradiation. ROS scavenger experiments indicate that a type I mechanism is the predominant pathway involved in cell death. Among the derivatives, C-NAF-B7 exhibited the strongest phototoxic response in MCF-7 cells, likely due to the presence of the biotin moiety may contribute to a better cellular recognition mediated by overexpressed biotin receptors in cancer cells. Moreover, its negligible toxicity in HEK-293 cells suggests they are more selective for tumoral cells. Overall, these results highlight the potential of C-NAF derivatives as promising dual probe that combine NIR fluorescence emission with phototoxic activity against cancer cells.

## 4 Materials and methods

### 4.1 Reagents

4-Bromo-1,8-naphthalic anhydride, butylamine, ethylnitroacetate, 4-(2-aminoethyl)-morpholine, *N*-hydroxysuccinimide (NHS), 1-[bis(dimethylamino)methylene]-1*H*-1,2,3-triazolo-[4,5-*b*] pyridinium-3-oxid hexafluorophosphate (HATU), *N*,*N*-diisopropylethylamine (DIPEA), *N*-Boc-ethylenediamine, trifluoroacetic acid (TFA), biotin (vitamin B_7_), were purchased from Sigma-Aldrich. 8-Hydroxyjulolidine-9-carboxaldehyde, hexamethylenetetramine (HMTA), *n-*butanol, sodium methoxide (CH_3_ONa), copper(ii) sulfate pentahydrate (CuSO_4_ × 5H_2_O), hydriodic acid 57%, tin(ii) chloride (SnCl_2_·2H_2_O), sodium hydroxide, anhydrous sodium sulfate (Na_2_SO_4_), potassium carbonate (K_2_CO_3_), dimethyl sulfoxide (DMSO) and chloroform were acquired from Merck. Methanol, ethanol, acetonitrile, dichloromethane, and *n*-hexane were purchased from J. T. Baker. All reagents and solvents were of analytical or HPLC grade.

### 4.2 Synthesis of coumarin probes

The products were characterized by ^1^H-NMR, and ultra-high-performance liquid chromatography (UHPLC) system coupled to a mass spectrometer (Ultimate 3000 RSLC system coupled to a Linear Ion Trap Mass Spectrometer LTQ XL, Thermo Scientific). The mass detection was carried out using electrospray ionization (SI), with the spray voltage set at 3 kV (350 °C). Detection was performed in a full scan mode in the 100–1000 *m*/*z* range in negative and positive mode.

The purity of the final products was determined by analytical chromatography (HPLC) equipped with a DAD detector and a Purospher STAR RP-18e (5 µm, 250 × 4 mm^2^) HPLC column. The sample was thermostated at 25 °C. The mobile phase consisted of acetonitrile, delivered isocratically at a flow rate of 1.0 mL min^−1^. The detection was performed at 220 nm and 550 nm, obtaining a purity yield *>*98% for all compounds (see Fig. S16, S26, and S39 in SI).

#### 4.2.1 Synthesis of coumarin–naphthalimide-butyl (1a, C-NAF)

The 4-formyl-*N*-butyl-1,8-naphthalimide (NAF) and coumarin amine (3, C-NH_2_), were synthesized as has been reported in the literature.^50–54^ The synthesis protocol and characterization are included in the SI (Schemes S1–S6 and Fig. S1, S12). The *N*-butylated naphthalimide precursor 2a (NAF, 98.13 mg, 0.30 mmol) and the coumarin 3 (43 mg, 0.2 mmol) were dissolved in ethanol (3 mL), and the solution was stirred at 80 °C for 6 h.^31,55^ After the reaction time, the solution was added to water, the solid formed was recovered by filtration and washed thoroughly with ethanol, and the solid was dried under vacuum for 24 h. 1a (Scheme S7 and Fig. S13–S16) was obtained as a purple solid (yield: 80%) with a melting point of 180–190 °C. ^1^H-NMR (400 MHz, CDCl_3_) *δ* 15.81 (d, *J* = 9.3 Hz, 1H), 9.16 (d, *J* = 9.5 Hz, 1H), 8.71 (dd, *J* = 8.0, 1.3 Hz, 1H), 8.58 (d, *J* = 7.5, 1.3 Hz, 1H), 8.40 (s, 1H), 7.64 (t, *J* = 7.8 Hz, 1H), 7.56 (s, 1H), 6.91 (s, 1H), 4.19–4.12 (m, 2H), 3.33 (m, 4H), 2.90 (t, *J* = 6.2 Hz, 2H), 2.79 (t, *J* = 6.2 Hz, 2H), 2.01 (m, 4H), 1.70 (m, 2H), 1.47–1.41 (m, 2H), 0.98 (t, *J* = 7.4 Hz, 3H). ^13^C NMR (100.13 MHz, CDCl_3_) *δ* 177.62, 164.40, 163.60, 157.80, 156.09, 149.94, 146.80, 137.72, 133.15, 131.94, 131.19, 131.02, 127.46, 125.68, 125.35, 122.58, 119.88, 117.51, 111.70, 110.72, 107.42, 106.48, 50.17, 49.75, 40.08, 30.32, 27.51, 21.18, 20.44, 20.26, 20.23, 13.89. UHPLC-MS/MS [M − H]^+^: *m*/*z* 536.33.

#### 4.2.2 Synthesis of coumarin–naphthalimide–morpholine (1b, C-NAF-M)

The 4-formyl-*N*-morpholine-1,8-naphthalimide (NAF-M) was synthesized as reported in literature,^56,57^ the synthesis protocol and characterization are described in the SI (Schemes S8–S10 and Fig. S17–S22). The *N*-morpholinyl naphthalimide precursor 2b (NAF-M, 100 mg, 0.168 mmol) and the coumarin 3 (30.72 mg, 0.12 mmol) were dissolved in ethanol (15 mL), and the solution was refluxed for 3 h.^31,55^ The excess solvent was removed under reduced pressure, and the product was purified with a silica gel chromatography column, with dichloromethane 90% and 10% methanol. The solid was dried under vacuum for 24 h. 1b (Scheme S11 and Fig. S23–S26) was obtained as a purple solid (yield: 60%) with a melting point of 200–210 °C. ^1^H-NMR: ^1^H-NMR (400 MHz, CDCl_3_) *δ* 13.35 (s, 1H) *δ* 7.98 (d, 1H), 7.83 (s, 1H), 7.65 (d, 1H), 7.56 (s, 1H), 7.31 (t, 1H), 7.05 (s, 1H), 6.90 (s, 1H), 5.30 (s, 1H), 4.34 (d, 4H), 3.47 (d, 2H), 3.33 (m, 4H), 2.89 (t, 4H), 1.90 (s, 2H), 1.22–1.08 (m, 4H), 0.88 (t, 4H). ^13^C NMR (100.13 MHz, CDCl_3_) *δ* (ppm): 166.29, 165.47, 164.33, 163.86, 163.59, 139.88, 135.28, 133.90, 131.51, 129.72, 128.27, 127.10, 126.00, 125.48, 123.06, 122.28, 119.75, 115.22, 112.27, 109.80, 63.93, 57.48, 55.62, 53.42, 52.47, 37.38, 33.78, 29.71. UHPLC MS/MS: [M + H]^+^: 593.39.

#### 4.2.3 Synthesis of coumarin–naphthalimide–biotin (1c, C-NAF-B7)

The 4-formyl-*N*-biotin-1,8-naphthalimide (NAF-B7) was synthesized by adapting previously reported biotin-functionalization procedures reported in literature,^58,59^ (see in Schemes S12–S16 and Fig. S27–S34). The *N*-biotinyl naphthalimide 2c (NAF-B7, 100 mg, 0.168 mmol) and C-NH_2_ (30.72 mg, 0.12 mmol) were dissolved in ethanol (15 mL) and was refluxed for 4 h. Then excess solvent was removed under reduced pressure, and the product was purified with a silica chromatography column with CH_2_Cl_2_/MeOH, increasing from 5% to 10% methanol. After that, solvent was removed in rotary evaporation, and the solid was dried under vacuum for 24 h. 1c (Scheme S17 and Fig. S36–S39). It was obtained as a purple solid (yield: 40%) with a melting point of 220–225 °C. ^1^H-NMR (400 MHz, DMSO) *δ* 14.14 (s, 1H), *δ* 9.15 (s, 1H), 8.61 (s, 1H), 8.39 (m, 2H), 8.19 (s, 1H), 7.91–7.66 (m, 2H), 6.76 (s, 1H), 4.29 (m, 2H), 4.12 (m, 4H), 3.15 (m, 7H), 2.85–2.60 (m, 6H), 2.00 (s, 2H), 1.90 (m, 4H), 1.45 (m, 4H), 1.26 (m, 2H). UHPLC MS/MS: [M − H]^+^: 747.46.

### 4.3 Absorption and steady-state fluorescence measurements

C-NAF derivatives solutions were prepared at 1.25 µM concentration in methanol, dimethyl sulfoxide, dichloromethane, and chloroform. Absorption spectra were recorded using a UV-visible Agilent 8453 spectrophotometer at room temperature in air-equilibrated solutions. Steady-state fluorescence measurements were carried out using an LS55 PerkinElmer spectrofluorometer, exciting at the maximum absorption in each solvent. Fluorescence quantum yields were determined using the area under the curve (AUC) of the fluorescence spectra and the absorbance at the excitation wavelength in each solvent for the sample (*x*) and a standard (std) (eqn (1)). Bengal rose in methanol (*φ*_std_ = 0.08) was used as a standard compound for C-NAF derivatives.^33,35,60^ The fluorescence measurements were recorded using the sample and standard with absorbance values lower than 0.1 at the excitation wavelength to avoid inner-filter effects. The values were corrected by the refractive index (*η*) for the sample (*η*_x_) and standard (*η*_std_) in each solvent.1
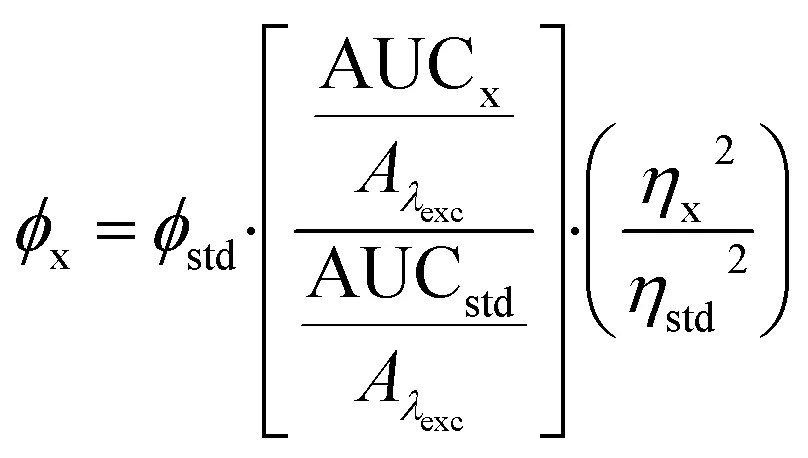


### 4.4 Hydrolysis analysis of C-NAF derivatives

The kinetics of the hydrolysis of C-NAF derivatives in methanol containing 0.02% water were evaluated from the decrease of the absorbance band between 530–540 nm through the use of UV-visible spectroscopy (UV-visible Agilent 8453 Spectrophotometer) as a function of time, under aerobic conditions.^61,62^ The absorbance values obtained as a function of time were fitted by nonlinear regression assuming pseudo-first-order hydrolysis kinetics, using the following monoexponential decay eqn (2)

2 *A*_*t*_ = *A*_∞_ + (*A*_0_ − *A*_∞_)e^−*k*_obs_*t*^

where *A*_*t*_ is the absorbance measured at time *t*, *A*_0_ is the initial absorbance at *t* = 0, *A*_∞_ is the absorbance reached at long reaction times (plateau), and *k*_obs_ is the observed pseudo-first-order hydrolysis rate constant (min^−1^). The hydrolysis half-life (*t*_1/2_) of each C-NAF derivative was subsequently calculated from the fitted *k*_obs_ value using eqn (3):3
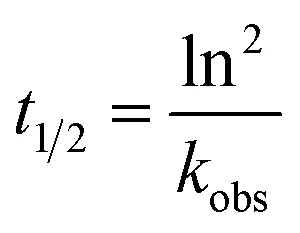
where *t*_1/2_ is expressed in minutes when *k*_obs_ is expressed in min^−1^. The hydrolysis products were confirmed by mass spectrometry, using a UHPLC Ultimate 3000 RSLC coupled with an LTQ XL linear Ion Trap Mass Spectrometer (Thermo Scientific). Briefly, aliquots from the hydrolysis reaction in methanol were diluted in 0.1% formic acid and immediately injected into UHPLC-MS/MS employing a Hypersil GOLD C18 column (50 × 4.6 mm, 1.9 mm, Thermo Fisher Scientific). An isocratic mobile phase (30/70) 0.1% formic acid/acetonitrile (0.1% formic acid) at 0.4 mL min^−1^ was employed and monitored for 10 min.

### 4.5 Singlet oxygen quantum yields

The singlet oxygen quantum yields (*Φ*_Δ_) of the C-NAF derivatives and their corresponding formyl-naphthalimide hydrolysis products (NAF) were determined in ultrapure water using 9,10-anthracenediyl-bis(methylene)dimalonic acid (ABMA) as a chemical probe for singlet oxygen. Singlet oxygen production was quantified by monitoring the decrease in the ABMA absorbance upon irradiation of the photosensitizers. Quantum yields were calculated by the relative method using rose bengal (*Φ*_Δ_ = 0.70 in water) as the reference photosensitizer for the C-NAF derivatives irradiated at 525 nm and riboflavin (*Φ*_Δ_ = 0.61 in water) as the reference for the NAF derivatives irradiated at 450 nm.^63^ The rate of ABMA consumption was determined by following the absorbance at 380 nm during irradiation at 450 nm and 525 nm LED-Illuminator (LEDi, Luzchem). The measurements were expressed as singlet oxygen quantum yields (*Φ*_Δ_) values calculated from the slopes of first-order plots as shown in eqn (4).^33,64^4
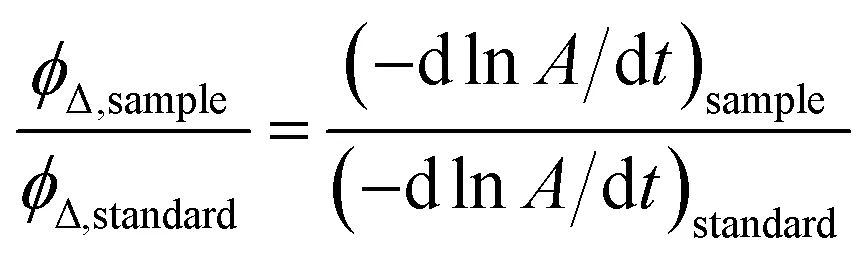


### 4.6 Structure optimization and electronic absorption spectra

The ground-state calculation was performed using density functional theory (DFT) with the Gaussian09 package.^65^ Stationary points on the potential energy surface were obtained using the B3LYP hybrid density functional.^45,66^ The molecular geometry optimization of C-NAF derivatives was performed in water using the 6-31G(d,p) basis set for all atoms.^67,68^ Analytical computations of harmonic vibrational frequencies verified the converged wavefunctions. Time-dependent DFT (TD-DFT) was used to study the electronic absorption spectra for each molecular system.^69,70^ The functional B97D was chosen based on its excellent performance in a previous study for coumarin derivates by TD-DFT calculations. The UV-visible spectra were obtained by singlet–singlet vertical transitions of Franck–Condon type using 30 excited states. The absorption spectra were calculated in the solution phase (methanol) using the conductor-like polarizable continuum model (C-PCM).^71^

### 4.7 *In vitro* cell culture studies

Human embryonic kidney cells HEK-293 (ATCC® CRL-1573), used as a healthy model, and MCF-7 adenocarcinoma breast cancer cells (ATCC® HTB-22) were cultured in Dulbecco's Modified Eagle's Medium (DMEM) supplemented with 10% fetal bovine serum (FBS), 100 U mL^−1^ penicillin, and 100 µg mL^−1^ streptomycin at 37 °C, 5% CO_2_, and 100% humidity. Cell cytotoxicity and phototoxicity assays were conducted in 96-well plates, with each measurement performed in octuplicate. Solutions of 1a–c derivatives were prepared in dimethyl sulfoxide (DMSO), and stock solutions were diluted to the final concentration using non-supplemented DMEM without phenol red. The final concentration used in cellular experiments was 1.0 µM. In all cases, the DMSO content in the final solutions was maintained below 1% (v/v). Cell culture plates contained 100 µL of MCF-7 or HEK 293 cells at a concentration of 1.5 × 10^5^ cells per mL in DMEM. After overnight incubation, the culture medium was removed, and the cells were rinsed once with phosphate-buffered saline (PBS, 100 mM, pH 7.4). Subsequently, 100 µL of 1a, 1b, and 1c solutions were added to the cells and incubated for 90 min at 37 °C, 5% CO_2_, and 100% humidity. Following incubation, the medium was removed, the cells were washed once with PBS, and fresh DMEM without phenol red supplementation was added. Identical plates were prepared; one set was maintained in darkness, while the other was exposed to illumination for 60 min using a Luzchem LED-L16 photoreactor (460 and 540 nm, light intensity of 6.27 mW cm^−2^) at 37 °C. Finally, the medium was removed, replaced with supplemented DMEM, and the cells were incubated for an additional 24 h.^34,40^

A colorimetric MTS assay (3-(4,5-dimethylthiazol-2-yl)-5-(3-carboxymethoxyphenyl)-2-(4-sulfophenyl)-2*H*-tetrazolium) was employed to determine the cytotoxicity/phototoxicity effect of 1a–c derivatives on MCF-7 and HEK-293 cells. 10 µL of MTS (5 mg mL^−1^) was added to each well and incubated for 2 h at 37 °C. Afterward, cell viability was determined by measuring the absorbance of each well at 490 nm using a microplate reader Biotek Synergy HT. GraphPad Prism 9® was employed to analyze the data, using the one-way ANOVA test to determine the statistical significance. The data were considered statistically significant with a confidence interval of *p* < 0.05.

#### 4.7.1 Intracellular reactive oxygen species detection

Intracellular ROS production was evaluated in MCF-7 cells using dichlorodihydrofluorescein diacetate (DCFH-DA) as a fluorescent probe. Cells were seeded in 96-well plates at a density of 1.5 × 10^5^ cells per mL (100 µL per well) and incubated overnight at 37 °C in a humidified atmosphere containing 5% CO_2_.

Sterile working solutions were prepared in phenol red-free DMEM. Cells were incubated with 100 µL of DCFH-DA (10 µM) for 30 min at 37 °C, washed with PBS, and subsequently treated with all the compounds at 1.0 µM concentrations for 90 min. The plates were then divided into two experimental conditions. For the light condition, cells were irradiated for 60 min at 460 nm under the same conditions described above for phototoxicity experiments. In parallel, control plates were maintained in the dark for the same period under identical experimental conditions. Immediately after irradiation, fluorescence emission was measured using a Cytation 5 Cell Imaging Multi-Mode Reader at excitation and emission wavelengths of 430 nm and 535 nm, respectively. Data was analyzed using GraphPad Prism 9® by one-way ANOVA, with statistical significance established at *p* < 0.05.

#### 4.7.2 Reactive oxygen species scavenging experiments

The contribution of reactive oxygen species to the phototoxic effect was investigated using Tiron as a superoxide anion scavenger and sodium azide as a singlet oxygen quencher. Cells were treated with 1.0 µM of C-NAF-B7 in the absence or presence of either Tiron (100 mM) or sodium azide (100 mM) for 90 min at 37 °C under a humidified atmosphere containing 5% CO_2_. After incubation, the cells were washed, irradiated under the same conditions used in the phototoxicity assay, and subsequently incubated as previously described.

## Author contributions

Benjamín Zúñiga-Salazar: formal analysis, data curation. Paula S. Rivero-Jerez: formal analysis, data curation, writing – review & editing. Sofía Pérez-Del Pino: formal analysis, data curation. Francisco Mura: methodology, formal analysis, conceptualization. Pablo Barrias: formal analysis, data curation. Martín Canals Díaz: formal analysis, data curation. José Robinson-Duggon: conceptualization, formal analysis, methodology. Alexis Aspée: writing – review & editing, resources, formal analysis, conceptualization. Denis Fuentealba: writing – original draft, resources, methodology, investigation, formal analysis, conceptualization. Daniel Zúñiga-Núñez: writing – original draft, resources, methodology, investigation, formal analysis, conceptualization.

## Conflicts of interest

There are no conflicts to declare.

## Supplementary Material

RA-016-D6RA08240H-s001

## Data Availability

All data this study are provided in the supplementary information (SI), including synthesis and characterization of the compound. Additional data are available from the corresponding author upon reasonable request. Supplementary information is available. See DOI: https://doi.org/10.1039/d6ra08240h.
