# Supplementary material for: Design and synthesis of hydrolyzable coumarin–naphthalimide dual probes for NIR fluorescence detection and selective photodynamic activity in cancer cells
Source: RSC Adv. 2026 Oct 19;16(48):64171–86. doi: 10.1039/d6ra08240h (PMC13678496; doi:10.1039/d6ra08240h)
Supplement: Supplementary file 1 [file RA-016-D6RA08240H-s001.pdf]

Supplementary Information (SI) for RSC Advances.
This journal is © The Royal Society of Chemistry

# Electronic Supplementary Information (ESI)

## Design and Synthesis of Hydrolyzable Coumarin–Naphthalimide Dual Probes for NIR Fluorescence Detection and Selective Photodynamic Activity in Cancer Cells

Benjamín Zúñiga-Salazar,[a] Paula S. Rivero-Jerez,[b] Sofía Pérez-Del Pino,[b] Francisco Mura,[a] Pablo Barrias,[c] Martin Canals Díaz, [b] José Robinson-Duggon,[d,e] Alexis Aspée,[a] Denis Fuentealba*,[b], and Daniel Zúñiga-Núñez,*,[a]

[a] *Laboratorio de Cinética y Fotoquímica, Facultad de Química y Biología, Universidad de Santiago de Chile, Santiago, Chile*

[b] *Laboratorio de Química Supramolecular y Fotobiología, Departamento de Química Física, Escuela de Química, Facultad de Química y de Farmacia, Pontificia Universidad Católica de Chile, Santiago, Chile*

[c] *Universidad Mayor, Facultad de Ciencias, Ingeniería y Tecnología, Centro de Nanotecnología Aplicada, Camino La Pirámide 5750, Huechuraba 8580745, Santiago, Chile*

[d] *Universidad de Panamá, Facultad de Ciencias Naturales, Exactas y Tecnología, Departamento de Bioquímica, Panamá 0824, República de Panamá*

[e] *Sistema Nacional de Investigación (SNI), Secretaría Nacional de Ciencia, Tecnología e Innovación (SENACYT), Panamá, República de Panamá*

*Correspondence: daniel.zunigan@usach.cl

dlfuente@uc.cl

# Contents

**S1. Synthesis methodology of precursors and C-NAF derivatives ........ 6**

S1.1 Coumarin amine ........ 6

Scheme S1. Synthesis compound 5. ........ 6

Scheme S2. Synthesis compound 3. ........ 6

S1.2 C-NAF derivatives and C-NAF ........ 7

Scheme S3. Synthesis compound 7. ........ 7

Scheme S4. Synthesis compound 8. ........ 7

Scheme S5. Synthesis compound 9. ........ 8

Scheme S6. Synthesis compound 2a (NAF). ........ 8

Scheme S7. Synthesis of 1a (C-NAF). ........ 9

S1.3 Naphthalimide-Morpholine (NAF-M) ........ 10

Scheme S8. Synthesis of compound 10. ........ 10

Scheme S9. Synthesis compound 11. ........ 11

Scheme S10. Synthesis of compound 2b (NAF-M). ........ 12

Scheme S11. Synthesis of compound 1b (C-NAF-M). ........ 13

S1.4 Naphthalimide-Biotin (NAF-B7) ........ 14

Scheme S12. Synthesis compound 12. ........ 14

Scheme S13. Synthesis compound 13. ........ 15

Scheme S14. Synthesis of compound 14. ........ 16

Scheme S15. Synthesis compound 15. ........ 17

Scheme S16. Synthesis of compound 2c (NAF-B7). ........ 18

Scheme S17. Synthesis of compound 1c (C-NAF-B7). ........ 19

**S2. NMR and UHPLC-MS/MS characterization ........ 20**

Figure S1. $^{1}$H NMR in $CDCl_3$ of compound 5. ........ 20

Figure S2. UHPLC MS/MS in positive mode of compound 5. ........ 20

Figure S3. $^{1}$H NMR in $CDCl_3$ of compound 3. ........ 21

Figure S4. UHPLC MS/MS in positive mode of compound 3. ........ 21

Figure S5. $^{1}$H NMR in $CDCl_3$ of compound 7. ........ 22

Figure S6. UHPLC MS/MS in positive mode of compound 7. ........ 22

Figure S7. $^{1}$H NMR in $CDCl_3$ of compound 8. ........ 23

Figure S8. UHPLC MS/MS in positive mode of compound 8. .......... 23

Figure S9. $^{1}$H NMR in DMSO-$d_6$ of compound 9. .......... 24

Figure S10. UHPLC MS/MS in positive mode of compound 9. .......... 24

Figure S11. $^{1}$H NMR in DMSO-$d_6$ of compound 2a (NAF). .......... 25

Figure S12. UHPLC MS/MS in positive mode of compound 2a (NAF). .......... 25

Figure S13. $^{1}$H NMR in $CDCl_3$ of 1a (C-NAF). .......... 26

Figure S14. $^{13}$C NMR in $CDCl_3$ of 1a (C-NAF). .......... 27

Figure S15. UHPLC MS/MS in positive mode of 1a (C-NAF). .......... 27

Figure S16. Chromatogram of C-NAF obtained by HPLC-DAD. The sample was thermostated at 25 °C and analyzed under isocratic conditions using 100% acetonitrile as the mobile phase at a flow rate of 1.0 mL min$^{-1}$. Top: detection at 550 nm; bottom: detection at 220 nm. .......... 28

Figure S17. $^{1}$H NMR in DMSO-$d_6$ of compound 10. .......... 29

Figure S18. UHPLC MS/MS in positive mode of compound 10. .......... 29

Figure S19. $^{1}$H NMR in DMSO-$d_6$ of compound 11. .......... 30

Figure S20.UHPLC MS/MS in positive mode of compound 11. .......... 30

Figure S21. $^{1}$H NMR in DMSO-$d_6$ of compound 2b (NAF-M). .......... 31

Figure S22. UHPLC MS/MS in positive mode of compound 2b (NAF-M). .......... 31

Figure S23. $^{1}$H NMR in $CDCl_3$ of compound 1b (C-NAF-M). .......... 32

Figure S24. $^{13}$C NMR in $CDCl_3$ of compound 1b (C-NAF-M). .......... 32

Figure S25. UHPLC MS/MS in positive mode of compound 1b (C-NAF-M). .......... 33

Figure S26. Chromatogram of C-NAF-M obtained by HPLC-DAD. The sample was thermostated at 25 °C and analyzed under isocratic conditions using 100% acetonitrile as the mobile phase at a flow rate of 1.0 mL min$^{-1}$. Top: detection at 550 nm; bottom: detection at 220 nm. .......... 34

Figure S27. $^{1}$H-NMR in $CDCl_3$ of compound 12. .......... 35

Figure S28. $^{1}$H NMR in DMSO-$d_6$ of compound 13. .......... 36

Figure S29. UHPLC MS/MS in positive mode of compound 13. .......... 36

Figure S30. $^{1}$H NMR in DMSO-$d_6$ of compound 14. .......... 37

Figure S31. UHPLC MS/MS in positive mode of compound 14. .......... 37

Figure S32. $^{1}$H-NMR in DMSO-$d_6$ of compound 15. .......... 38

Figure S33. UHPLC MS/MS in positive mode of compound 15. .......... 38

Figure S34. 1H NMR in DMSO-d6 of compound 2c, NAF-B7. .......... 39

Figure S36. $^{1}$H-NMR in DMSO-$d_6$ of compound 1c (C-NAF-B7). .......... 40

Figure S38. UHPLC MS/MS in positive mode of compound 1c (C-NAF-B7). .................................... 41

Figure S39. Chromatogram of C-NAF-B7 obtained by HPLC-DAD. The sample was thermostated at 25 °C and analyzed under isocratic conditions using 100% acetonitrile as the mobile phase at a flow rate of 1.0 mL min$^{-1}$. Top: detection at 550 nm; bottom: detection at 220 nm. ............................................ 42

**S3. Additional electronic and photophysical properties ........................................................ 43**

S3.1 Molecular orbital frontiers of C-NAF and C-NAF-M ............................................................ 43

Figure S40. Molecular orbital surfaces of the LUMO (A) and HOMO (B) of C-NAF, calculated at the B3LYP/6-31G(d,p) level of theory in methanol. Solvent effects were modeled using the C-PCM approach. ........................................................................................................................................... 43

Figure S41. Molecular orbital surfaces of the LUMO (A) and HOMO (B) of C-NAF-M, calculated at the B3LYP/6-31G(d,p) level of theory in methanol. Solvent effects were modeled using the C-PCM approach. ........................................................................................................................................... 44

S3.2 Absorption spectra of NAF derivatives and Coumarin amine ............................................... 45

Figure S42. Normalized absorption spectra of NAF derivatives and coumarin amine in methanol. NAF (-), C-NAF-M (-), C-NAF-B7 (-) and C-$NH_2$ (-). ........................................................................ 45

**S4. Identification of hydrolysis products by UHPLC-MS/MS and kinetics studies ....................... 46**

S4.1 Mass Spectrum of hydrolysis of C-NAF .................................................................................. 46

Figure S43. Chromatogram and mass spectrum of C-NAF hydrolysis in methanol determined by UHPLC MS/MS, positive mode. ...................................................................................................... 46

S4.2 Mass Spectrum of hydrolysis of C-NAF-M ............................................................................. 47

Figure S44. Chromatogram and mass spectrum of C-NAF-M hydrolysis in methanol determined by UHPLC MS/MS, positive mode. ...................................................................................................... 47

S4.3 Mass Spectrum of hydrolysis of C-NAF-B7 ............................................................................. 48

Figure S45. Mass spectrum of C-NAF-B7 hydrolysis in methanol determined by direct injection in UHPLC MS/MS, positive mode. ...................................................................................................... 48

S4.4. Hydrolysis studies and kinetic analysis ................................................................................ 49

**S5. Singlet oxygen generation and ROS-related experiments .................................................... 50**

Figure S47. Consumption of ABMA (200 mM) by irradiation LED of NAF derivatives (2.5 μM) at 460 nm in ultrapure water. NAF (A), NAF-M (B), NAF-B7 (C) and C-$NH_2$ (D) .................................................. 50

**S6. Stability under biologically relevant conditions ................................................................. 51**

S6.1 Fluorescence stability in DMEM conditions ........................................................................... 51

Figure S48. Normalized fluorescence emission intensity ($I/I_0$) of C-NAF (black), C-NAF-M (red), and C-NAF-B7 (blue) in DMEM under continuous 540 nm irradiation for 30 min. Fluorescence intensity was normalized to the initial value ($I_0$). The gradual decrease in emission intensity indicates photoinduced hydrolytic degradation of the C-NAF derivatives under prolonged irradiation. .............................. 51

S6.2 Evaluation of the spectral stability of the C-NAF derivatives at different pH values ............... 52

Figure S49. Effect of pH on the absorption spectra of the C-NAF derivatives. Normalized UV–Vis absorption spectra of (A) C-NAF, (B) C-NAF-M, and (C) C-NAF-B7 recorded at pH 5.0 (black), pH 7.4 (red), and pH 8.0 (blue). ........................................................................................................ 52

**S7. In vitro cytotoxicity and phototoxicity controls of C-NAF derivatives in MCF-7 .................... 53**

Figure S50. Dark toxicity (grey) and phototoxicity (yellow) of C-NAF derivatives (1.0 μM) on MCF-7 cells. Incubation for 90 min followed by irradiation with 540 nm LEDs for 60 min. NS: no significance... 53

Figure S51. Dark toxicity (grey) and phototoxicity (yellow) of C-NAF derivatives (1.0 μM) on MCF-7 cells. Incubation for 15 min followed by irradiation with 460 nm LEDs for 60 min. Statistical significance of $p < 0.0001$ (****). ........................................................................................................ 54

## S1. Synthesis methodology of precursors and C-NAF derivatives

### S1.1 Coumarin amine

8-hydroxyjuliolidine-9-carboxaldehyde (500 mg, 2.3 mmol) and ethyl nitroacetate (306 mg, 2.3 mmol) were dissolved in 10 mL of *n*-butanol. Three drops of piperidine were added, and the reaction mixture was refluxed with stirring for 12 h. After completion, the mixture was cooled to room temperature and left to stand for 24 h. The resulting solid was collected by filtration to afford a purple product (431.6 mg, 66% yield). $^{1}$H NMR: (400 MHz, $CDCl_3$) δ 8.53 (s, 1H), 6.96 (s, 1H), 3.41 (q, 4H), 2.90 – 2.82 (m, 2H), 2.81 – 2.73 (m, 2H). UHPLC MS/MS: $[M+H]^+$:287.08.

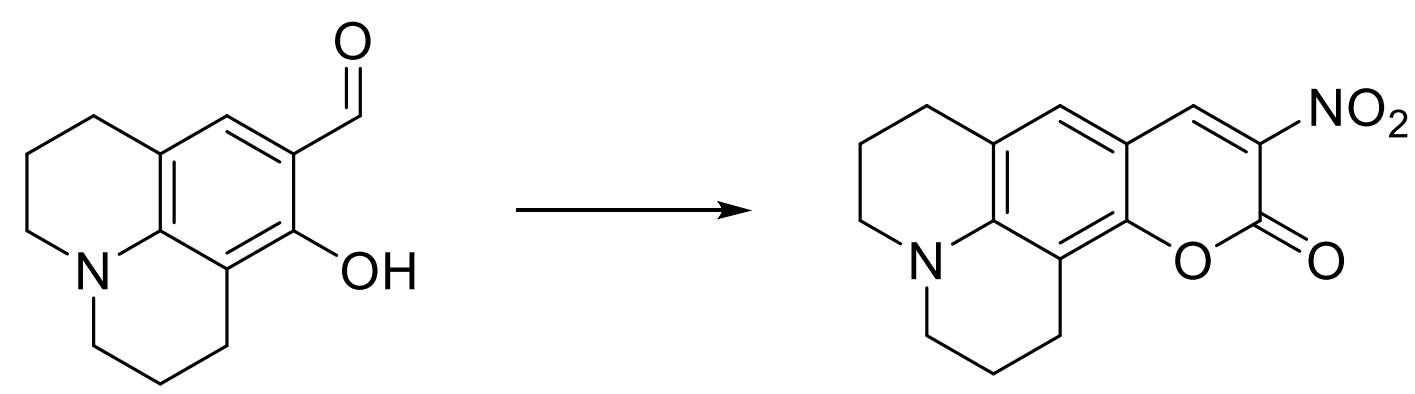


4

Chemical Formula: $C_{13}H_{15}NO_2$
Exact Mass: 217,11

5

Chemical Formula: $C_{15}H_{14}N_2O_4$
Exact Mass: 286,10

**Scheme S1. Synthesis compound 5.**

In a 25 mL round-bottom flask, concentrated HCl (37%) and Tin (II) chloride dihydrate (603 mg, 2.67 mmol) were combined. Subsequently, coumarin-nitro (**5**, 255 mg, 0.890 mmol) was added portion wise over 30 min at room temperature. The reaction mixture was then stirred for 5 h. Upon completion, the mixture was cooled in an ice bath and neutralized with 5 M NaOH. The resulting precipitate was collected by filtration to afford a brown solid (**3**, 180 mg, 79% yield). $^{1}$H NMR (400 MHz, DMSO) δ 7.40 (s, 1H), 7.01 (s, 1H), 3.22 (p, *J* = 3.6 Hz, 4H), 2.77 – 2.67 (m, 1H), 1.95 – 1.81 (m, 1H). UHPLC MS/MS: $[M+H]^+$:257.01

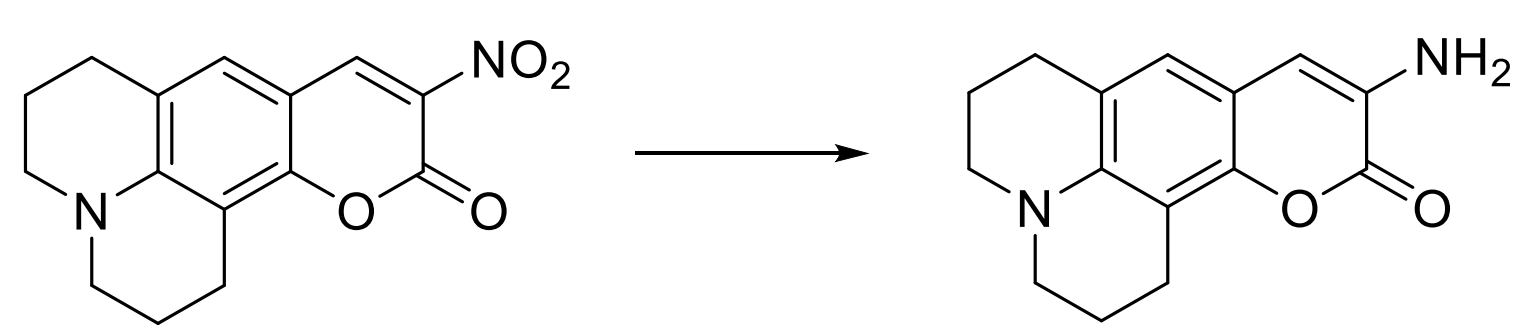


5

Chemical Formula: $C_{15}H_{14}N_2O_4$
Exact Mass: 286,10

3

Chemical Formula: $C_{15}H_{16}N_2O_2$
Exact Mass: 256,12

**Scheme S2. Synthesis compound 3.**

## S1.2 C-NAF derivatives and C-NAF

4-Bromo-1,8-naphthalic anhydride (**6**, 3.98 g, 14.43 mmol) was dissolved in ethanol (80 mL), and butylamine (1.005 g, 14.43 mmol) was added. The reaction mixture was stirred in the refluxing ethanol for 24h. After cooling to room temperature, the mixture was filtered and washed with ethanol 3 times and dried under reduced pressure to yield (4.60 g, 96%) as a yellow solid. $^{1}H$ NMR (400 MHz, $CDCl_3$) δ 8.67 (m, 1H), 8.59 – 8.55 (m, 1H), 8.41 (d, *J* = 7.9 Hz, 1H), 8.04 (d, *J* = 7.9 Hz, 1H), 7.85 (m, 1H), 4.24 – 4.11 (m, 2H), 1.71 (m, 2H), 1.45 (m, 2H), 0.98 (t, *J* = 7.4 Hz, 3H); UHPLC MS/MS: $[M+H]^+$:332.07.

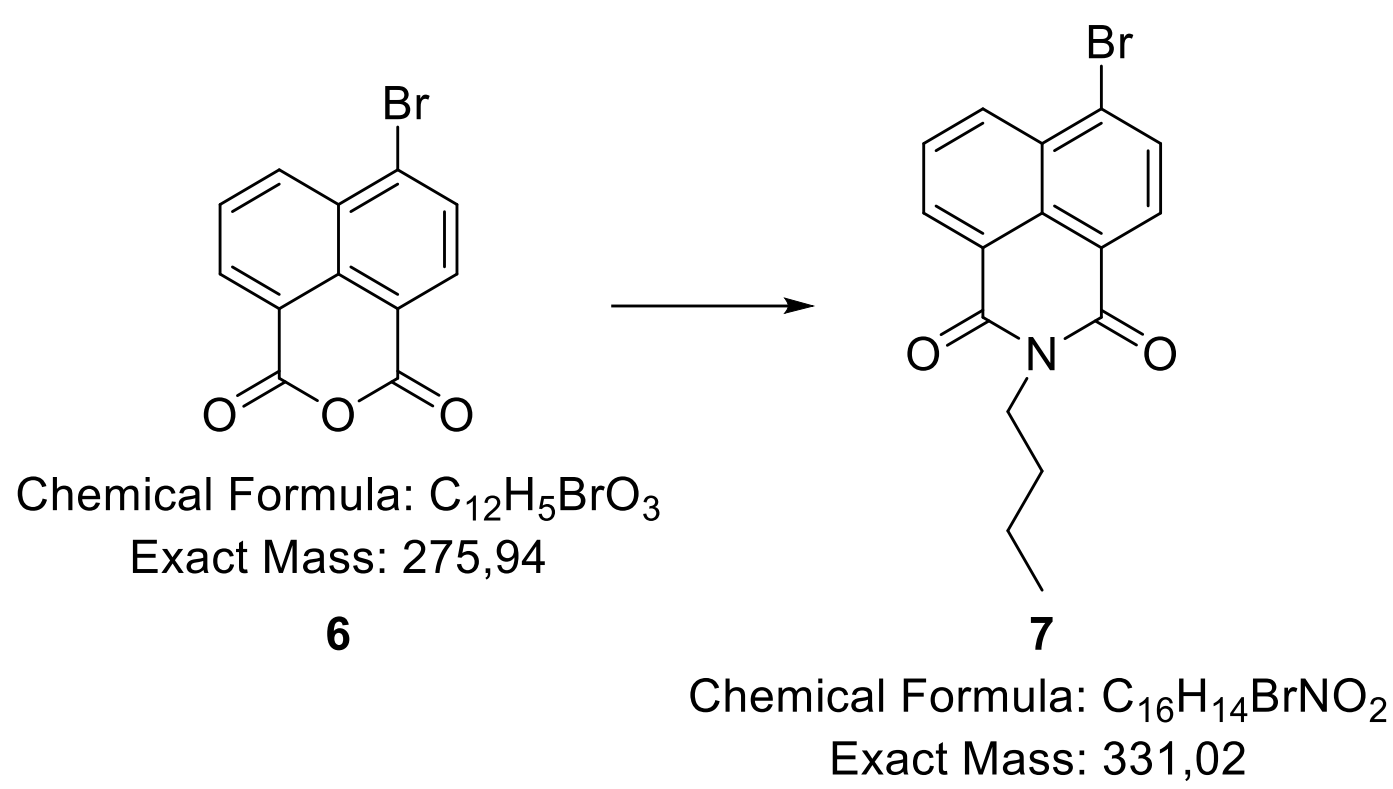


**Scheme S3. Synthesis compound 7.**

A mixture of compound **7** (540 mg, 1.63 mmol), $CH_3ONa$ (702 mg, 13.0 mmol) and $CuSO_4.5H_2O$ (50 mg, 0.2 mmol) in 5 mL $CH_3OH$ were refluxed for 8 hrs. After the removal of the solvent under reduced pressure and washing of the residue with water (30 mLx3). The compound **8** was dried under vacuum leading to yellow needles (415 mg, 90%). $^{1}H$ NMR (400 MHz, $CDCl_3$) δ 8.62 – 8.57 (m, 1H), 8.54 (d, *J* = 8.2 Hz, 1H), 7.74 – 7.64 (m, 1H), 7.03 (d, *J* = 8.3 Hz, 1H), 4.19 – 4.12 (m, 2H), 4.11 (s, 3H), 1.80 – 1.63 (m, 2H), 1.43 (m, 2H), 0.96 (t, *J* = 7.4 Hz, 3H). ; UHPLC MS/MS: $[M+H]^+$:264.16.

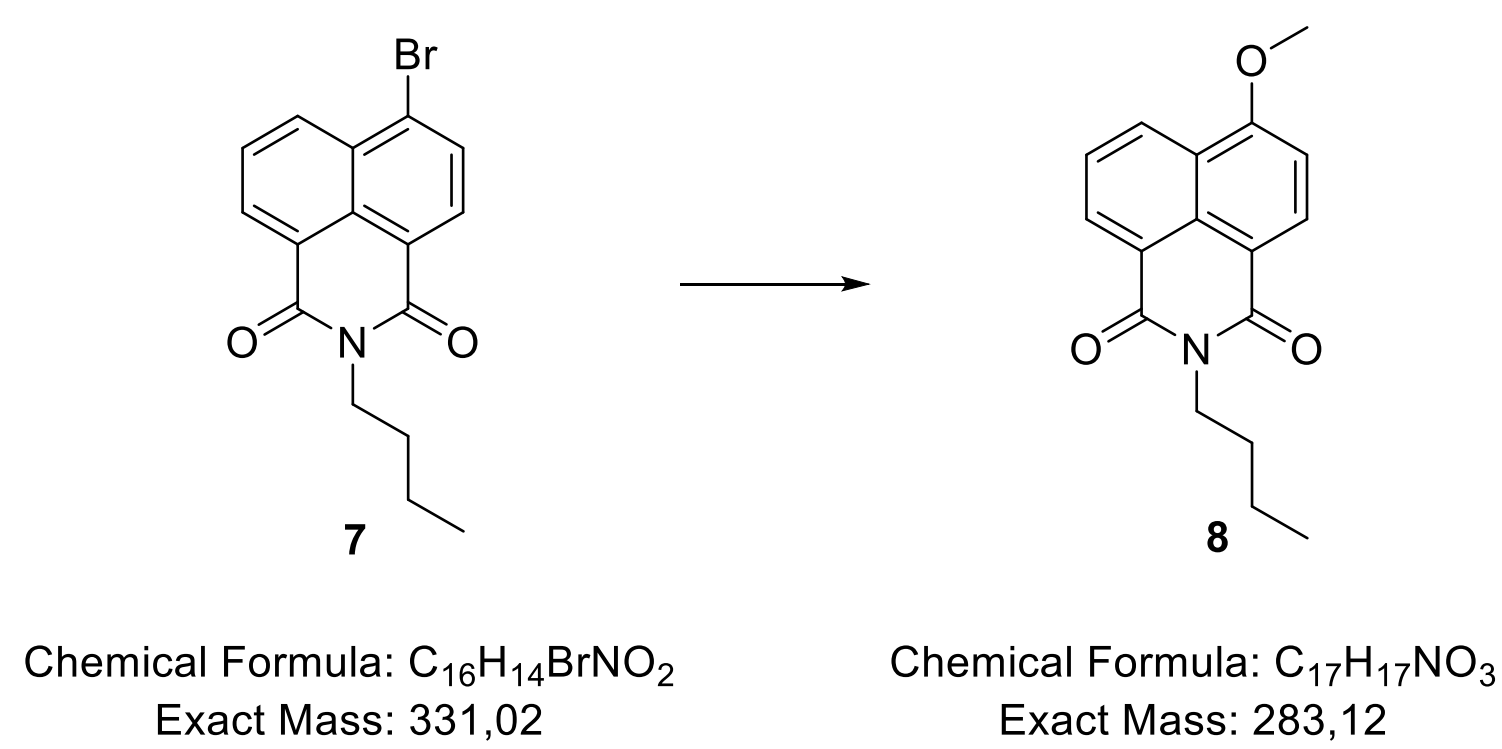


**Scheme S4. Synthesis compound 8.**

300 mg (1.07 mmol) of compound **8** were added in 10 mL of 57% hydroiodic acid, the reaction was heated to reflux at 140°C for 3 hours. After the time had elapsed, the reaction was cooled to room temperature and filtered. The compound **9** was dried under vacuum leading to as a light-yellow needle (260 mg, 90%). $^{1}$H NMR (400 MHz, DMSO) δ 11.85 (s, 1H), 8.57 – 8.51 (m, 1H), 8.50 – 8.45 (m, 1H), 8.36 (d, *J* = 8.2 Hz, 1H), 7.82 – 7.74 (m, 1H), 7.16 (d, *J* = 8.2 Hz, 1H), 4.07 – 3.95 (m, 2H), 1.67 – 1.54 (m, 2H), 1.43 – 1.26 (m, 2H), 0.92 (t, *J* = 7.4 Hz, 3H). UHPLC MS/MS: $[M+H]^+$:264.16.

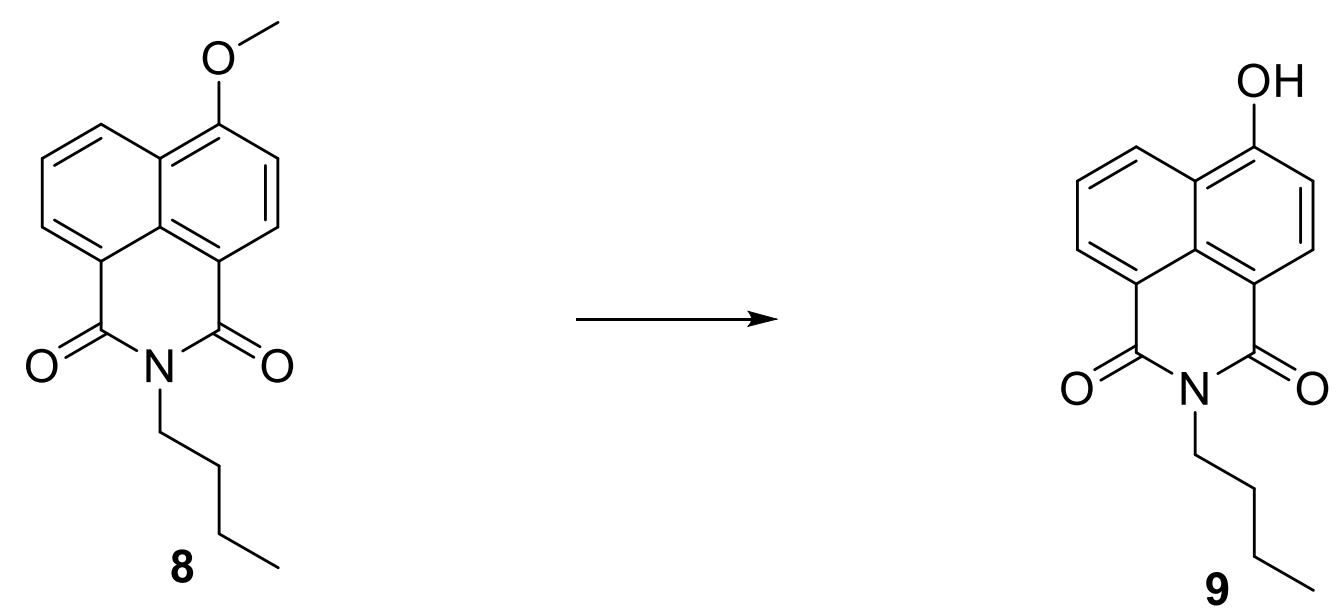


Chemical Formula: $C_{17}H_{17}NO_3$
Exact Mass: 283,12

Chemical Formula: $C_{16}H_{15}NO_3$
Exact Mass: 269,11

**Scheme S5. Synthesis compound 9.**

100 mg (0.371 mmol) of compound **9** and 100 mg (0.714 mmol) HMTA were added in 10 mL of TFA, the reaction was heated at reflux for 12 hours. After time, the reaction was cooled to room temperature. The reaction mixture was diluted with distilled water and the precipitates formed were filtered and washed with plenty of water. The compound was dried under vacuum leading to **2a** (**NAF**) as a light-yellow (99.2 mg, 90%). $^{1}$H NMR (400 MHz, DMSO) δ 10.37 (d, *J* = 5.5 Hz, 1H), 8.72 (s, 2H), 8.58 (s, 1H), 7.87 (d, *J* = 7.2 Hz, 1H), 4.03 (d, *J* = 6.2 Hz, 3H), 1.60 (d, *J* = 6.5 Hz, 2H), 1.43 – 1.32 (m, 2H), 0.92 (d, *J* = 6.7 Hz, 4H). UHPLC MS/MS: $[M+H]^+$:298.09.

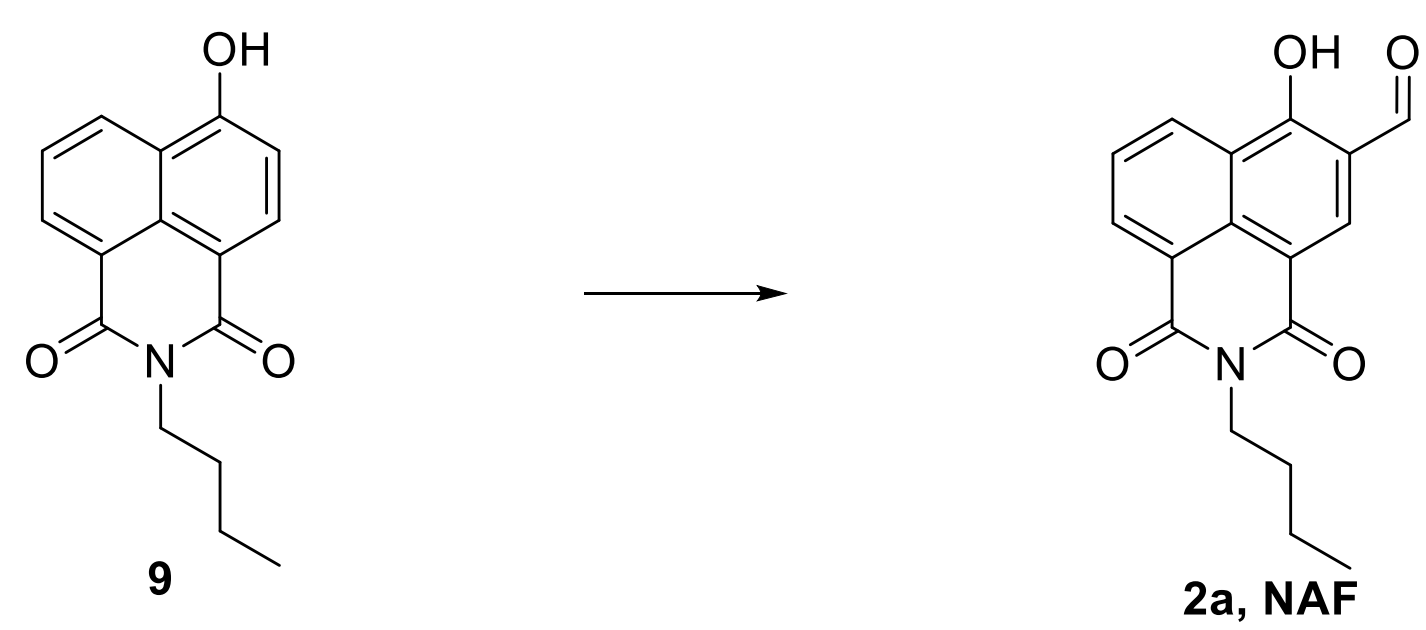


Chemical Formula: $C_{16}H_{15}NO_3$
Exact Mass: 269,11

Chemical Formula: $C_{17}H_{15}NO_4$
Exact Mass: 297,10

**Scheme S6. Synthesis compound 2a (NAF).**

The *N*-butylated naphthalimide precursor **2a** (NAF, 98,13 mg, 0.30 mmol) and the coumarin **3** (43 mg, 0.2 mmol) were dissolved in ethanol (3 mL), and the solution was stirred at 80 °C for 6 h. [31,54] After the reaction time, the solution was added to water, the solid formed was recovered by filtration and washed thoroughly with ethanol, and the solid was dried under vacuum for 24 h. **1a** (Scheme S7) was obtained as a purple solid (yield: 80%) with a melting point of 180-190°C. $^1$H-NMR (400 MHz, $CDCl_3$) δ 15.81 (d, *J* = 9.3 Hz, 1H), 9.16 (d, J = 9.5 Hz, 1H), 8.71 (dd, *J* = 8.0, 1.3 Hz, 1H), 8.58 (d, *J* = 7.5, 1.3 Hz, 1H), 8.40 (s, 1H), 7.64 (t, *J* = 7.8 Hz, 1H), 7.56 (s, 1H), 6.91 (s, 1H), 4.19 – 4.12 (m, 2H), 3.33 (m, 4H), 2.90 (t, *J* = 6.2 Hz, 2H), 2.79 (t, *J* = 6.2 Hz, 2H), 2.01 (m, 4H), 1.70 (m, 2H), 1.47 – 1.41 (m, 2H), 0.98 (t, *J* = 7.4 Hz, 3H). $^{13}$C NMR (100.13 MHz, $CDCl_3$) δ 177.62, 164.40, 163.60, 157.80, 156.09, 149.94, 146.80, 137.72, 133.15, 131.94, 131.19, 131.02, 127.46, 125.68, 125.35, 122.58, 119.88, 117.51, 111.70, 110.72, 107.42, 106.48, 50.17, 49.75, 40.08, 30.32, 27.51, 21.18, 20.44, 20.26, 20.23, 13.89. UHPLC-MS/MS $[M-H]^+$: m/z 536.33.

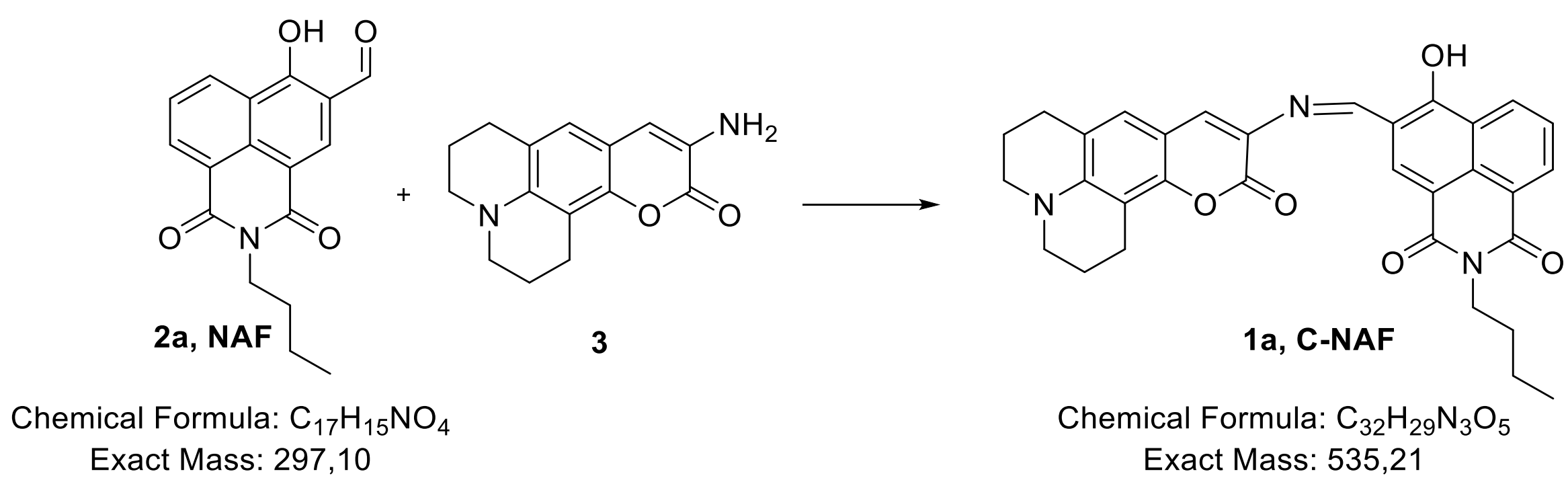


**Scheme S7. Synthesis of 1a (C-NAF).**

### S1.3 Naphthalimide-Morpholine (NAF-M)

4-bromo-1,8-naphthalic anhydride (**6**, 9.93 g, 36.00 mmol) and 4-(2-aminoethyl)-morpholine (10 g, 0.072 mol) were dissolved in absolute ethanol (200 mL), the solution was refluxed for 24 hours. After cooling to room temperature, the yellowish precipitate was collected by filtration and dried under vacuum to yield (**10**, 12.5 g, 90%) as a yellow solid. $^1$H-NMR (400 MHz, DMSO) δ 8.57 (d, 1H), 8.54 (d, 1H), 8.34 (d, 1H), 8.22 (d, 1H), 8.00 (t, 1H), 4.17 (t, 2H), 3.53 (t, 4H), 2.57 (t, 2H), 2.50 – 2.43 (m, 4H). UHPLC MS/MS: $[M+H]^+$: 389.0495.

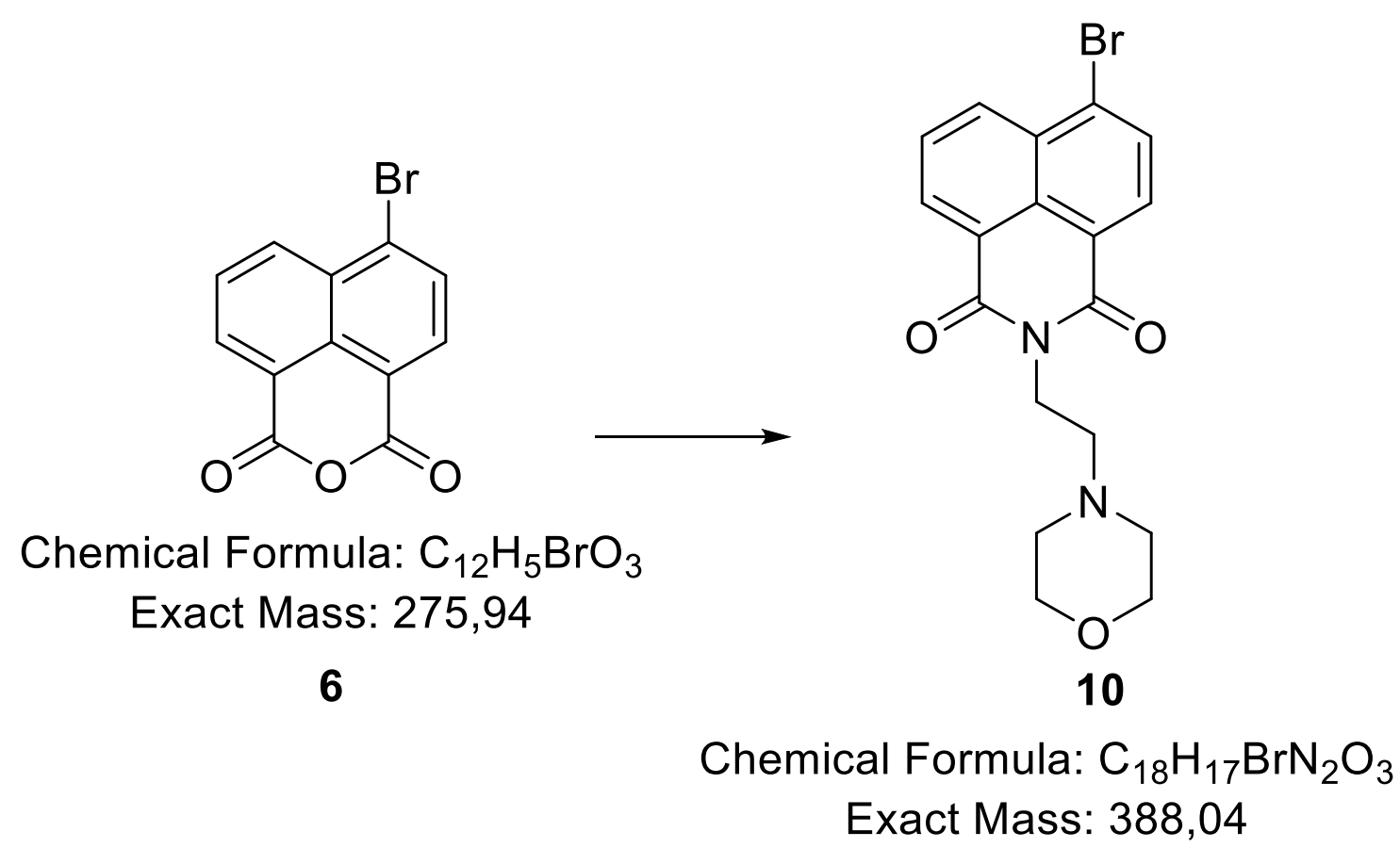


**Scheme S8. Synthesis of compound 10.**

Compound **10** (1.05 g, 2.70 mmol), NHS (350 mg, 3.04 mmol, 1.1 equiv.) and $K_2CO_3$ (1.24 g, 8.96 mmol, 3.3 equiv.) were dissolved in DMSO (12 mL) and the resultant mixture heated at 80 °C for 135 minutes. After this time the reaction mixture was cooled to room temperature, then diluted with $H_2O$ (100 mL), and acidified to pH = 6 using 0.5 M HCl. The aqueous mixture was then extracted with 10 % MeOH in $CH_2Cl_2$ (3 × 50 mL), brine (3 x 50 mL) and dried with $MgSO_4$. The compound **11** was dried under vacuum leading to as a light-yellow (730 mg, 83 %). $^1$H-RMN: $^1$H NMR (400 MHz, DMSO) δ 9.33 (s, 1H), 8.60 (d, 1H), 8.53 (d, 1H), 8.41 (d ,1H), 7.82 (t, 1H), 7.20 (d, 1H), 4.40 (t, 2H), 4.10 – 3.99 (m, 2H), 3.69 (d, 2H), 3.64 – 3.47 (m, 4H). UHPLC MS/MS: $[M+H]^+$: 327.18.

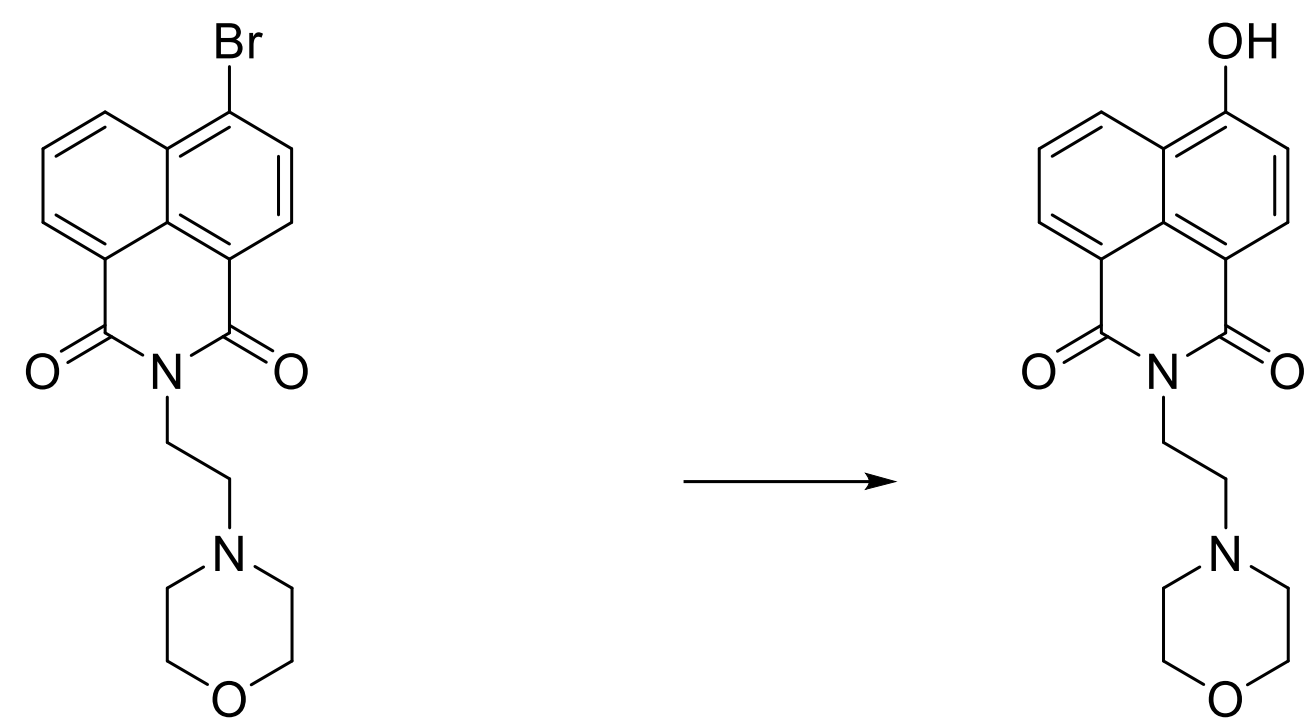


**10**
Chemical Formula: $C_{18}H_{17}BrN_2O_3$
Exact Mass: 388,04

**11**
Chemical Formula: $C_{18}H_{18}N_2O_4$
Exact Mass: 326,13

**Scheme S9. Synthesis compound 11.**

Compound **11** (0.50 g, 1.53 mmol) and HMTA (0.42 g, 3.0 mmol) were dissolved in trifluoroacetic acid (15 mL). The solution was refluxed for 24 hours, then the solution was cooled to room temperature. The reaction mixture was diluted with 30 mL distilled water, and the precipitated formed were filtered and washed with distilled water (x5). The compound was dried under vacuum leading to **2b** (**NAF-M**) as a yellow solid (487 mg, 90 %). $^{1}$H-NMR (400 MHz, DMSO) δ 10.32 (s, 1H), 8.69 (m, 1H), 8.63 – 8.55 (m, 1H), 8.51 (m, 1H), 8.45 (m, 1H), 7.80 – 7.72 (m, 1H), 4.43 – 4.34 (m, 4H), 3.67 (s, 2H), 3.52 (t, 4H), 3.39 (d, 2H). UHPLC MS/MS: $[M\text{-}H]^{+}$: 355.22.

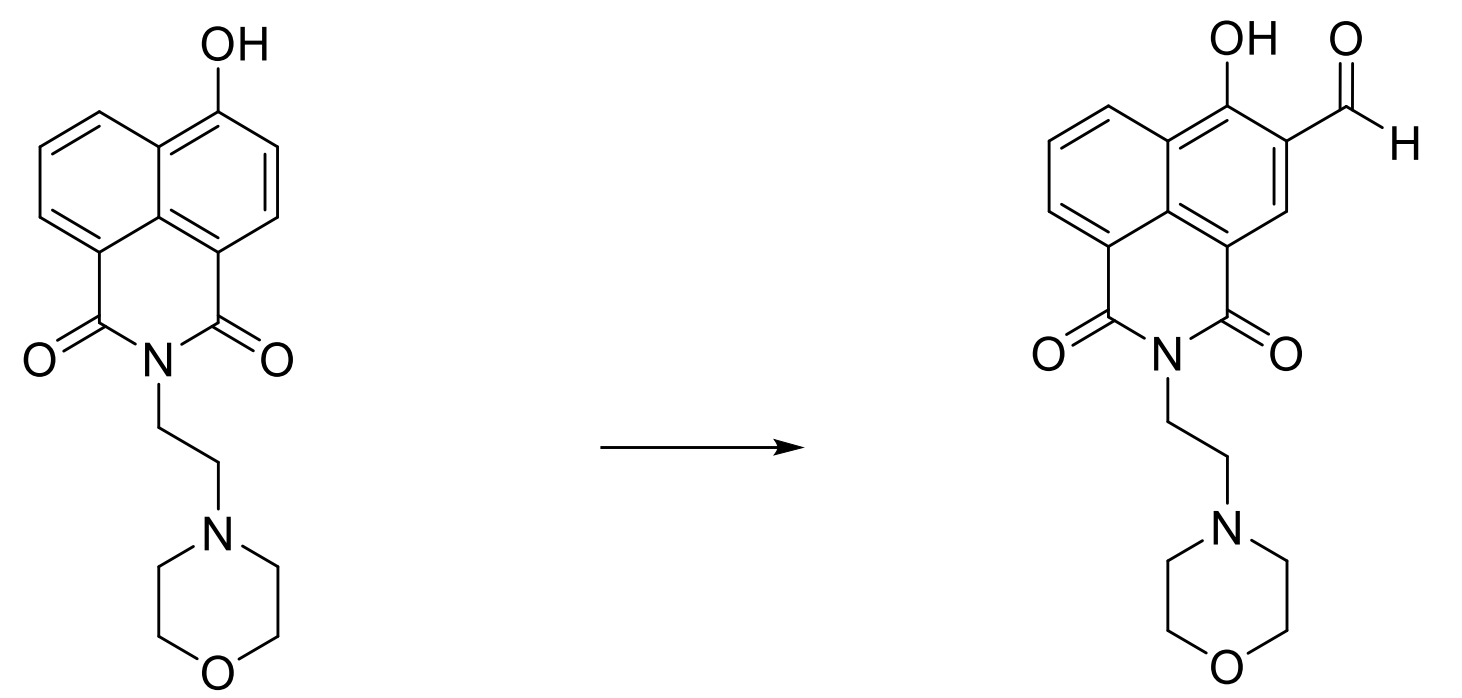


**11**
Chemical Formula: $C_{18}H_{18}N_2O_4$
Exact Mass: 326,13

**2b, NAF-M**
Chemical Formula: $C_{19}H_{18}N_2O_5$
Exact Mass: 354,12

**Scheme S10. Synthesis of compound 2b (NAF-M).**

The N-morpholinyl naphthalimide precursor 2b (NAF-M, 100 mg, 0.168 mmol) and the coumarin 3 (30.72 mg, 0.12 mmol) were dissolved in ethanol (15 mL), and the solution was refluxed for 3 h. 31,54 The excess solvent was removed under reduced pressure, and the product was purified with a silica gel chromatography column, with dichloromethane 90% and 10% methanol. The solid was dried under vacuum for 24 h. 1b (Scheme S11) was obtained as a purple solid (yield: 60%) with a melting point of 200-210 °C. $^{1}$H-NMR: 1H-NMR (400 MHz, CDCl3) δ 7.98 (d,1H), 7.83 (s, 1H), 7.65 (d,1H), 7.56 (s, 1H), 7.31 (t,1H), 7.05 (s, 1H), 6.90 (s, 1H), 5.30 (s, 1H), 4.34 (d, 4H), 3.47 (d, 2H), 3.33 (m, 4H), 2.89 (t, 4H), 1.90 (s, 2H), 1.22 – 1.08 (m, 4H), 0.88 (t, 4H). $^{13}$C NMR (100.13 MHz, $CDCl_3$) δ (ppm): 166.29, 165.47, 164.33, 163.86, 163.59, 139.88, 135.28, 133.90, 131.51, 129.72, 128.27, 127.10, 126.00, 125.48, 123.06, 122.28, 119.75, 115.22, 112.27, 109.80, 63.93, 57.48, 55.62, 53.42, 52.47, 37.38, 33.78, 29.71. UHPLC MS/MS:[M+H]+: 593.39.

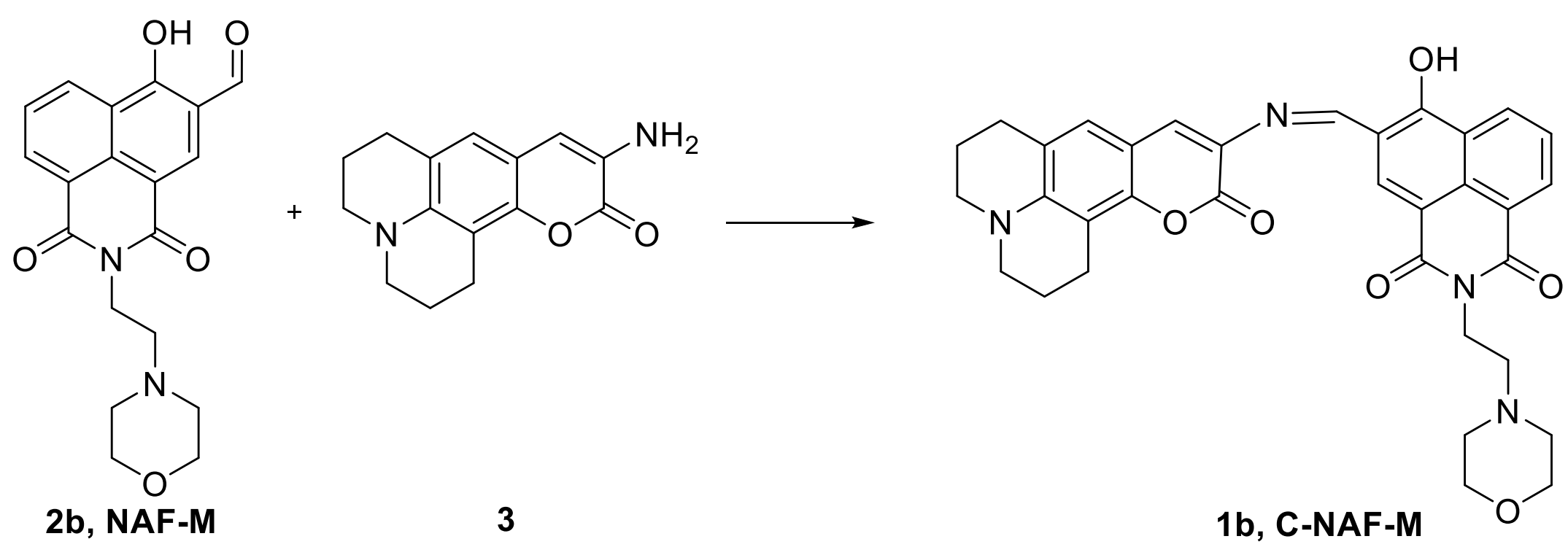


**2b, NAF-M**

Chemical Formula: $C_{19}H_{18}N_2O_5$
Exact Mass: 354,12

**3**

**1b, C-NAF-M**

Chemical Formula: $C_{34}H_{32}N_4O_6$
Exact Mass: 592,23

**Scheme S11. Synthesis of compound 1b (C-NAF-M).**

### S1.4 Naphthalimide-Biotin (NAF-B7)

4-bromo-1,8-naphthalic anhydride (**6**, 1 g, 3.609 mmol) and *N*-Boc-ethylenediamine (1.143 mL, 7.218 mmol) were dissolved in absolute ethanol (50 mL), and the solution was refluxed for 24 hours. After cooling room temperature, the solid was filtered. The compound **12** was dried under vacuum leading to a light yellow solid (1.2 g, 80 %). $^{1}$H-NMR (400 MHz, $CDCl_3$) δ 8.69 – 8.63 (m, 1H), 8.57 (dd, *J* = 8.5, 1.2 Hz, 1H), 8.45 – 8.38 (m, 1H), 8.08 – 8.00 (m, 1H), 7.92 – 7.78 (m, 1H), 4.92 (s, 1H), 4.35 (t, *J* = 5.8 Hz, 2H), 3.58 – 3.46 (m, 2H), 1.27 (s, 9H). UHPLC MS/MS: $[M+H]^+$: 419.

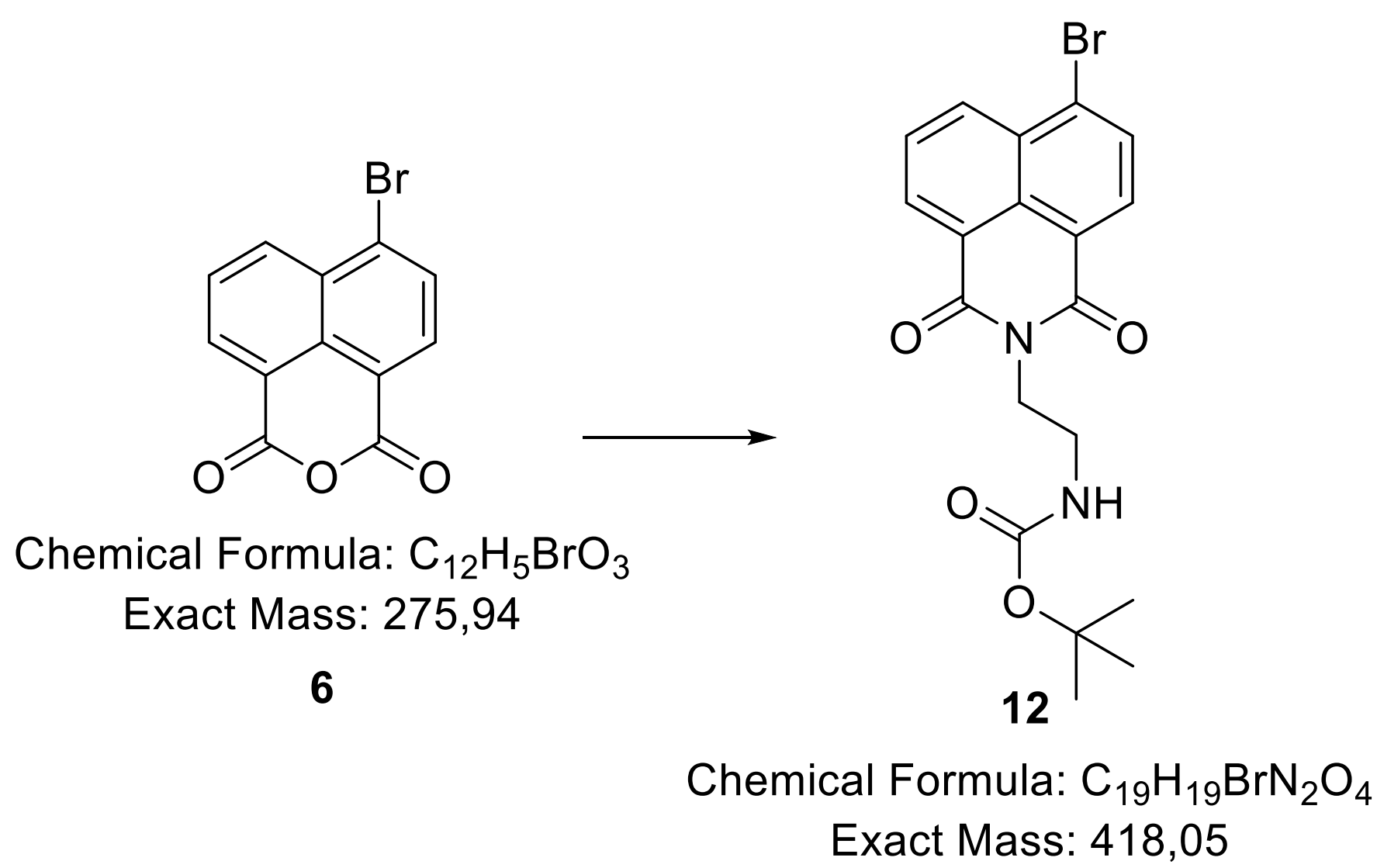


**Scheme S12. Synthesis compound 12.**

TFA 1.1 mL (14.31 mmol) was added dropwise to a solution of compound **12** (600 mg, 1.43 mmol) in $CH_2Cl_2$ (25 mL) at 0 °C. The mixture was stirred at room temperature for 6 hours. The compound was dried under vacuum leading to **13** as a light-yellow solid (409.2 mg, 90%). $^1$H-NMR (400 MHz, DMSO) δ 8.62 (q, *J* = 1.1 Hz, 1H), 8.61 – 8.57 (m, 1H), 8.38 (d, *J* = 7.9 Hz, 1H), 8.27 (d, *J* = 7.8 Hz, 1H), 8.04 (dd, *J* = 8.5, 7.3 Hz, 1H), 7.83 (s, 2H), 4.31 (t, *J* = 5.9 Hz, 2H), 3.16 (q, *J* = 5.8 Hz, 2H). UHPLC MS/MS: $[M+H]^+$: 319.01

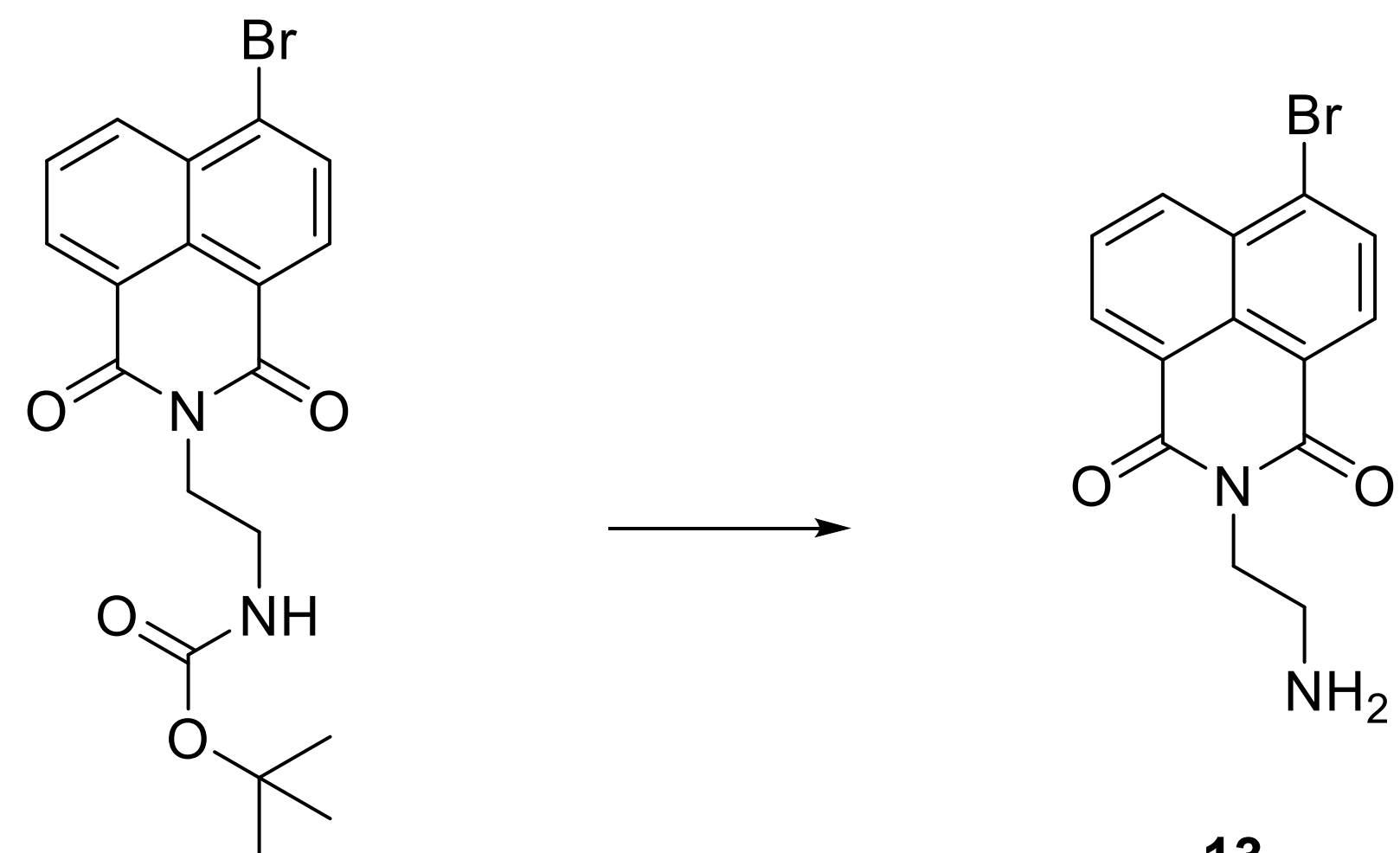


**12**

Chemical Formula: $C_{19}H_{19}BrN_2O_4$
Exact Mass: 418,05

**13**

Chemical Formula: $C_{14}H_{11}BrN_2O_2$
Exact Mass: 318,00

**Scheme S13. Synthesis compound 13.**

Biotin (812.6 mg, 3.326 mmol) and HATU (993.68 mg, 2.615 mmol) were dissolved in 50 mL of dichloromethane. Subsequently, DIPEA (1.58 mL, 9.26 mmol) was added. The solution was stirred at room temperature for 20 minutes, then compound **13** (720.5 mg, 2.257 mmol) was added to the reaction mixture. Thus, the reaction mixture was stirred for 24 hours at room temperature. Finally, the solvent was removed under vacuum and the solid was washed with acid water (0.5 M HCl) several times leading to **14** as a yellow solid (982 mg, 80 %). $^1$H NMR (400 MHz, DMSO) 8.62 – 8.50 (m, 2H), 8.35 (d, 1H), 8.24 (d, 1H), 8.02 (t, 1H), 7.87 (t, 1H), 6.36 (d, 2H), 4.33 – 4.26 (m, 1H), 4.09 – 4.01 (m, 1H), 3.40 (q, 2H), 2.57 (d, 2H), 2.20 (t, 1H), 1.92 (t, 2H), 1.59 – 1.46 (m, 2H), 1.41 – 1.30 (m, 4H), 1.29 – 1.13 (m, 2H). UHPLC MS/MS: $[M+H]^+$: 545.33

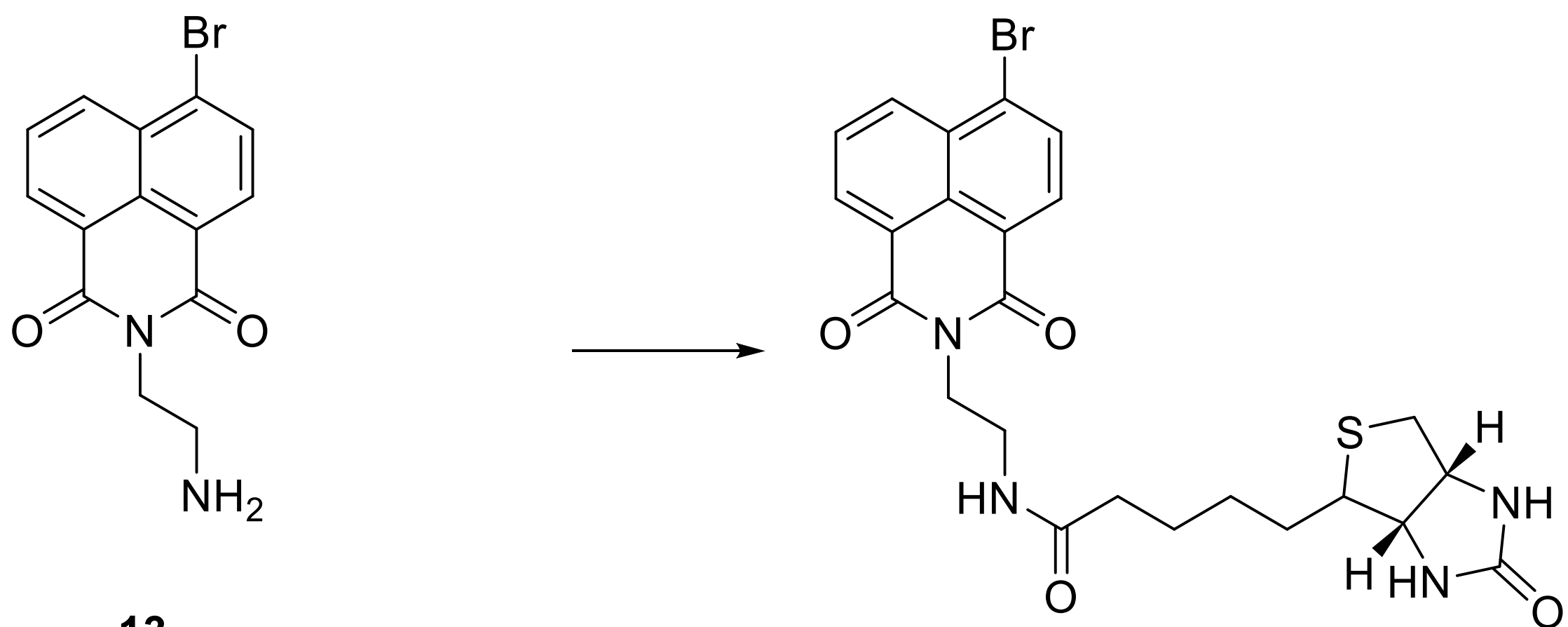


**13**

Chemical Formula: $C_{14}H_{11}BrN_2O_2$
Exact Mass: 318,00

**14**

Chemical Formula: $C_{24}H_{25}BrN_4O_4S$
Exact Mass: 544,08

**Scheme S14. Synthesis of compound 14.**

A 25 mL round bottom flask was charged with compound **14** (987mg, 1.81 mmol), NHS (271 mg, 2.36 mmol) and $K_2CO_3$ (960 mg, 6.94 mmol) in DMSO (12 mL). The mixture was heated at 80 °C for 135 minutes. Then, the reaction mixture was cooled to ambient temperature, diluted with distilled water (25 mL), and acidified to pH = 3 using 0,5 M HCl. Finally, the precipitated were filtered and dried vacuum leading to **15** as a yellow solid (523 mg, 60 %). $^{1}$H-NMR (400 MHz, DMSO) δ 11.87 (s, 1H), 8.54 (d, 1H), 8.48 (d, 1H), 8.36 (d, 1H), 7.86 (t, 1H), 7.77 (dd, 1H), 7.16 (d, 1H), 6.36 (d, 2H), 4.40 – 4.22 (m, 1H), 4.11 (t, 2H), 4.08 – 3.98 (m, 1H), 2.57 (d, 2H), 2.54 (d 1H), 1.94 (t, 2H), 1.60 – 1.46 (m, 2H), 1.43 – 1.30 (m, 4H), 1.27 – 1.14 (m, 2H). UHPLC MS/MS: $[M+H]^+$: 483.32.

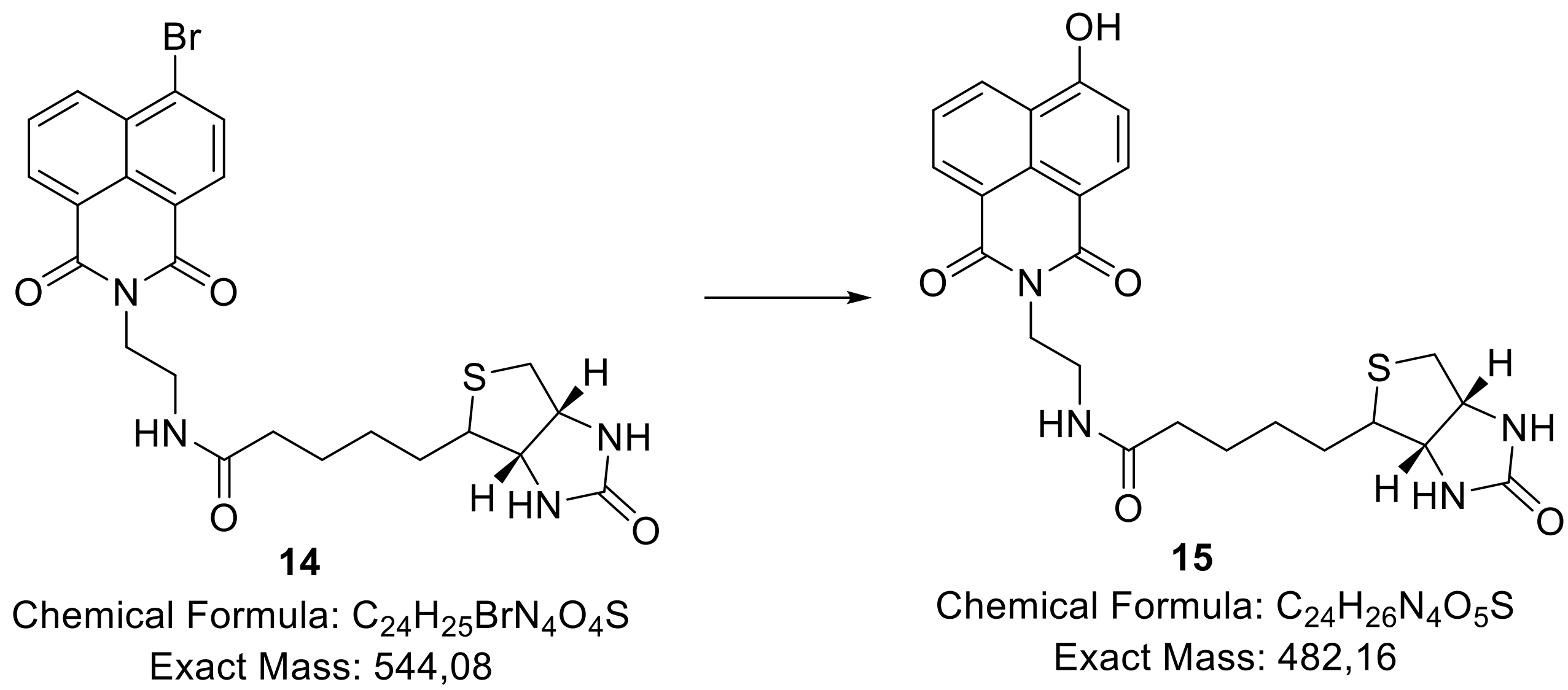


**Scheme S15. Synthesis compound 15.**

Compound **15** (501.6 mg, 1.04 mmol) and HMTA (283.5 mg, 2.02 mmol) were dissolved in trifluoroacetic acid (15 mL). The solution was refluxed for 24 hours. Then, the mixture was cooled to room temperature and diluted with 30 mL distilled water. Finally, the precipitate was filtered and washed with distilled water several times and dried by vacuum leading to **2c** (NAF-B7) as a yellow solid (212 mg, 40 %). $^{1}H$ NMR (400 MHz, DMSO) δ 11.85 (s, 1H), 8.57 – 8.44 (m, 2H), 8.35 (d, 1H), 7.90 – 7.82 (m, 1H), 7.81 – 7.73 (m, 1H), 7.16 (d, 1H), 6.36 (d, 2H), 4.34 – 4.26 (m, 1H), 4.11 (t, 2H), 4.09 – 4.02 (m, 1H), 2.84 – 2.75 (m, 1H), 2.61 – 2.52 (m, 2H), 1.94 (t, 2H), 1.60 – 1.47 (m, 2H), 1.47 – 1.31 (m, 4H), 1.26 – 1.12 (m, 2H). UHPLC MS/MS $[M-H]^-$ : 509.27; $[M-H]^-$ :509.39.

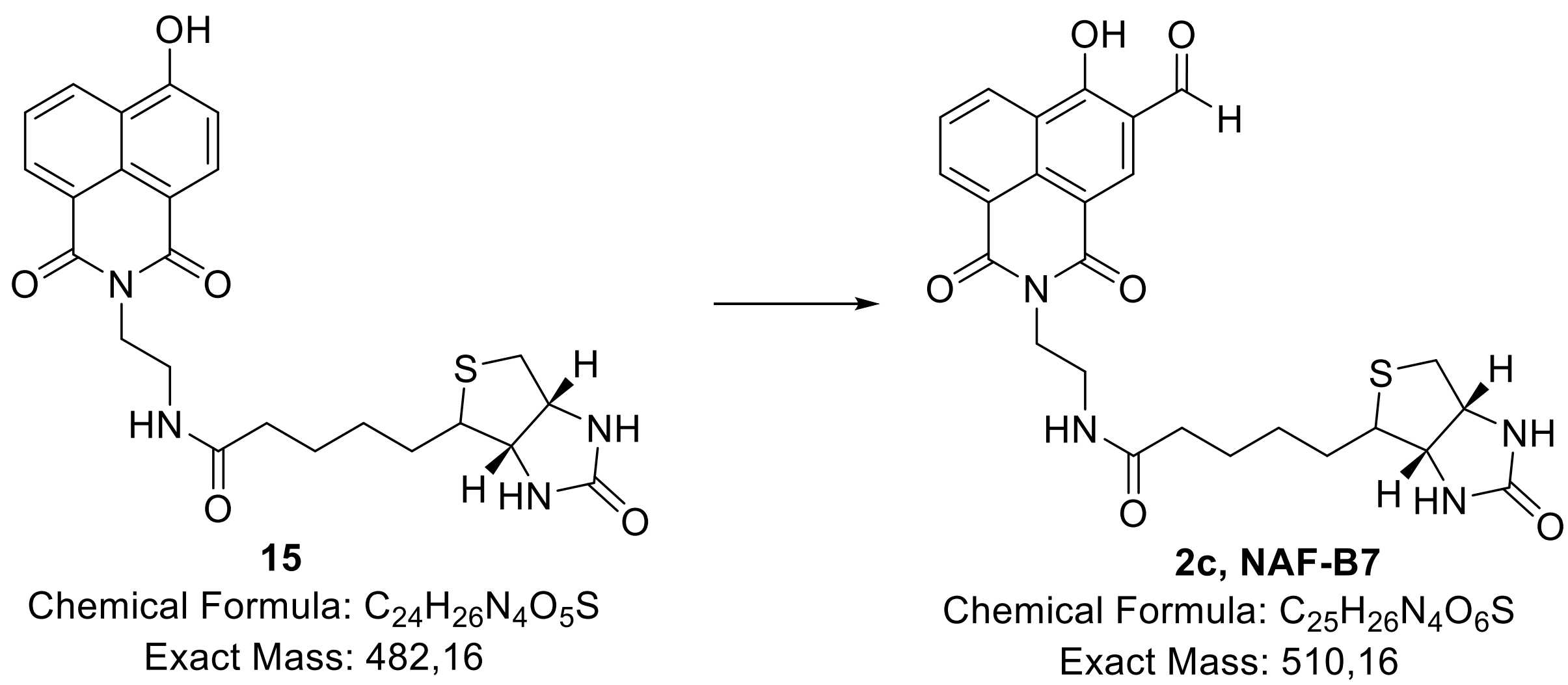


**Scheme S16. Synthesis of compound 2c (NAF-B7).**

The N-biotinyl naphthalimide 2c (NAF-B7, 100 mg, 0.168 mmol) and C-NH2 (30.72 mg, 0,12 mmol) were dissolved in ethanol (15 mL) and was refluxed for 4 h. Then excess solvent was removed under reduced pressure, and the product was purified with a silica chromatography column with CH2Cl2/MeOH, increasing from 5% to 10% methanol. After that, solvent was removed in rotary evaporation, and the solid was dried under vacuum for 24 h. 1c (Scheme S17 and Figure S17). It was obtained as a purple solid (yield: 40%) with a melting point of 220-225°C. $^{1}$H-NMR (400 MHz, DMSO) δ 14.14 (s, 1H), δ 9.15 (s, 1H), 8.61 (s, 1H), 8.39 (m, 2H), 8.19 (s, 1H), 7.91 – 7.66 (m, 2H), 6.76 (s, 1H), 4.29 (m, 2H), 4.12 (m, 4H), 3.15 (m, 7H), 2.85 – 2.60 (m, 6H), 2.00 (s, 2H), 1.90 (m, 4H), 1.45 (m, 4H), 1.26 (m, 2H). UHPLC MS/MS: [M-H]+: 747.46

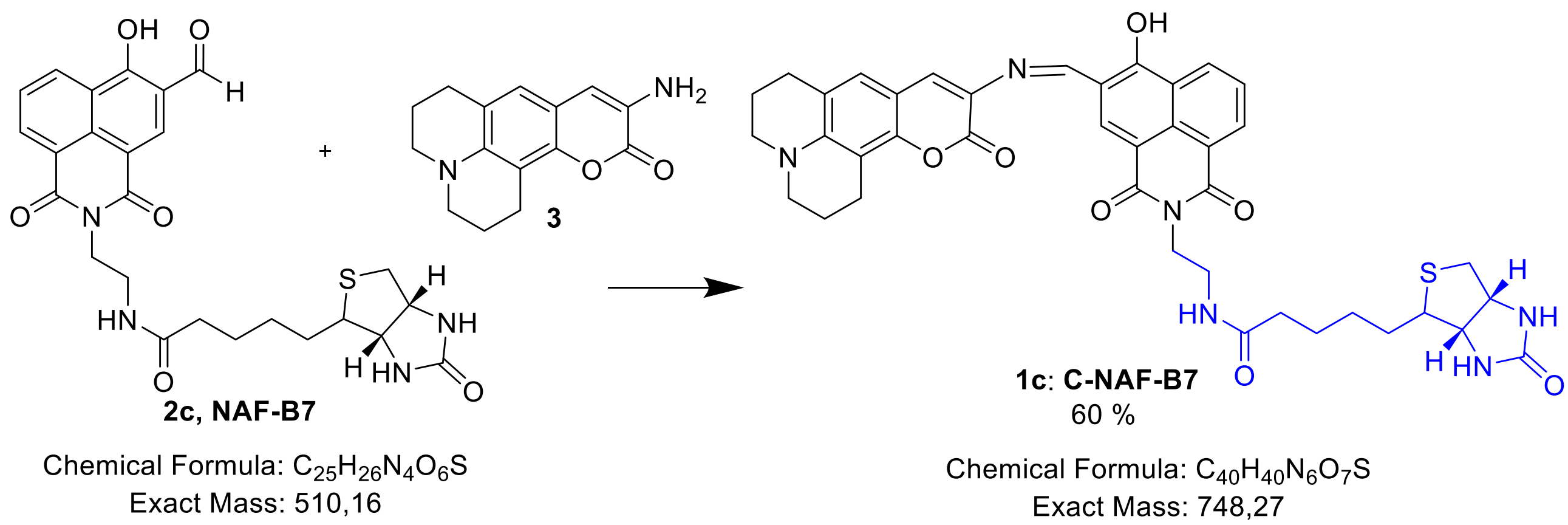


**Scheme S17. Synthesis of compound 1c (C-NAF-B7).**

## S2. NMR and UHPLC-MS/MS characterization

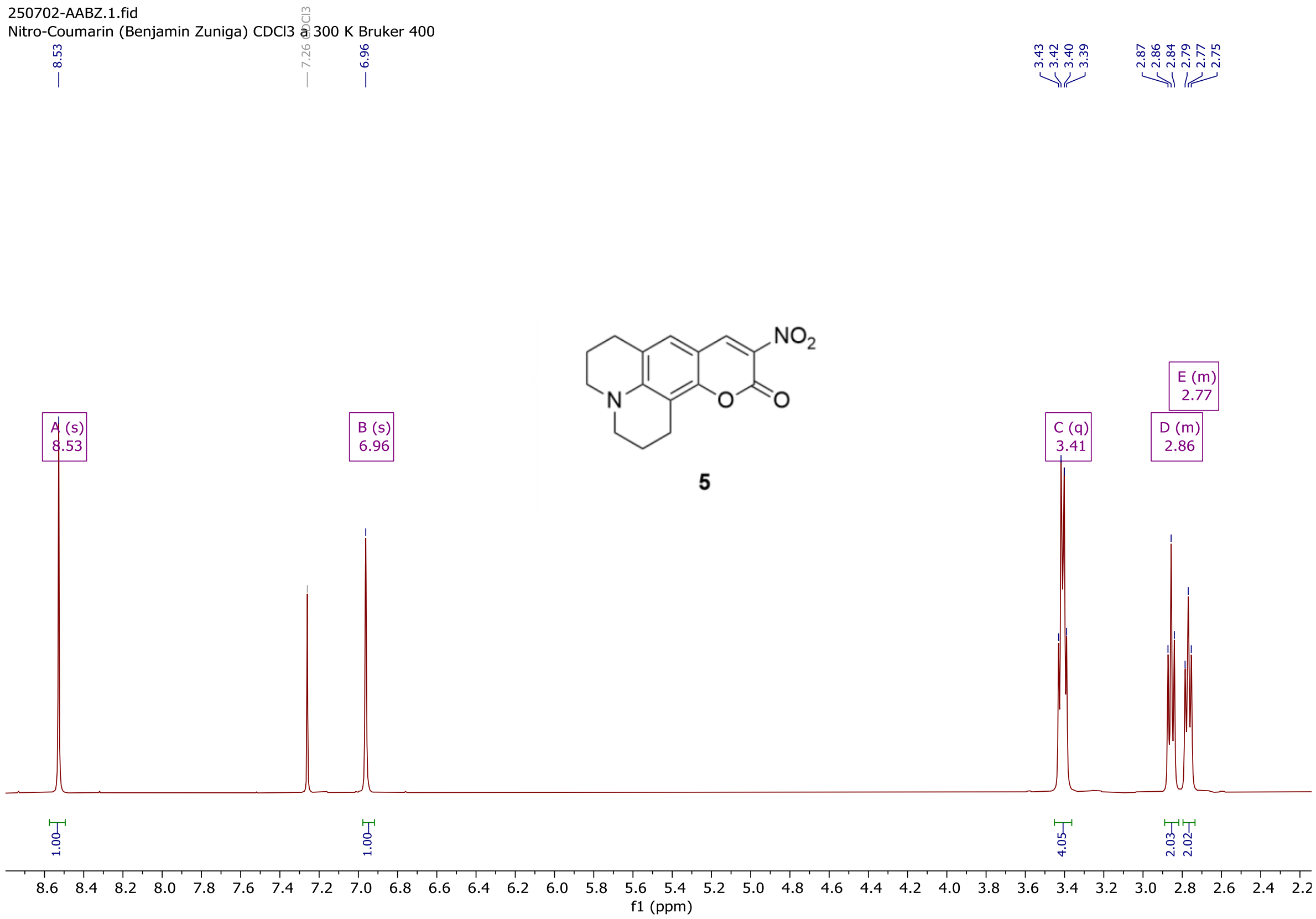


**Figure S1. $^1$H NMR in $CDCl_3$ of compound 5.**

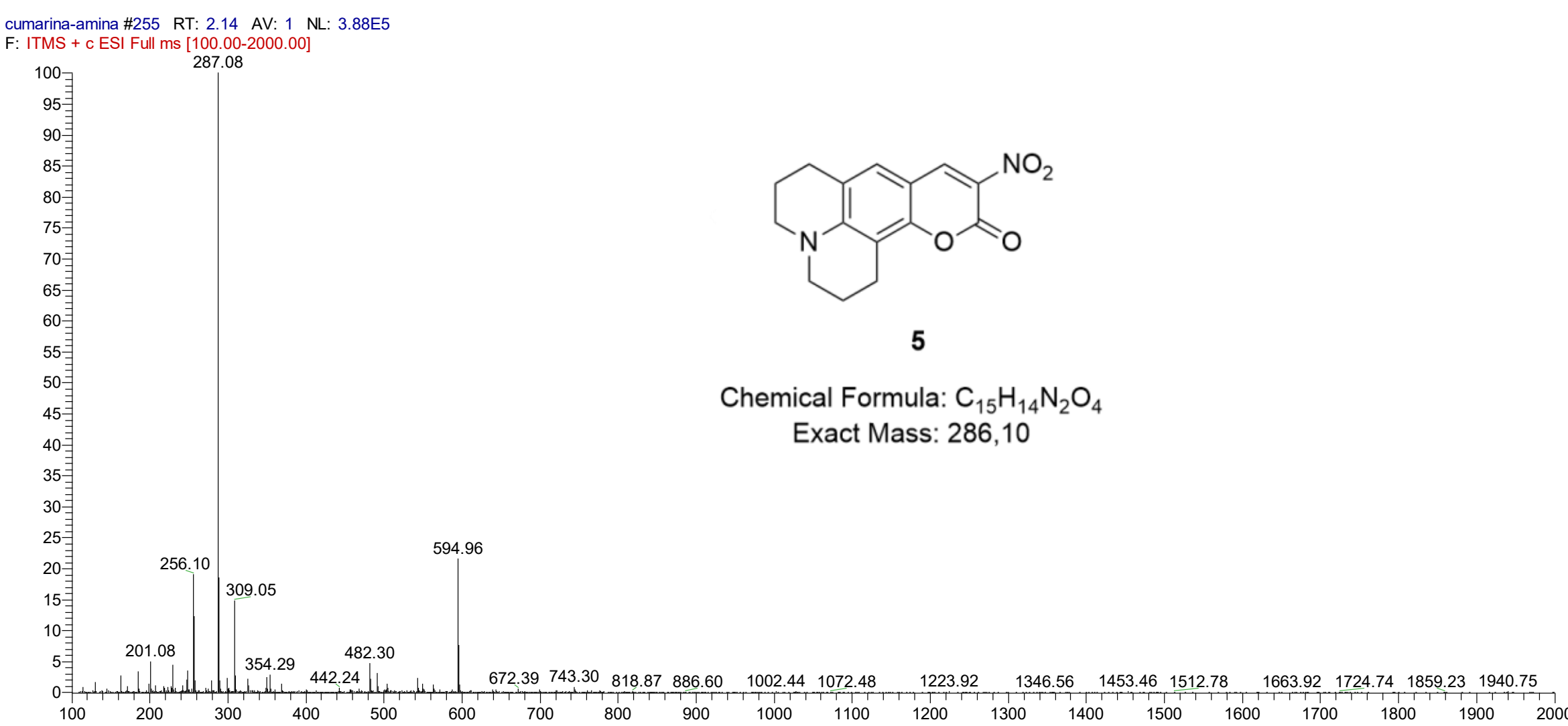


**Figure S2. UHPLC MS/MS in positive mode of compound 5.**

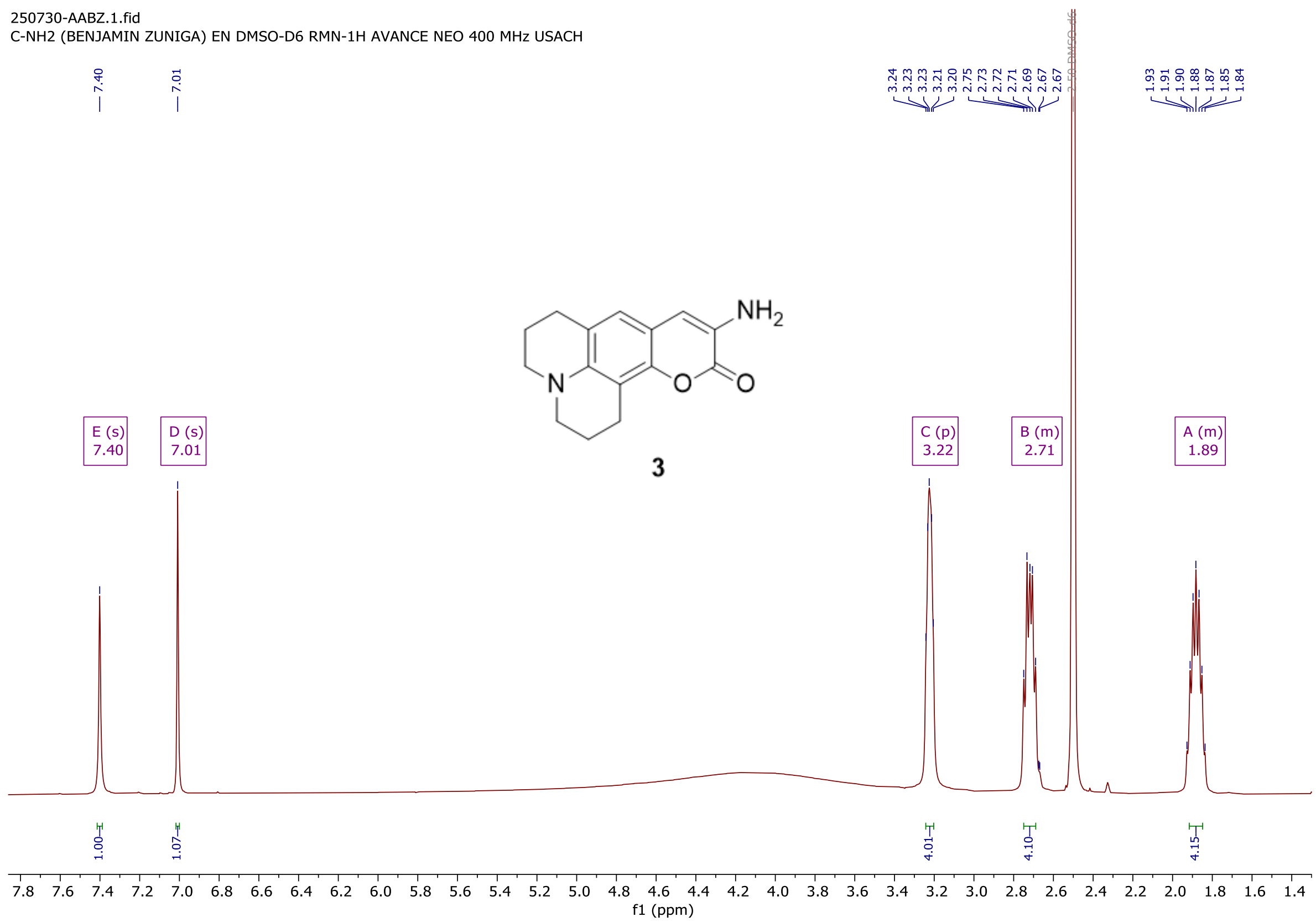


**Figure S3. $^1$H NMR in $CDCl_3$ of compound 3.**

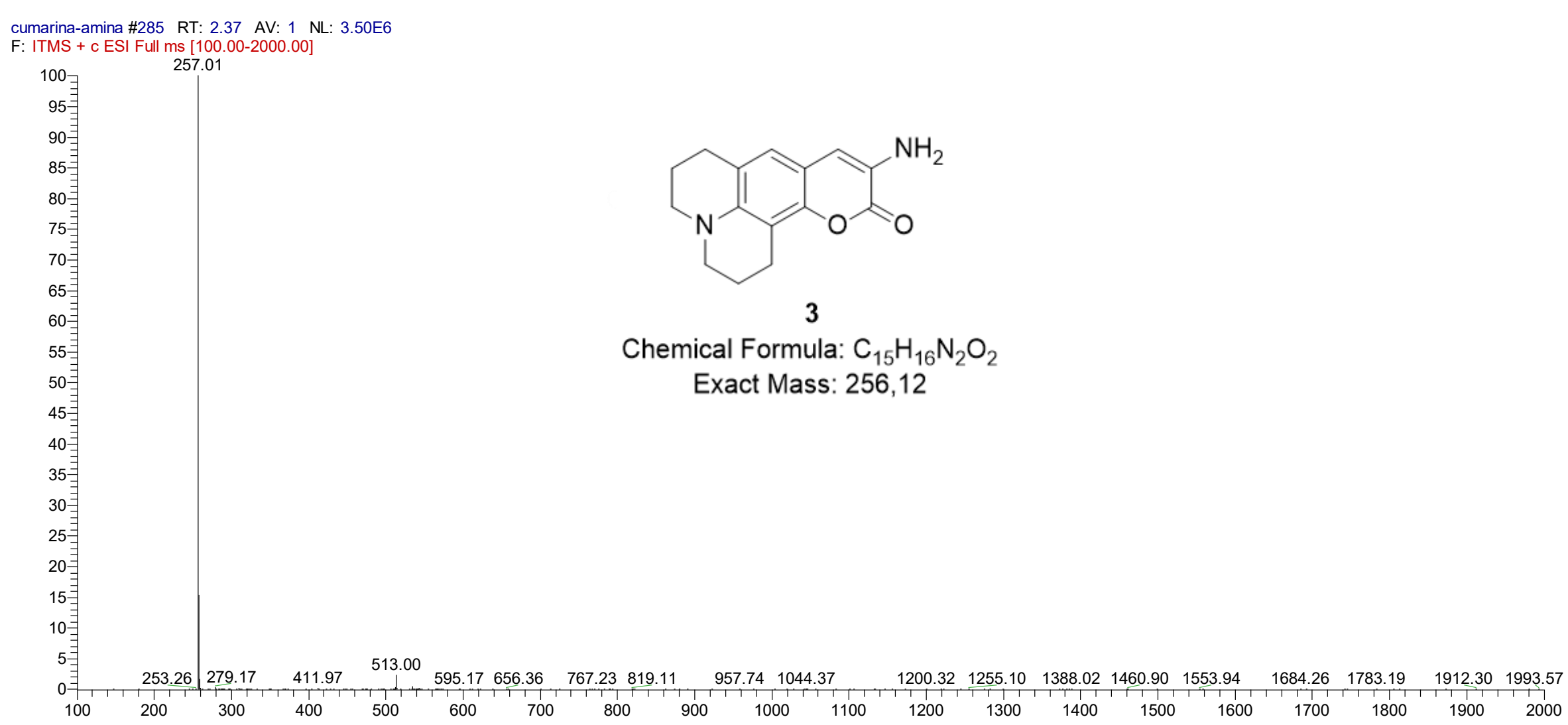


**Figure S4. UHPLC MS/MS in positive mode of compound 3.**

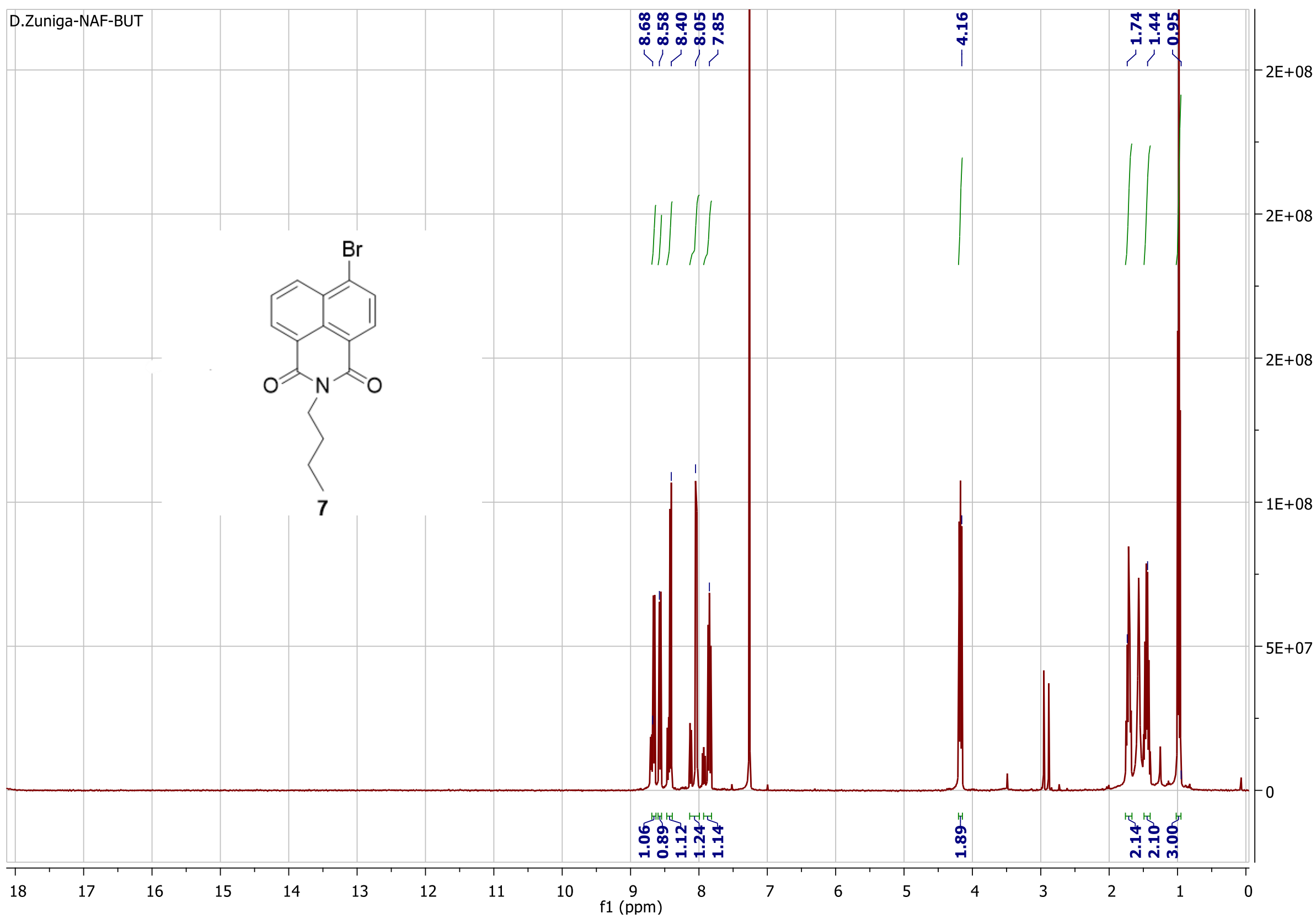


**Figure S5.** $^1$**H NMR in** $CDCl_3$ **of compound 7.**

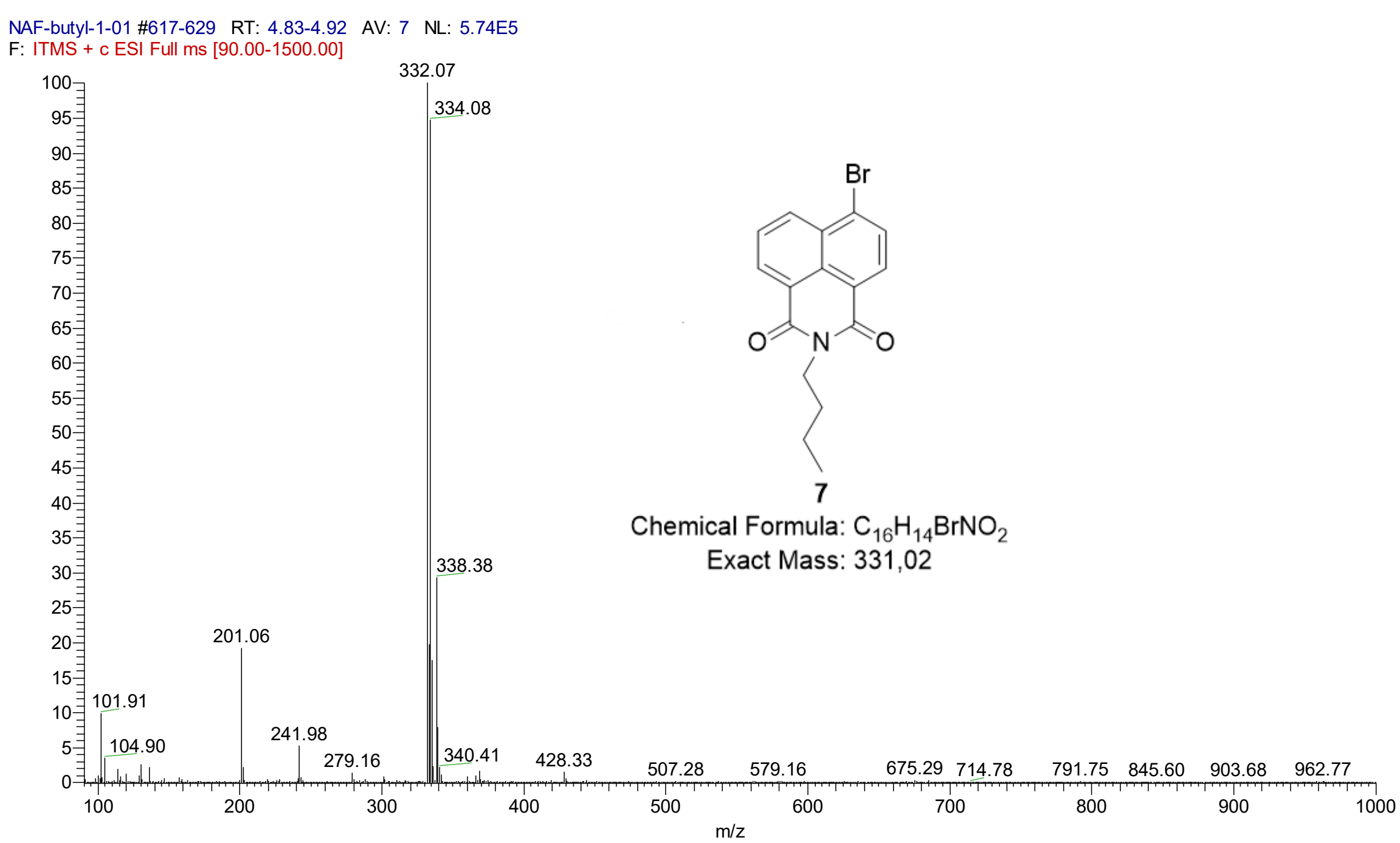


**Figure S6. UHPLC MS/MS in positive mode of compound 7.**

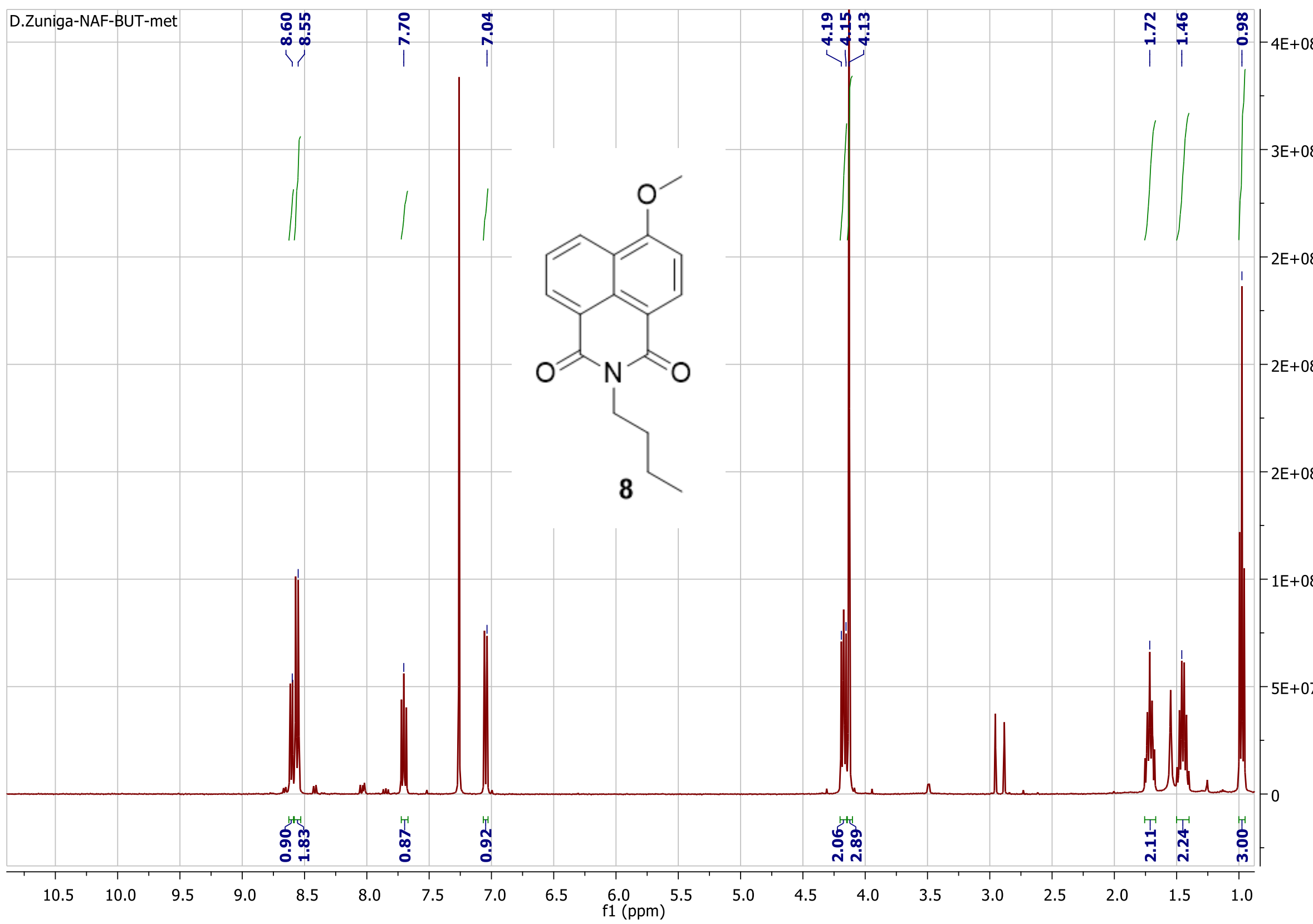


**Figure S7.** **$^1$H NMR in $CDCl_3$ of compound 8.**

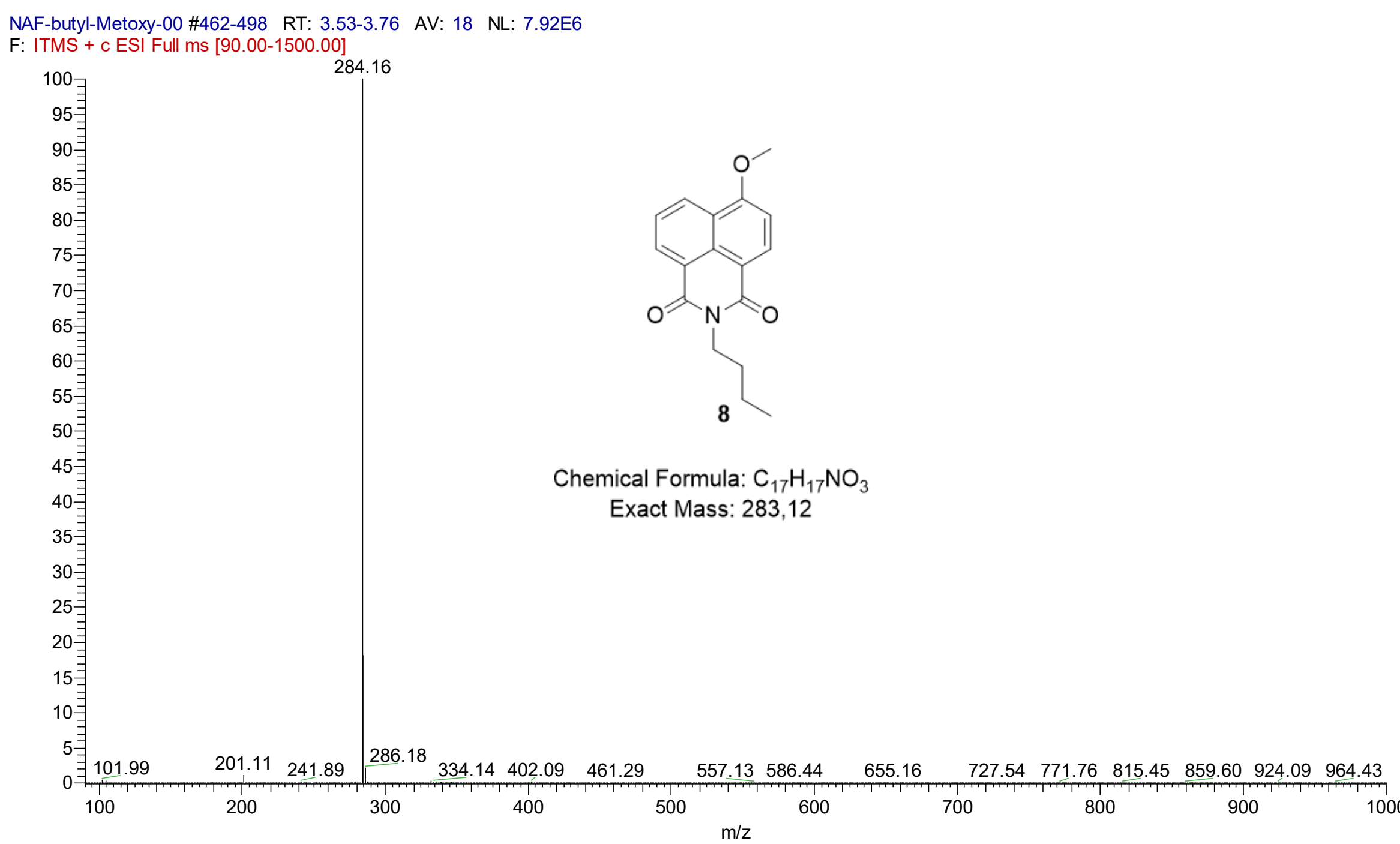


**Figure S8. UHPLC MS/MS in positive mode of compound 8.**

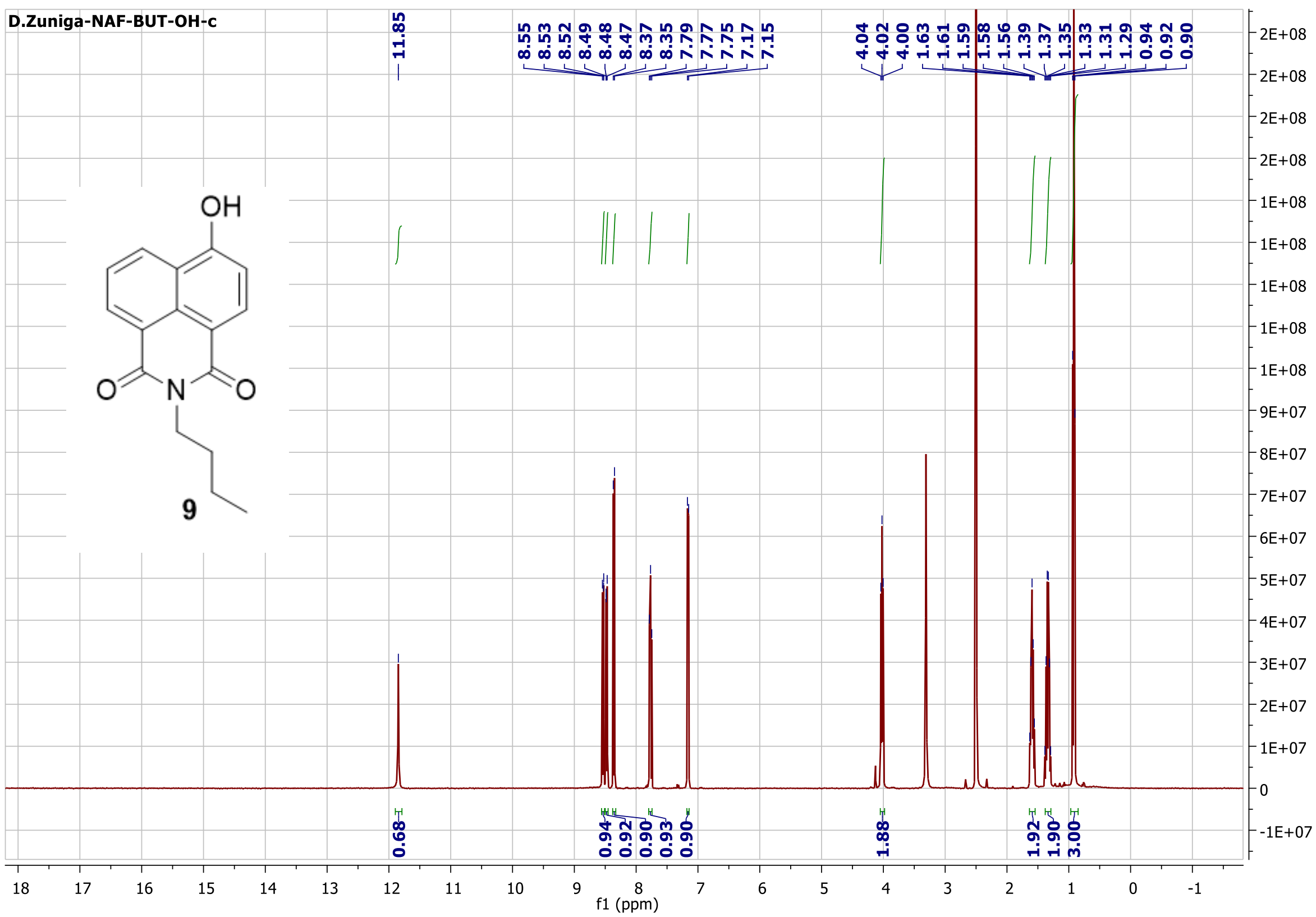


**Figure S9.** $^1H$ **NMR in DMSO-d$_6$ of compound 9.**

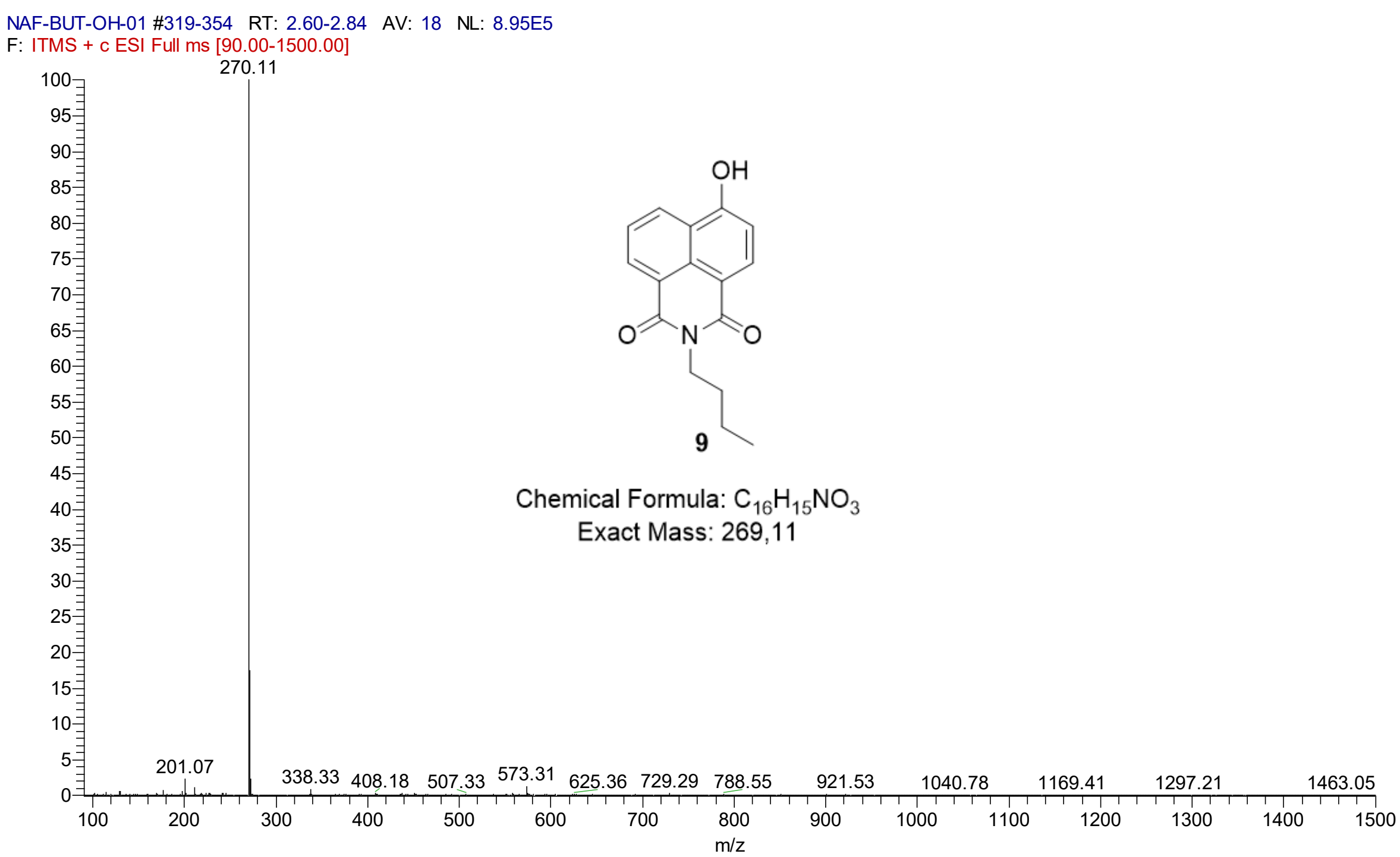


**Figure S10. UHPLC MS/MS in positive mode of compound 9.**

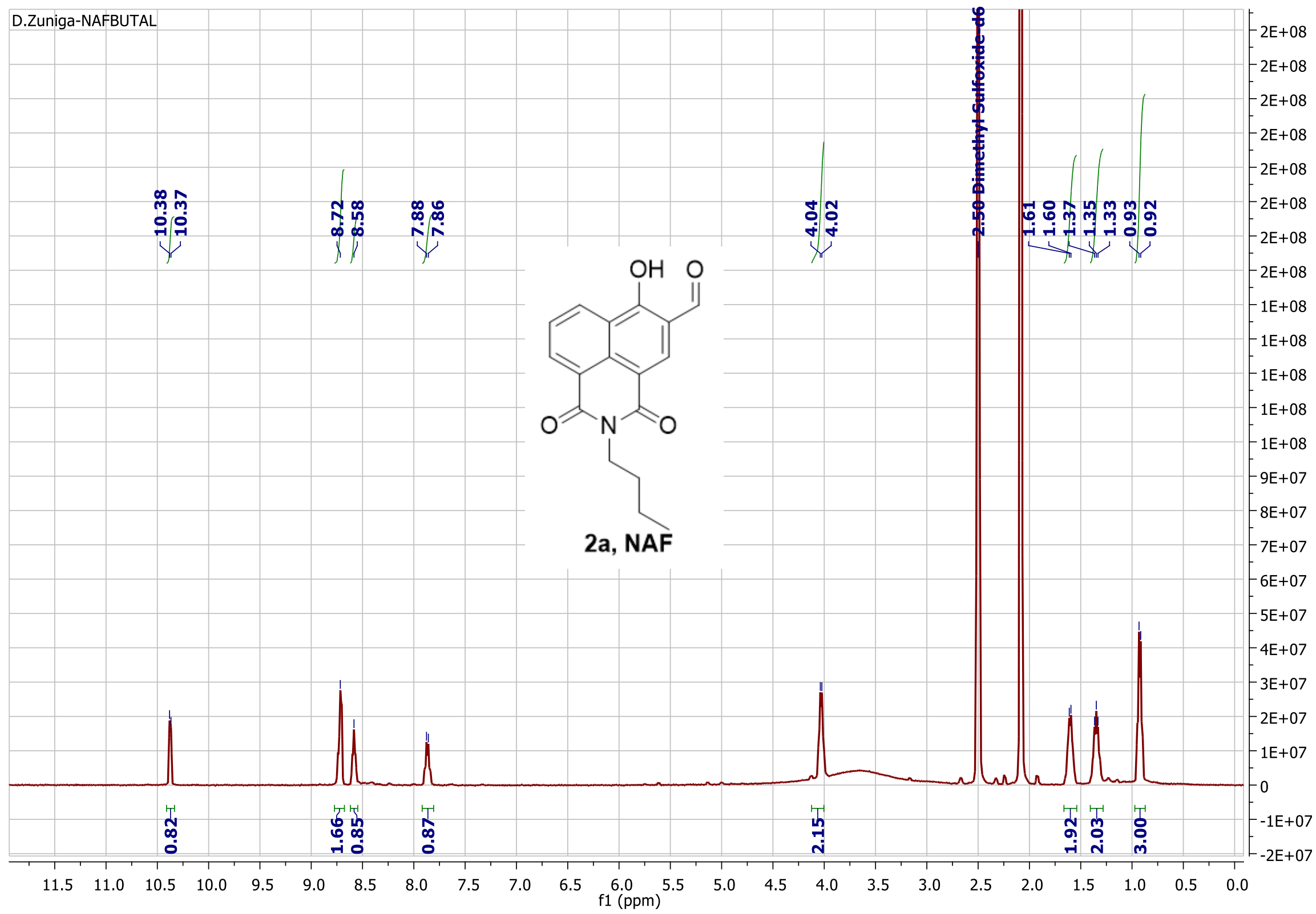


**Figure S11.** $^{1}$**H NMR in DMSO-d**$_{6}$ **of compound 2a (NAF).**

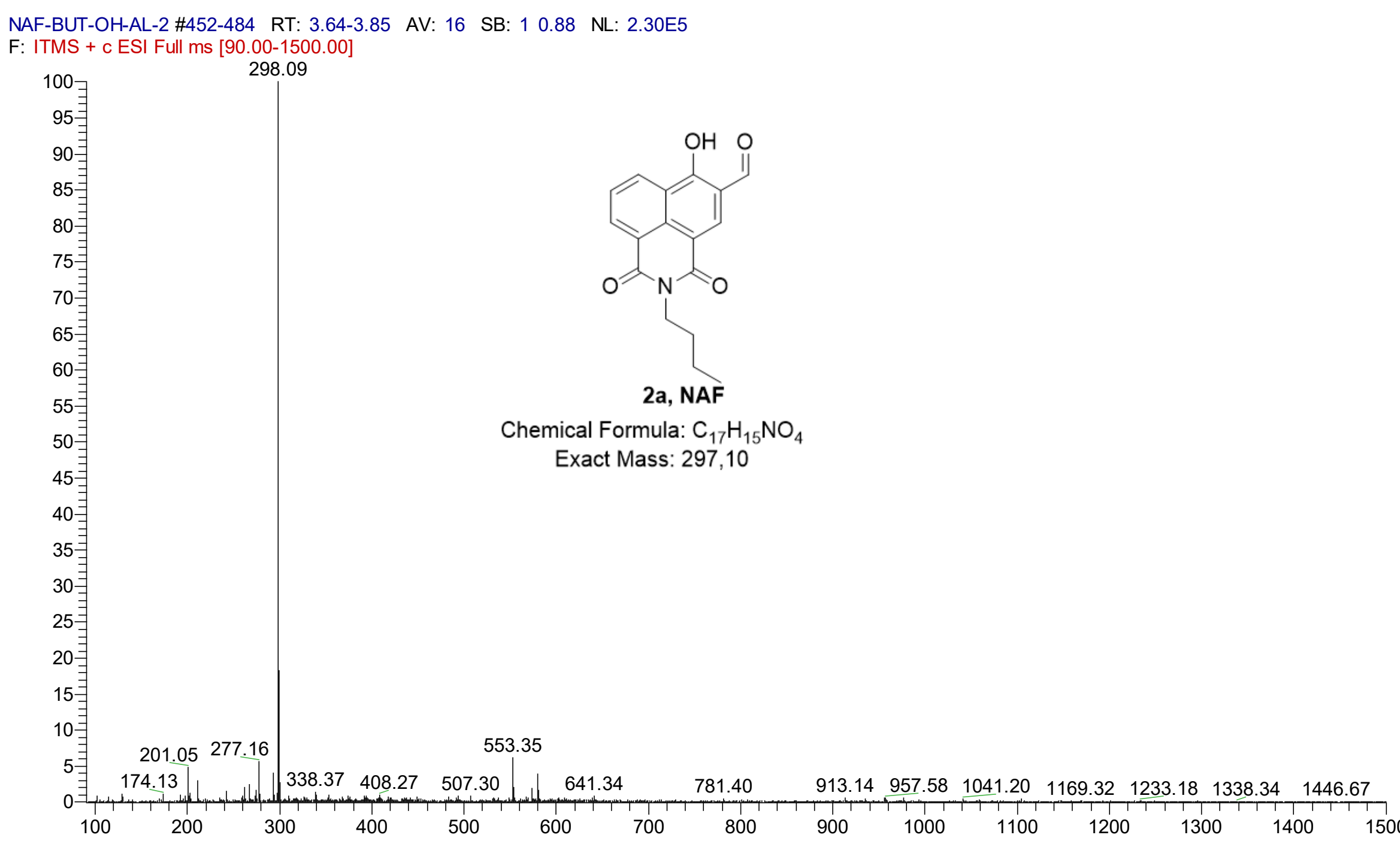


**Figure S12. UHPLC MS/MS in positive mode of compound 2a (NAF).**

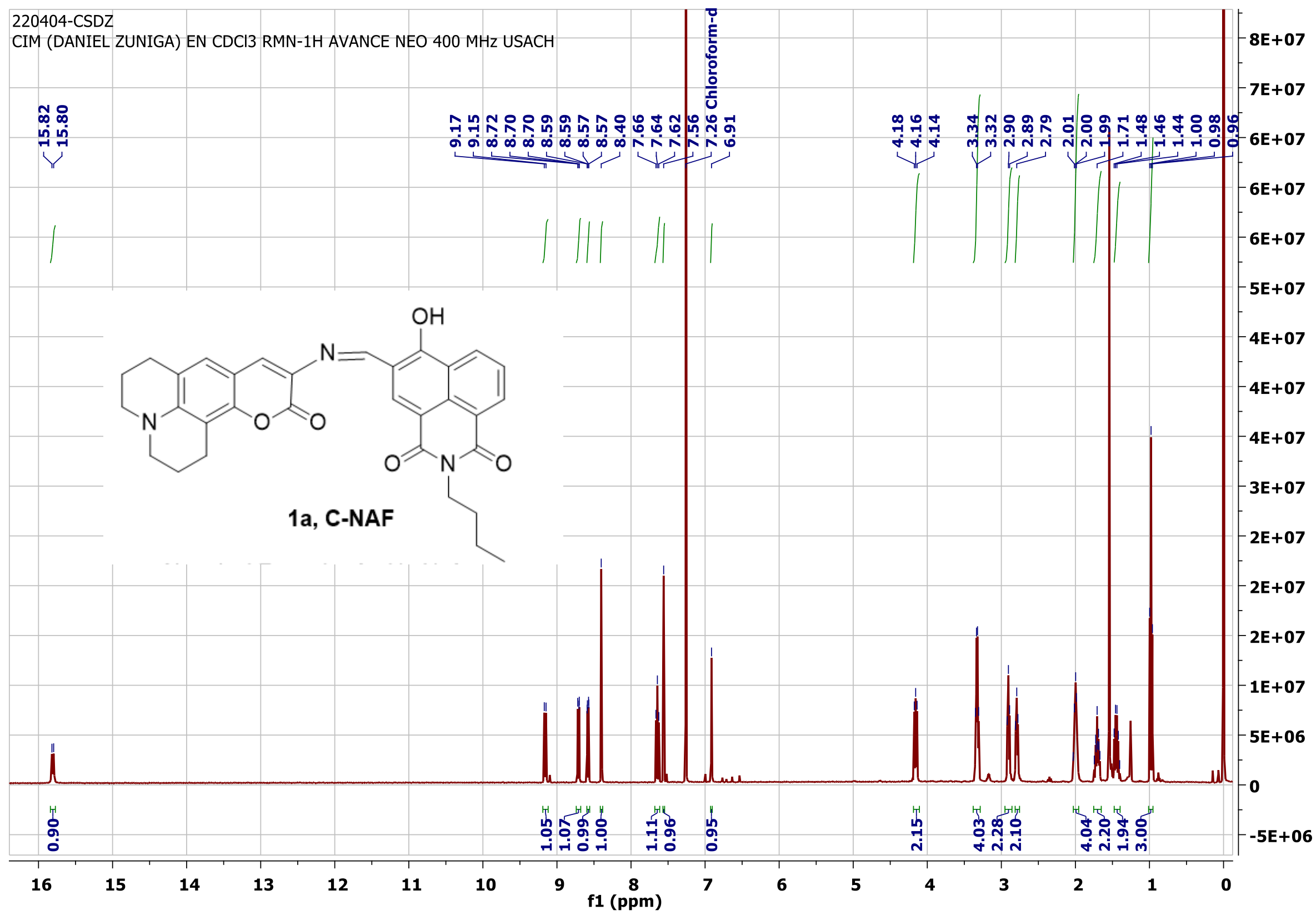


**Figure S13. $^{1}$H NMR in $CDCl_3$ of 1a (C-NAF).**

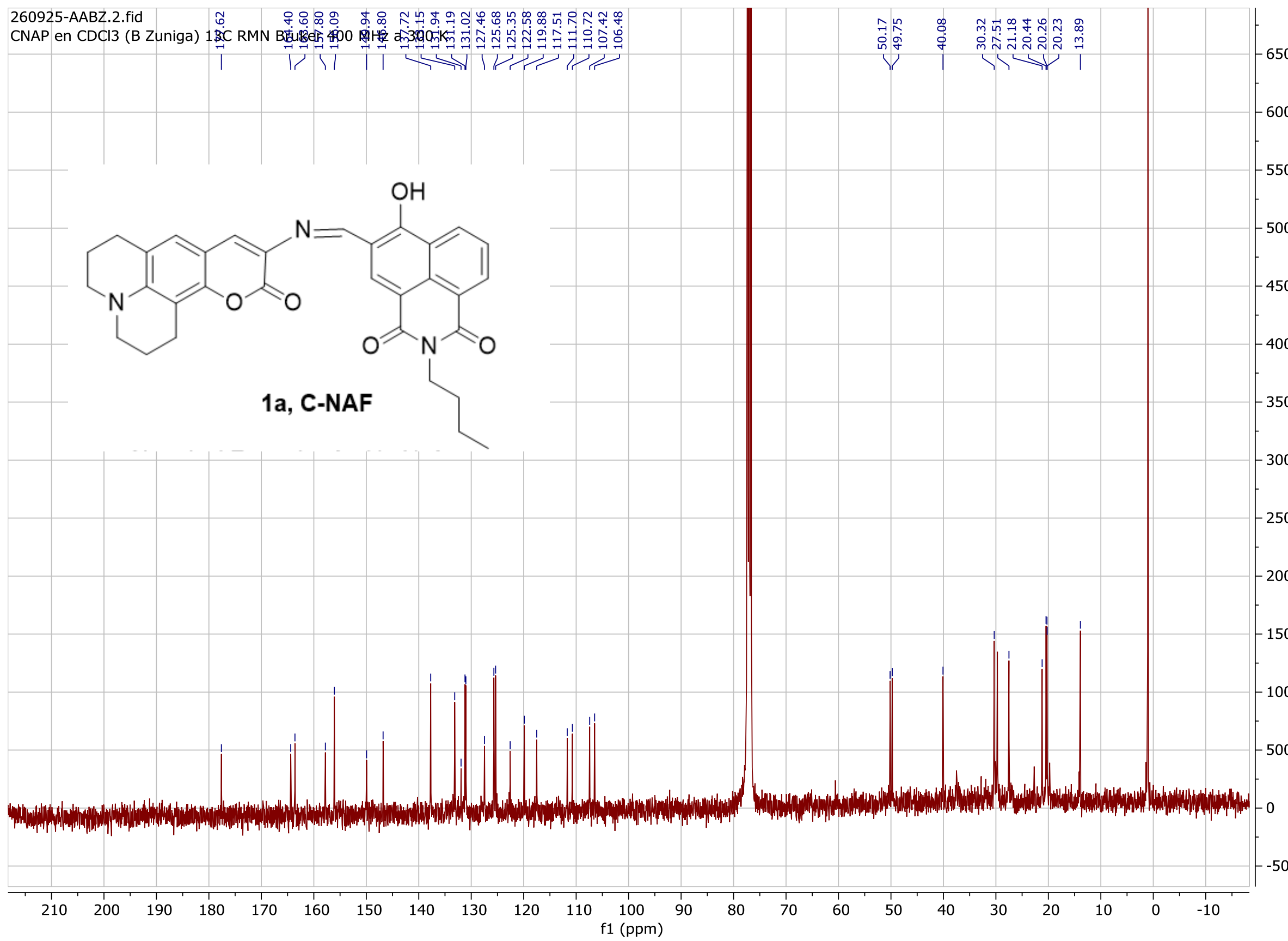


**Figure S14. $^{13}C$ NMR in $CDCl_3$ of 1a (C-NAF).**

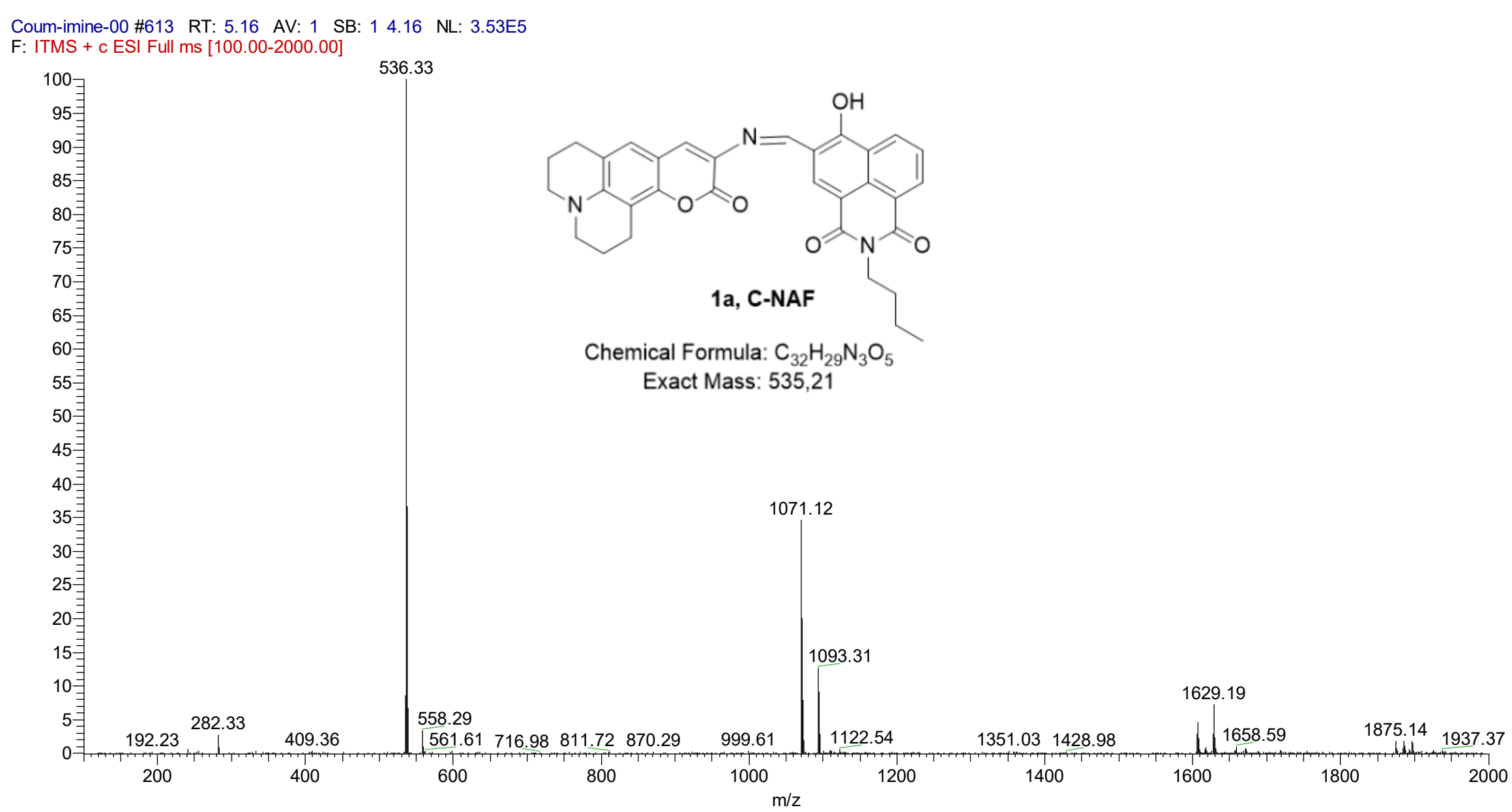


**Figure S15. UHPLC MS/MS in positive mode of 1a (C-NAF).**

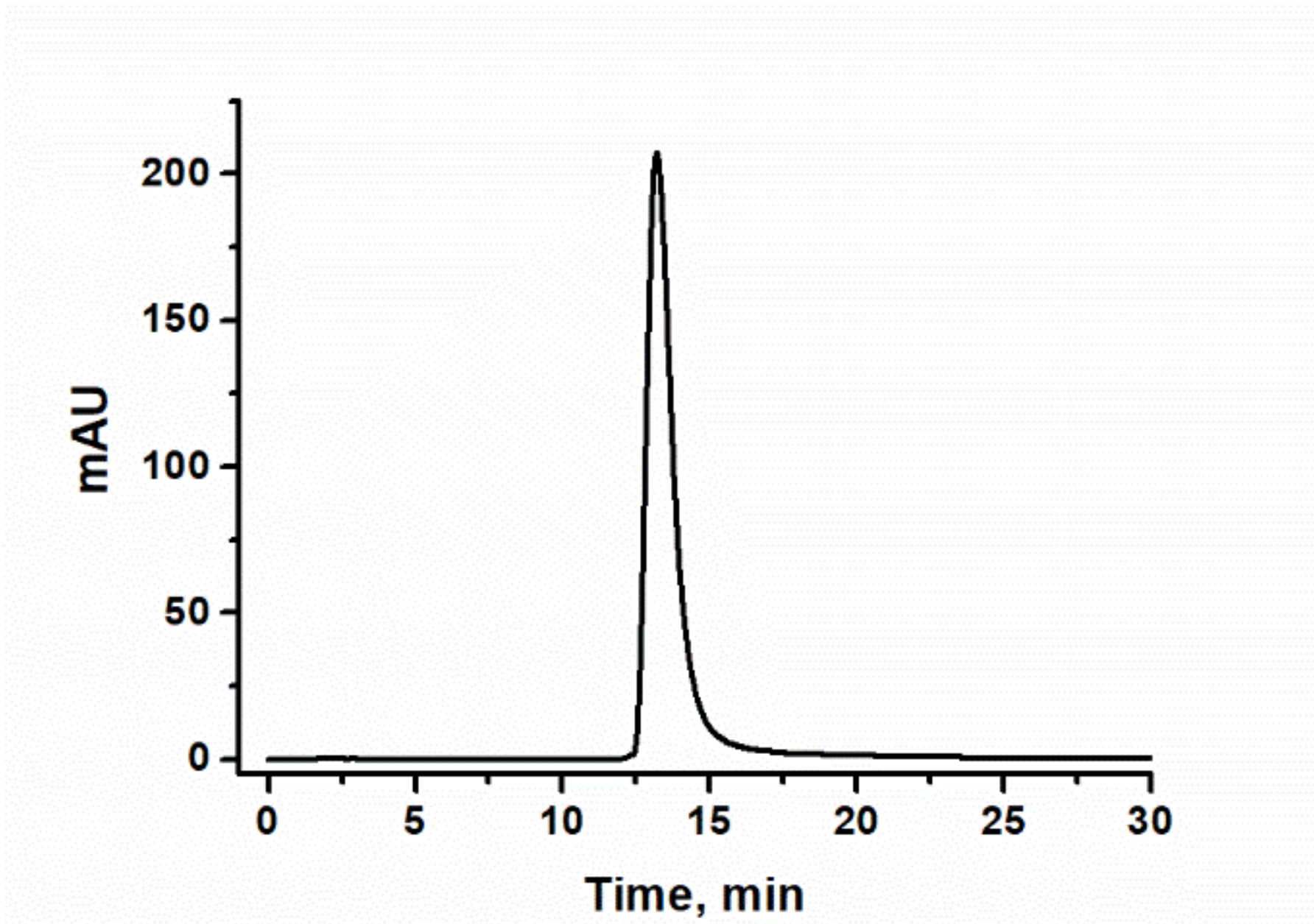


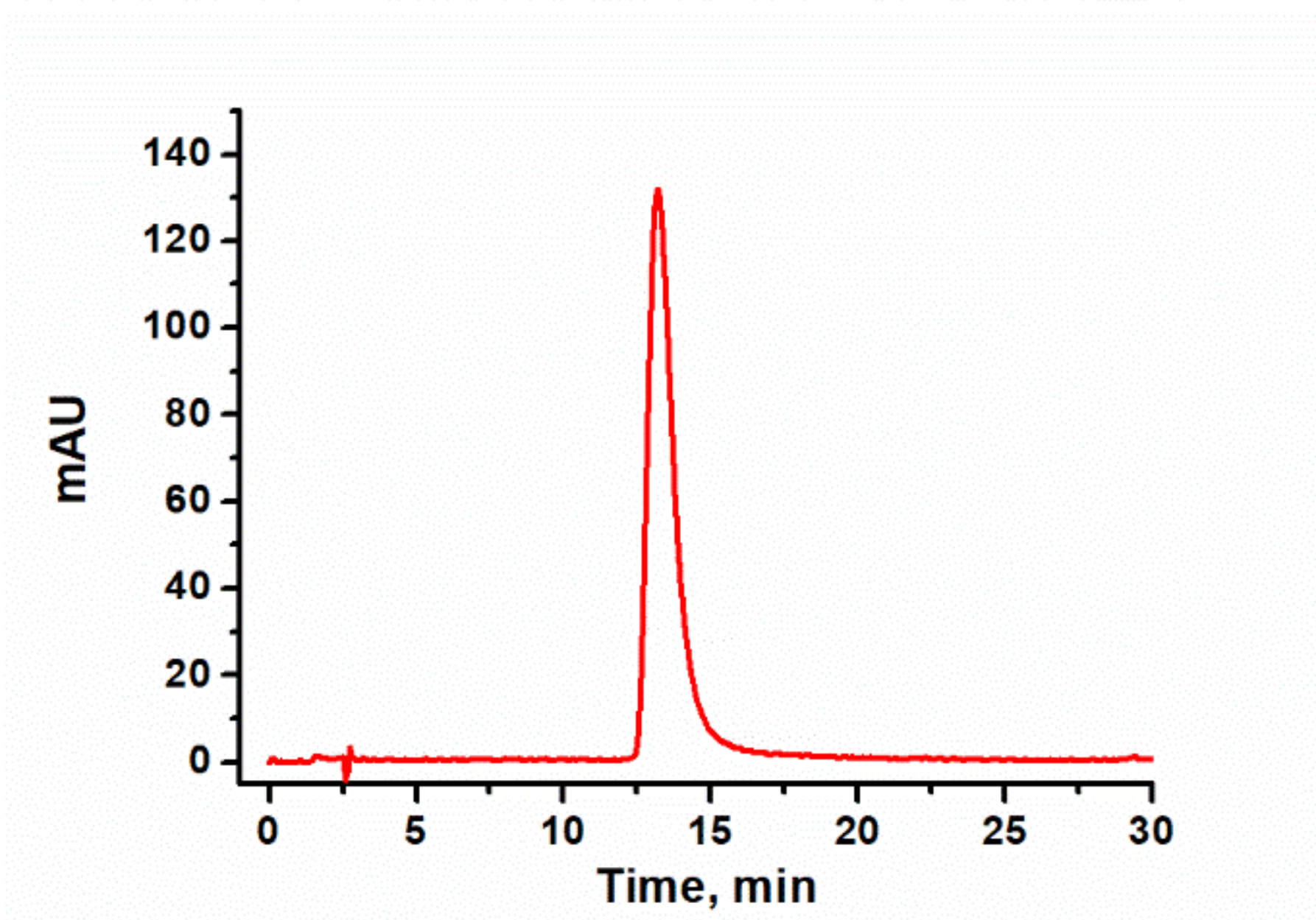


**Figure S16. Chromatogram of C-NAF obtained by HPLC-DAD. The sample was thermostated at 25 °C and analyzed under isocratic conditions using 100% acetonitrile as the mobile phase at a flow rate of 1.0 mL min$^{-1}$. Top: detection at 550 nm; bottom: detection at 220 nm.**

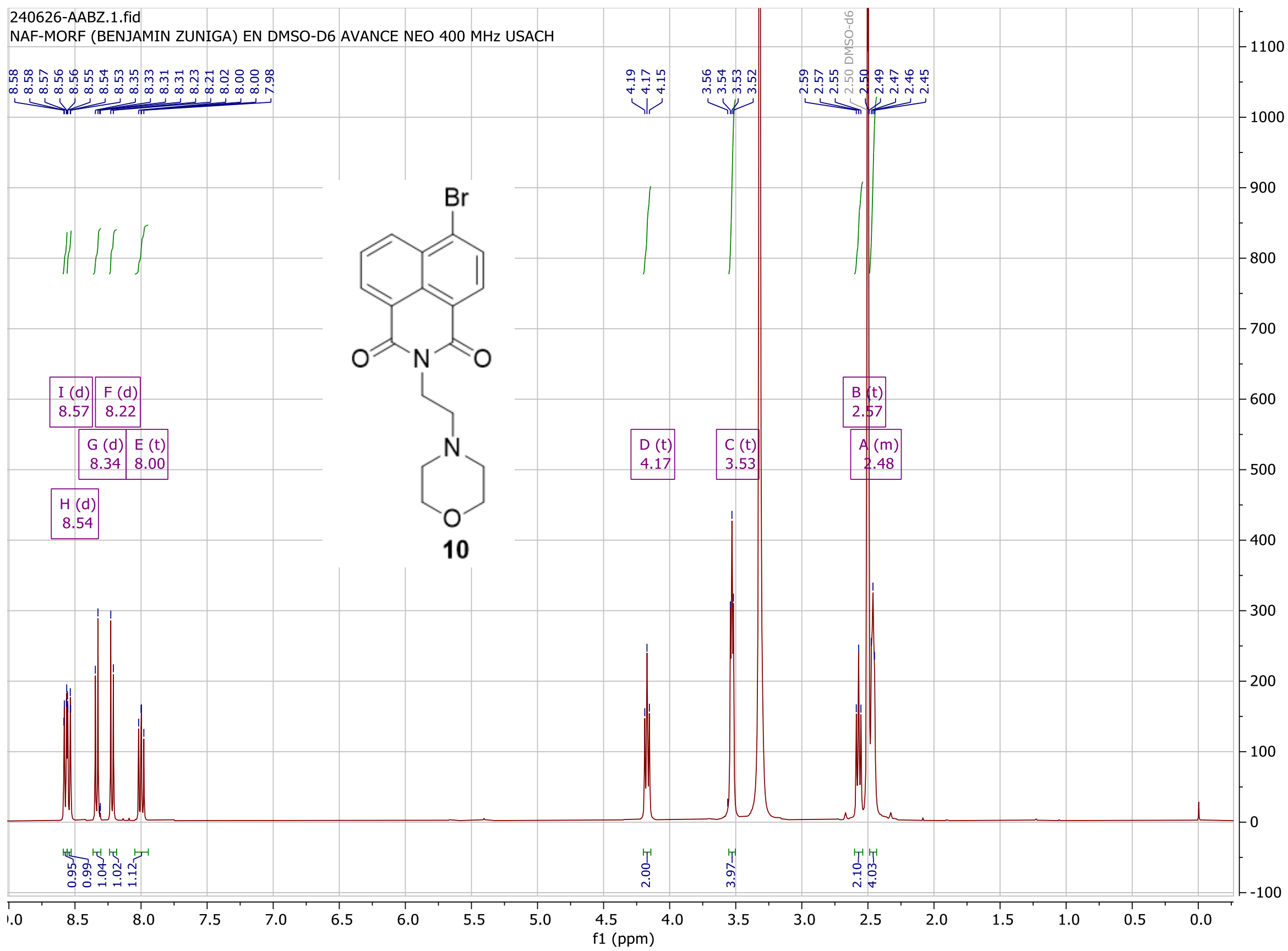

**Figure S17. $^1$H NMR in DMSO-d$_6$ of compound 10.**

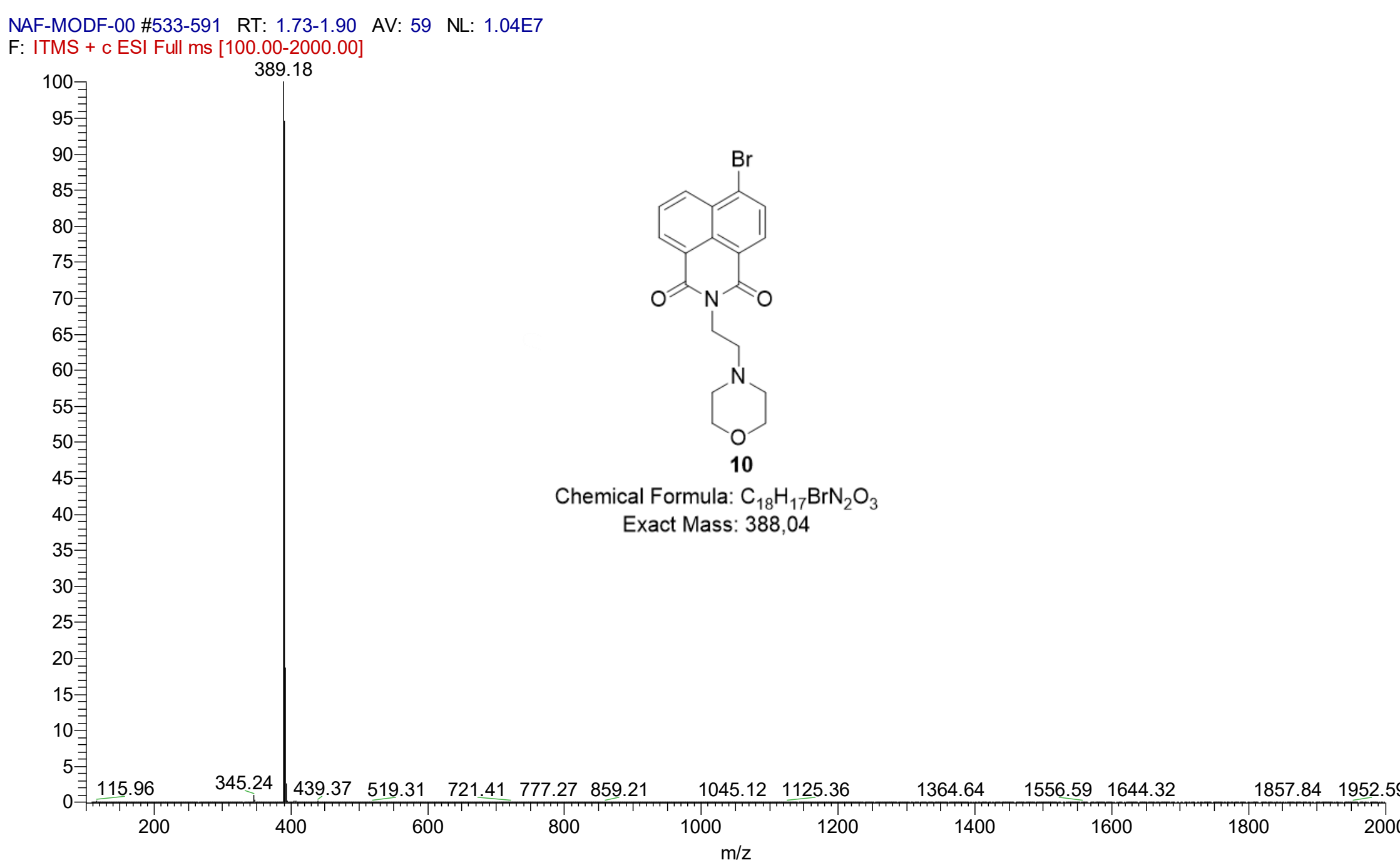

**Figure S18. UHPLC MS/MS in positive mode of compound 10.**

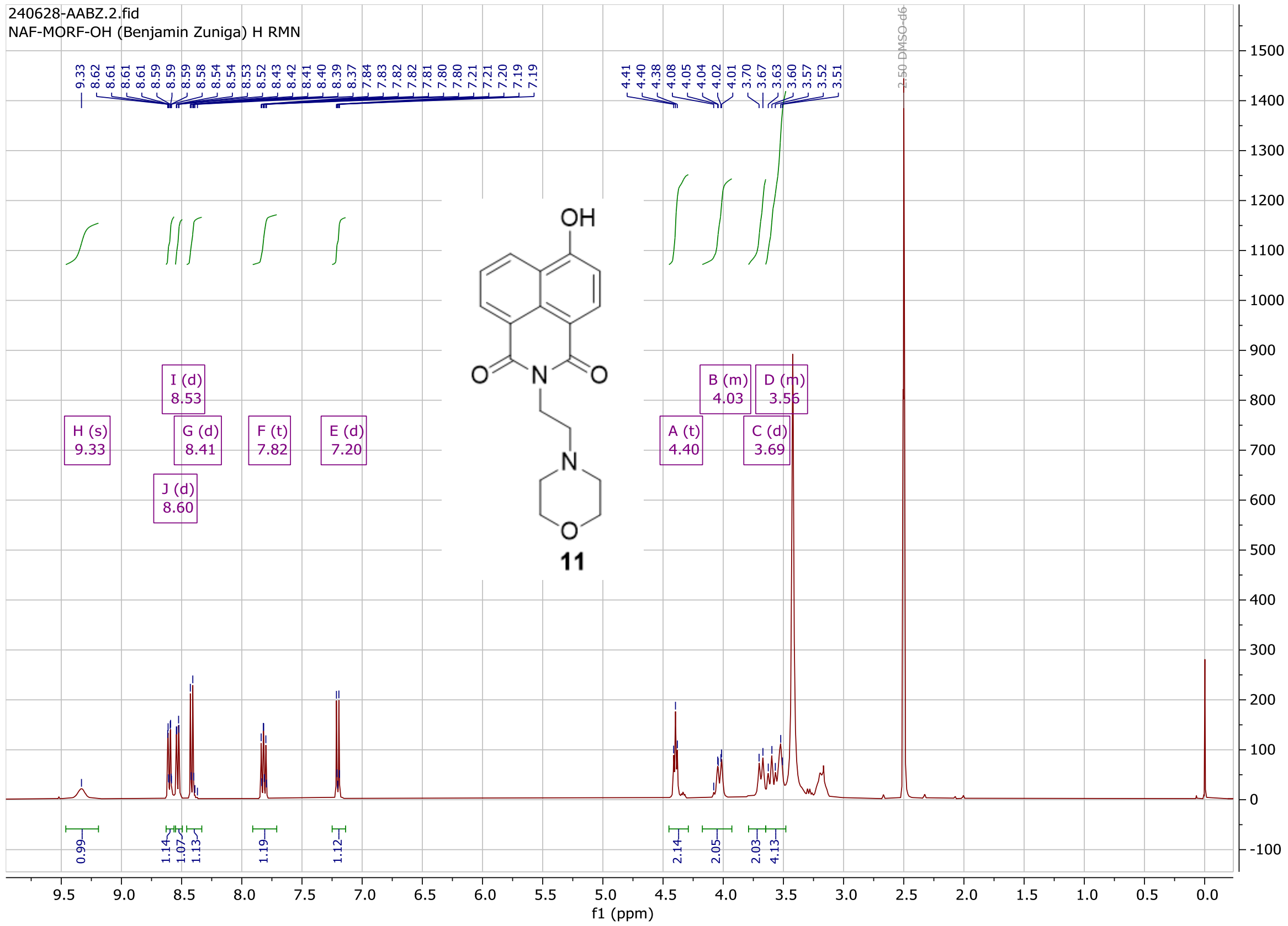


**Figure S19.** [1]**H NMR in DMSO-d**$_6$ **of compound 11.**

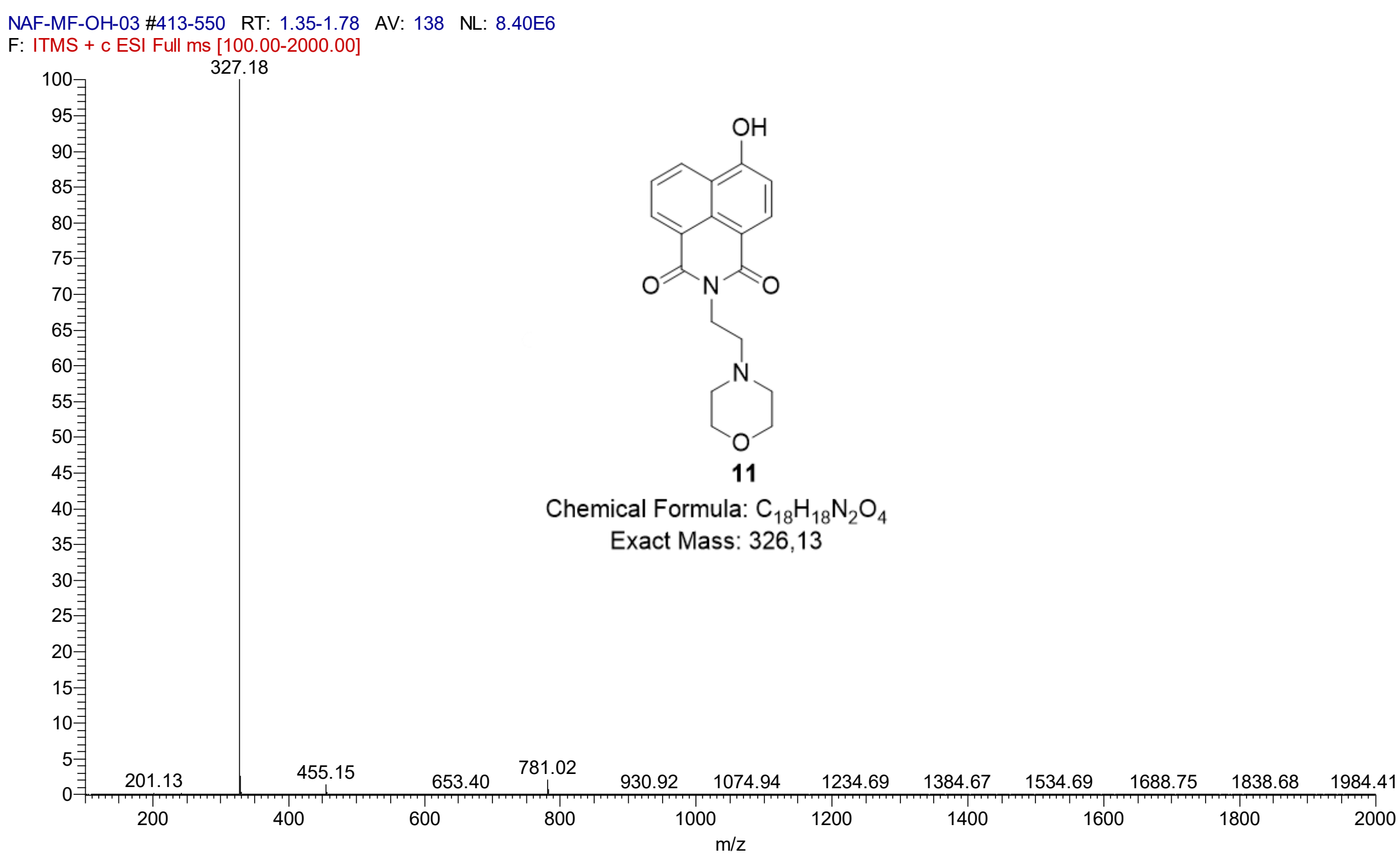


**Figure S20.UHPLC MS/MS in positive mode of compound 11.**

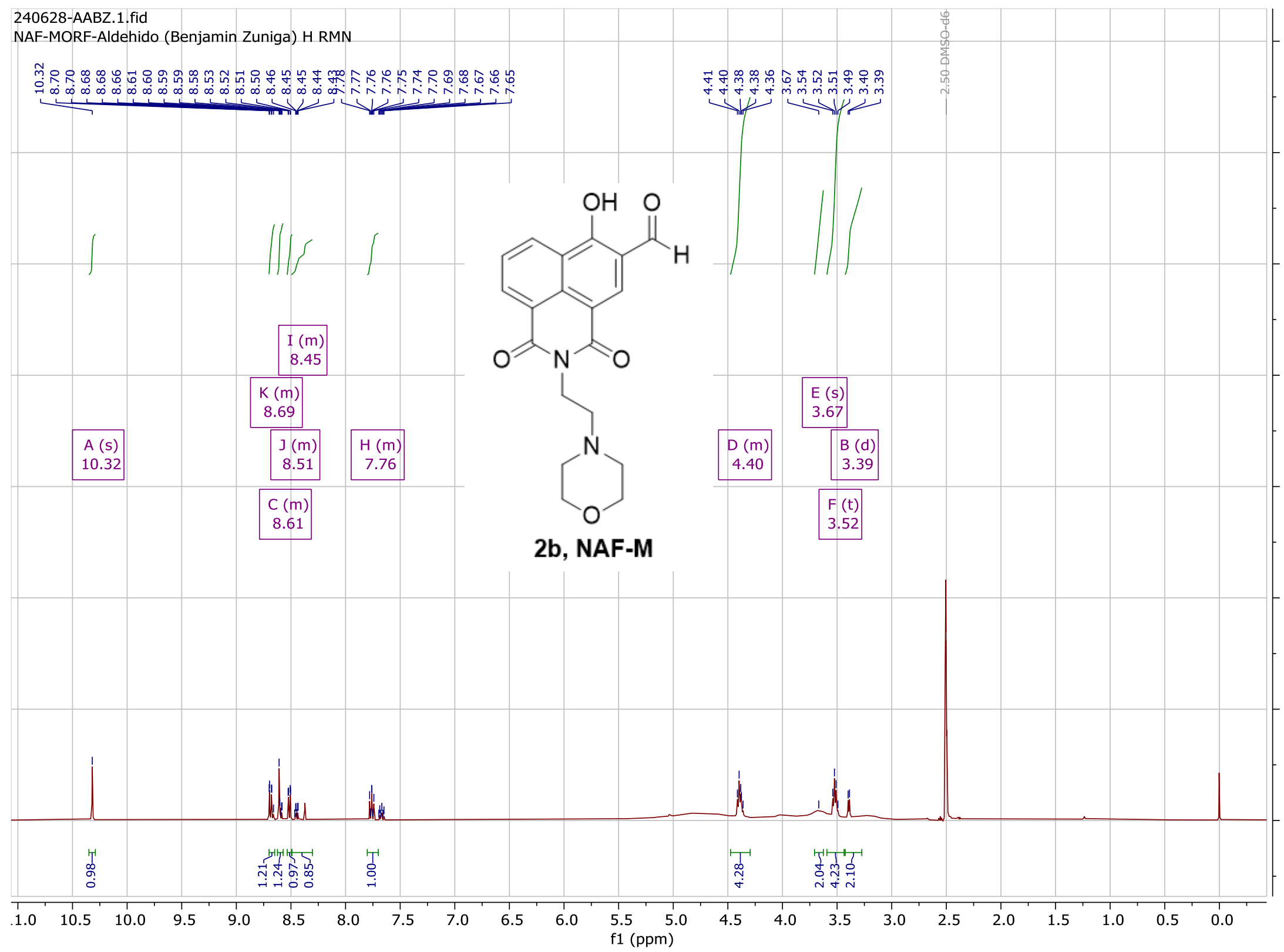


**Figure S21. $^{1}H$ NMR in DMSO-$d_6$ of compound 2b (NAF-M).**

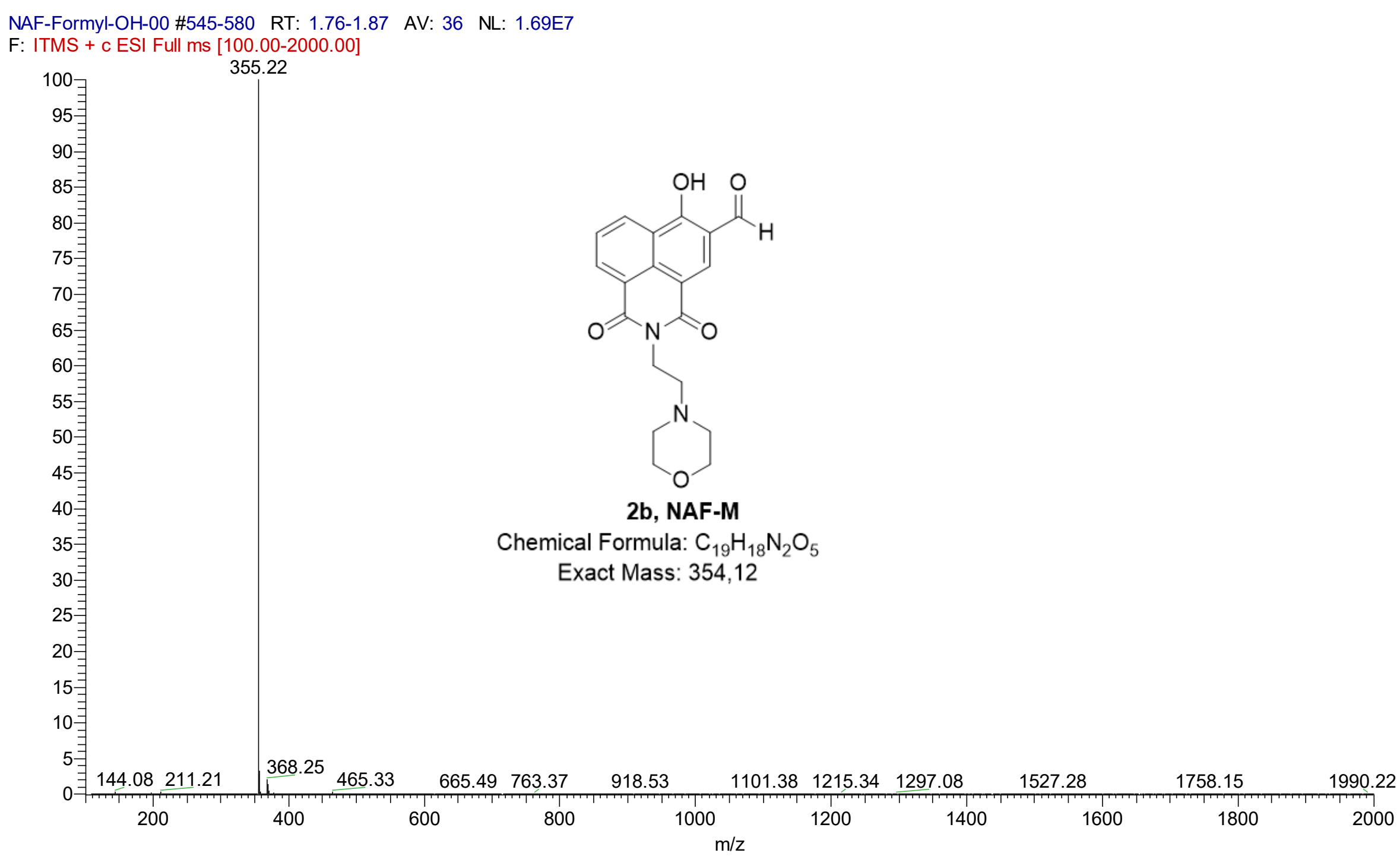


**Figure S22. UHPLC MS/MS in positive mode of compound 2b (NAF-M).**

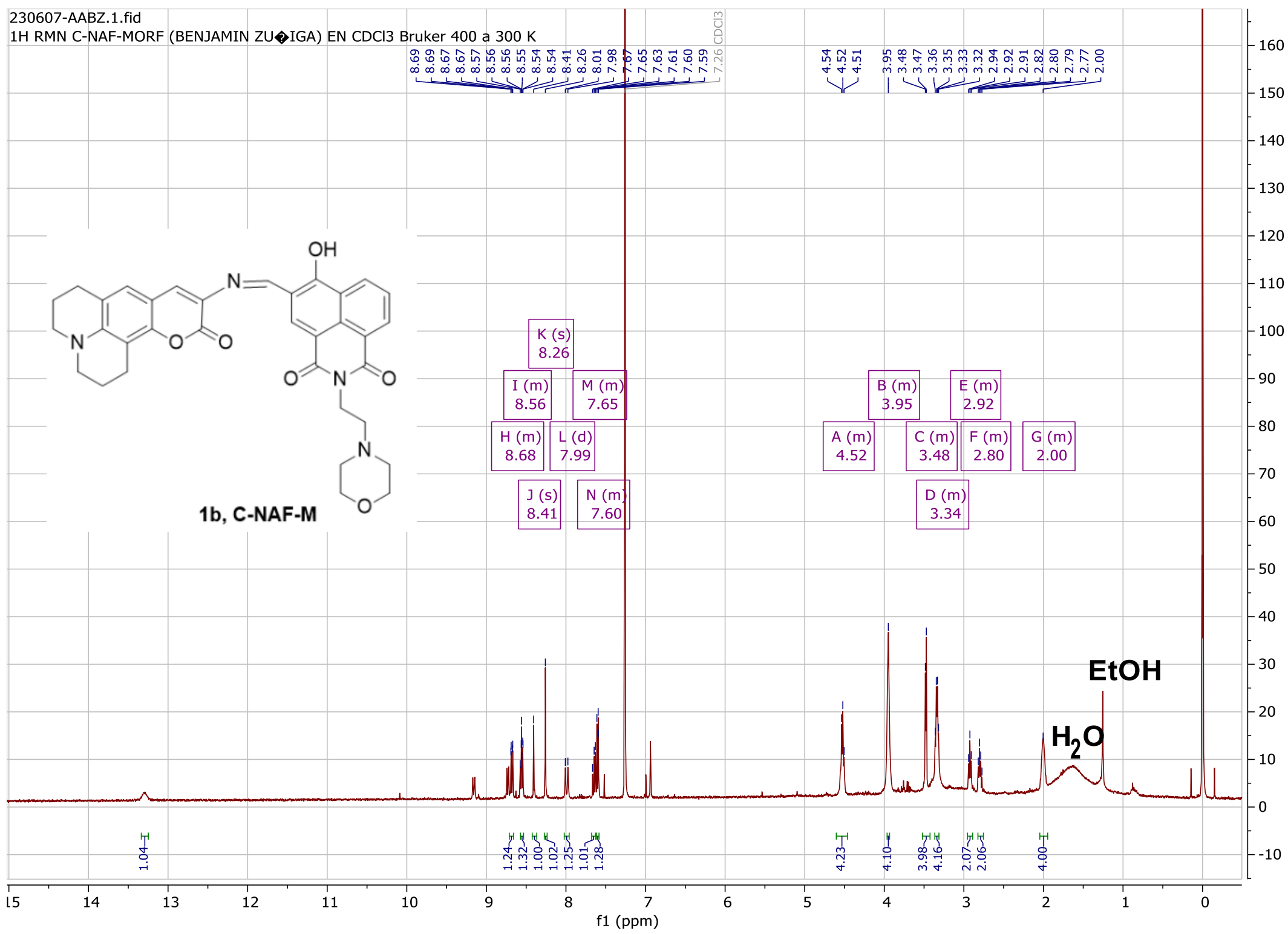

**Figure S23. $^{1}H$ NMR in $CDCl_3$ of compound 1b (C-NAF-M).**

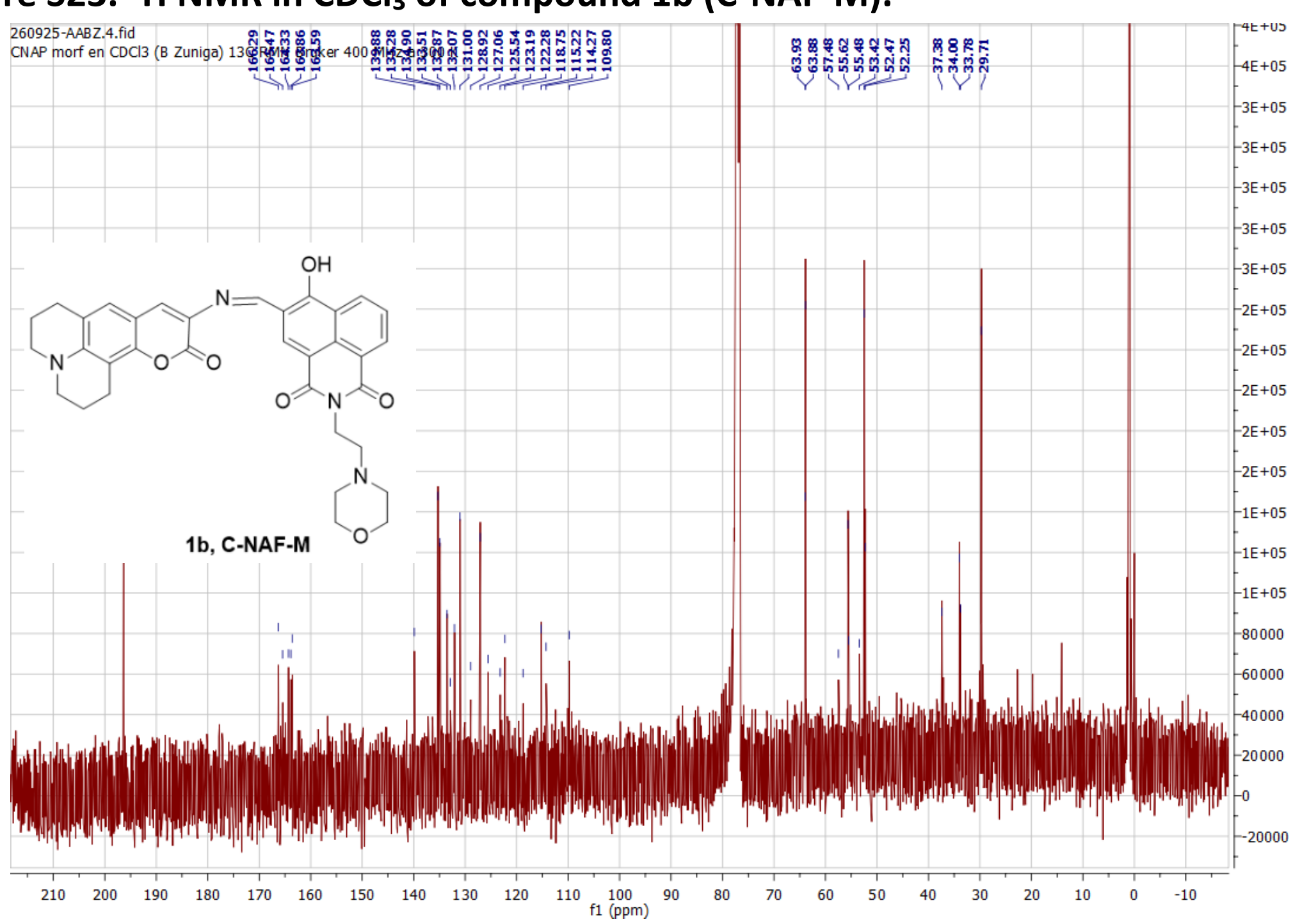

**Figure S24. $^{13}C$ NMR in $CDCl_3$ of compound 1b (C-NAF-M).**

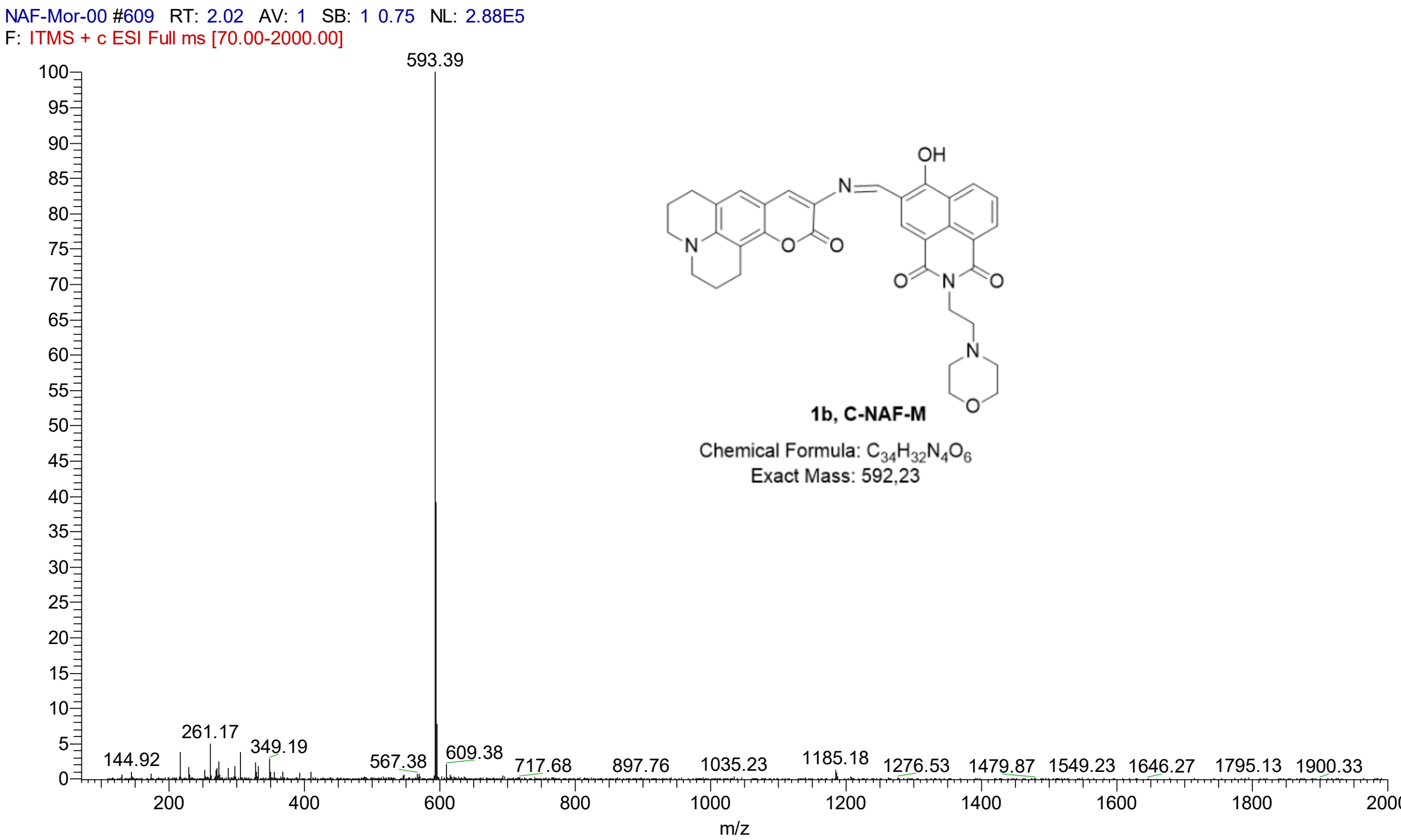


**Figure S25. UHPLC MS/MS in positive mode of compound 1b (C-NAF-M).**

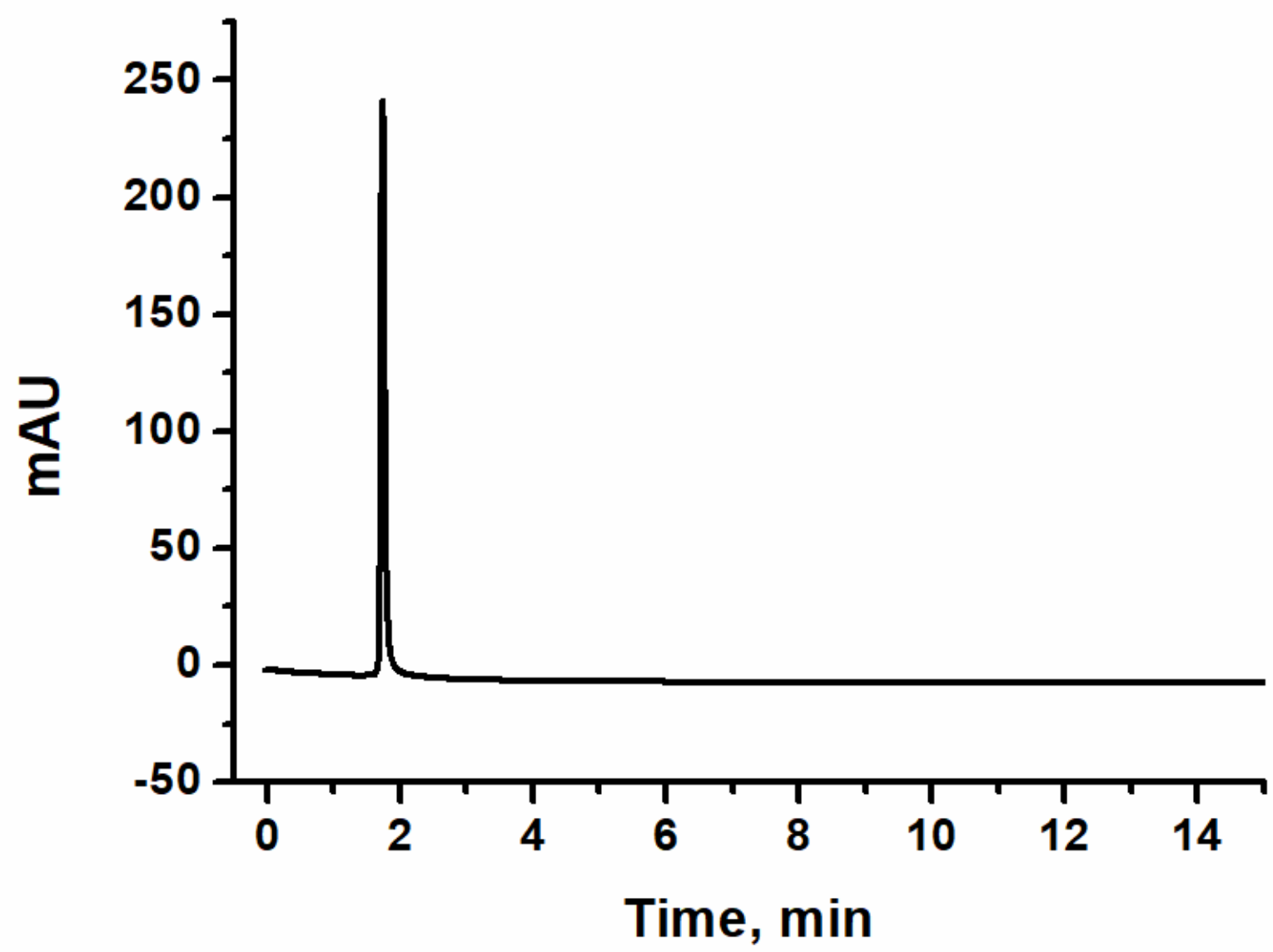


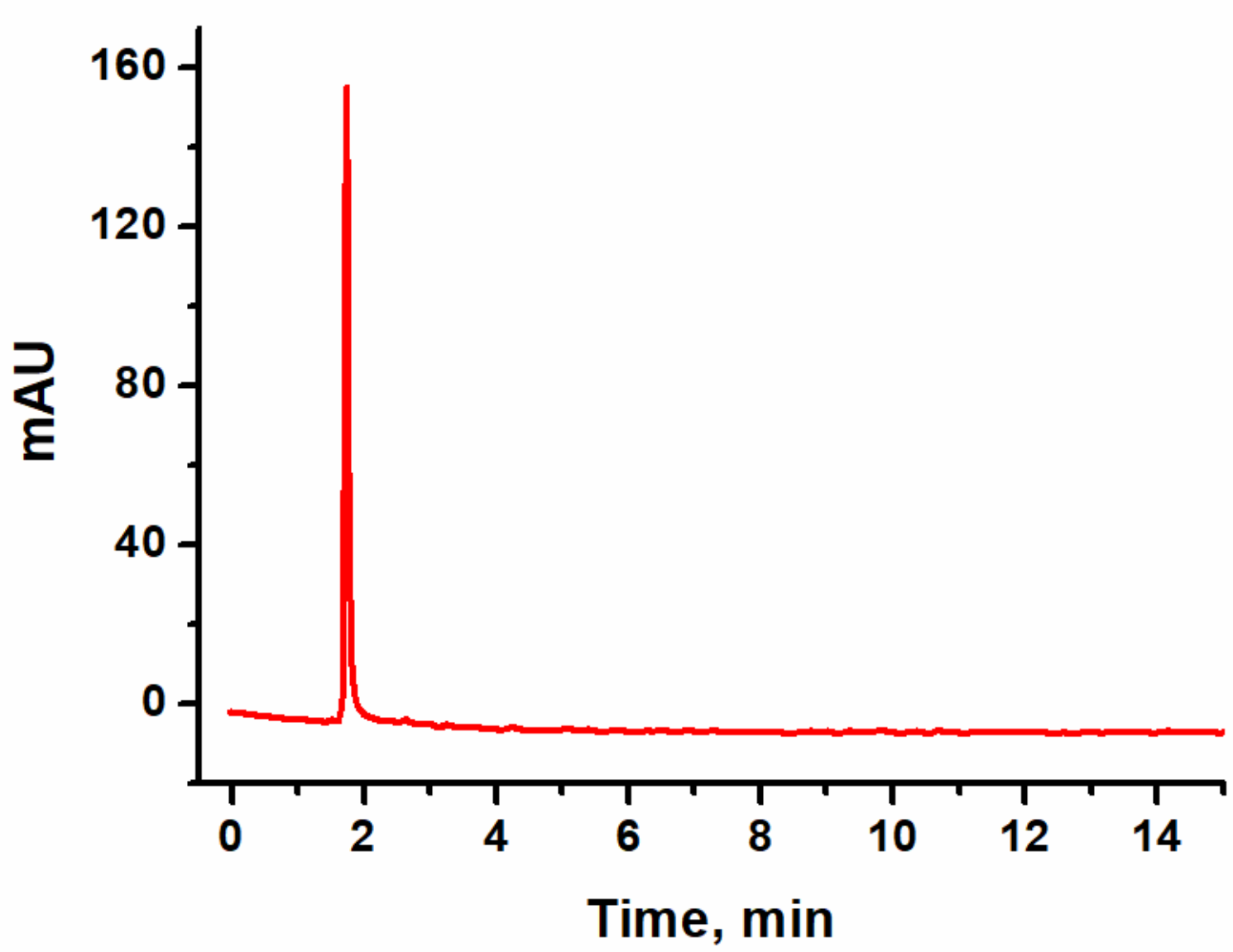


**Figure S26. Chromatogram of C-NAF-M obtained by HPLC-DAD. The sample was thermostated at 25 °C and analyzed under isocratic conditions using 100% acetonitrile as the mobile phase at a flow rate of 1.0 mL min$^{-1}$. Top: detection at 550 nm; bottom: detection at 220 nm.**

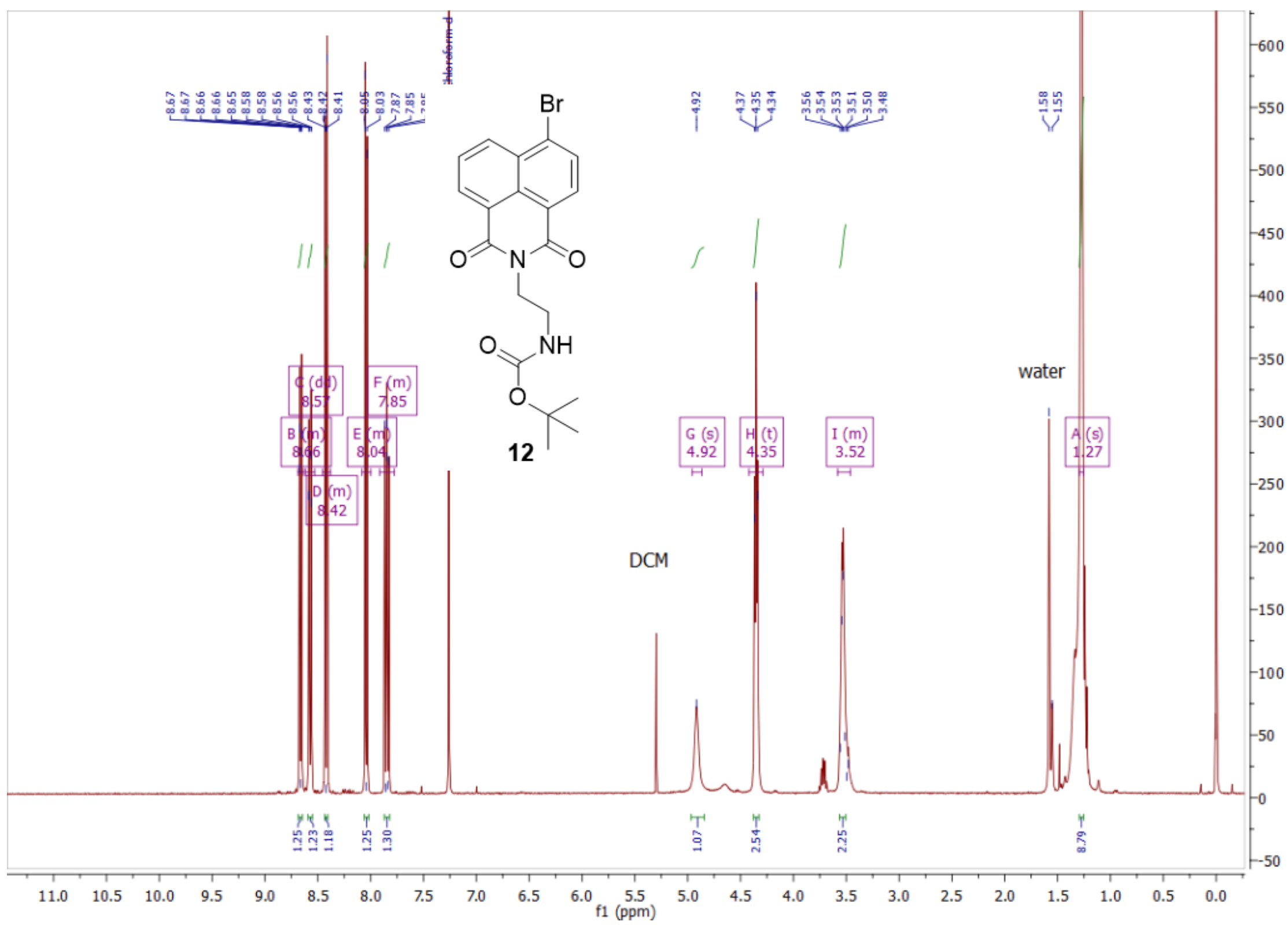


**Figure S27. $^{1}$H-NMR in $CDCl_3$ of compound 12.**

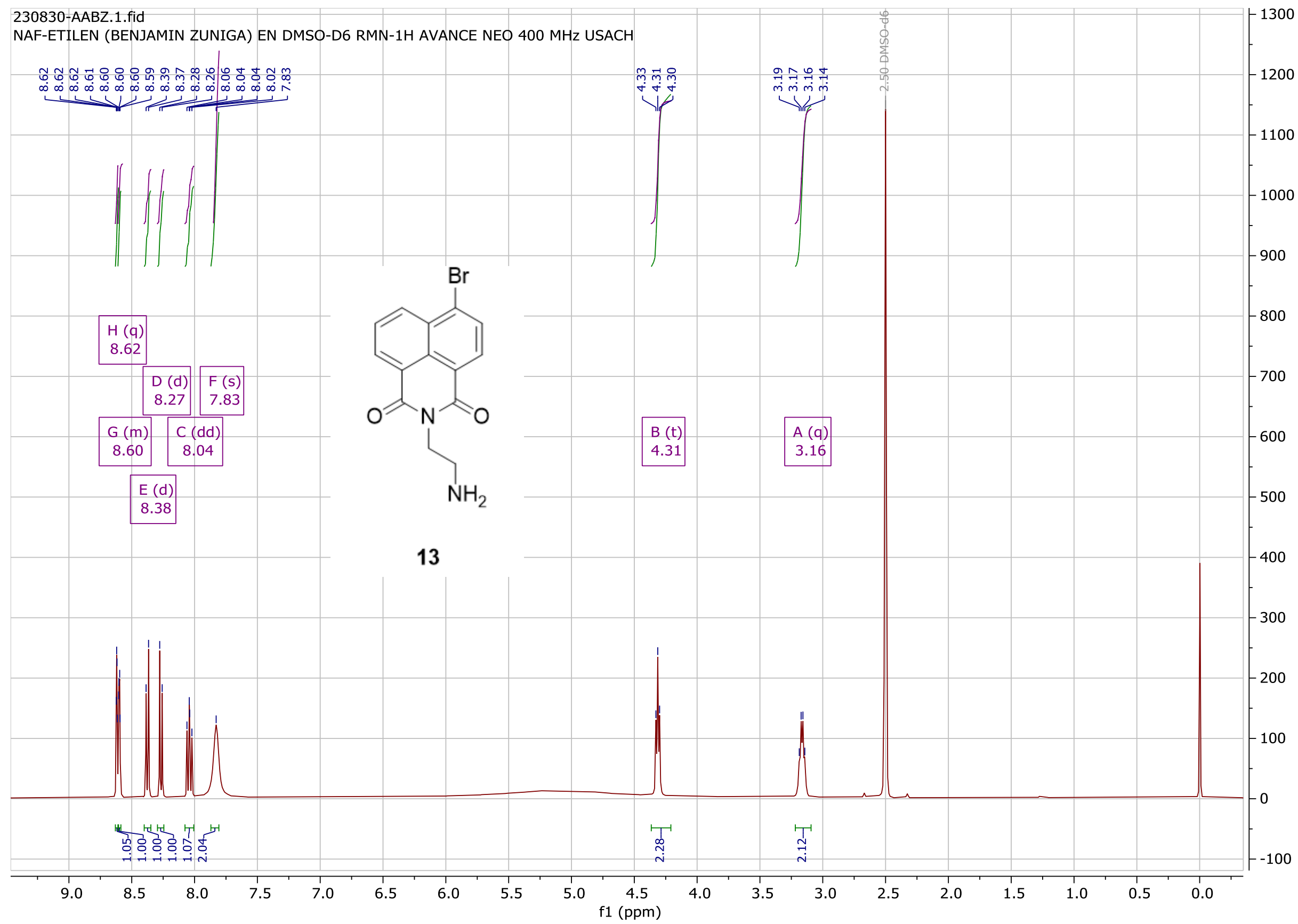


**Figure S28. $^{1}H$ NMR in DMSO-d$_6$ of compound 13.**

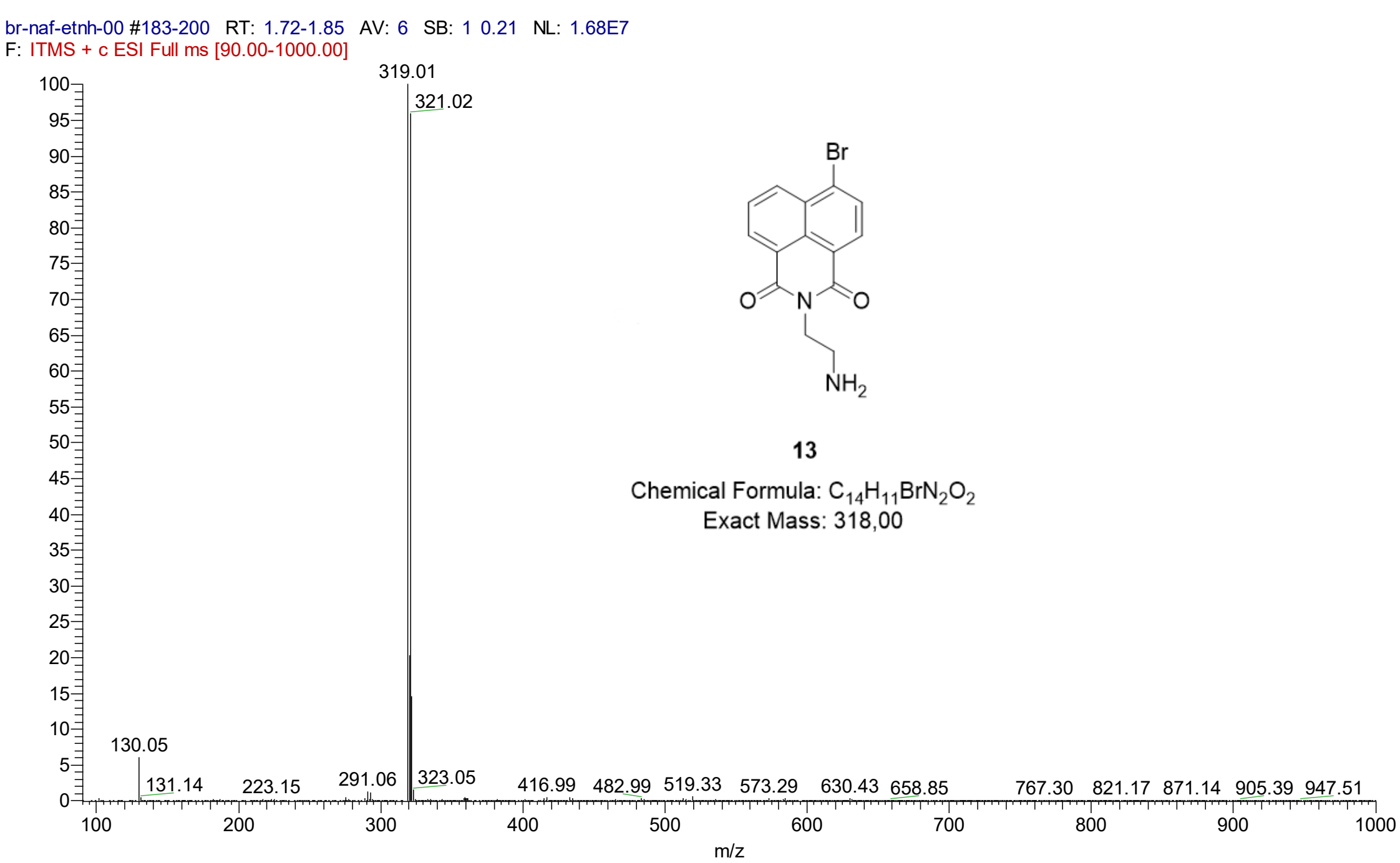


**Figure S29. UHPLC MS/MS in positive mode of compound 13.**

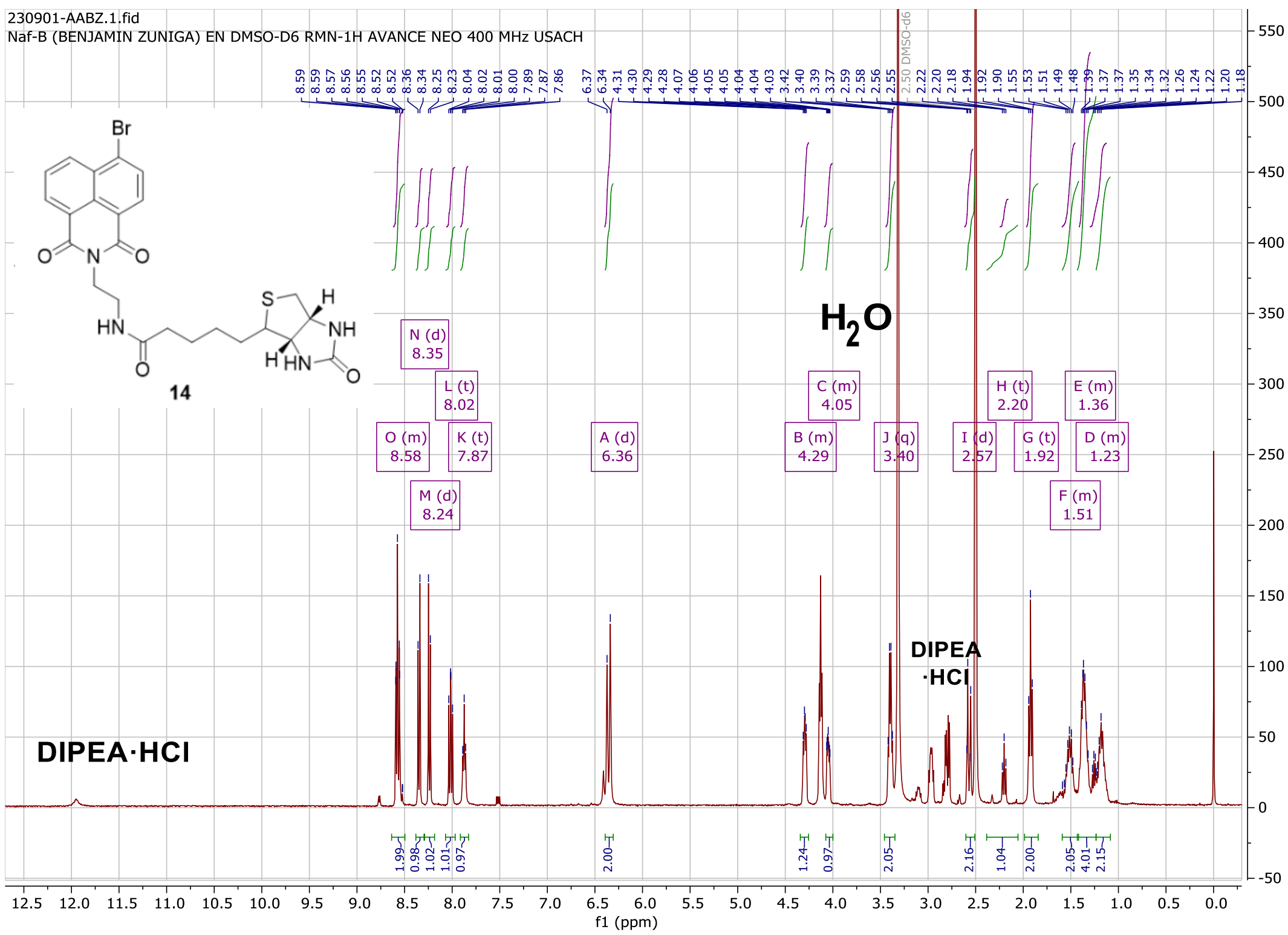


**Figure S30. $^{1}H$ NMR in DMSO-$d_6$ of compound 14.**

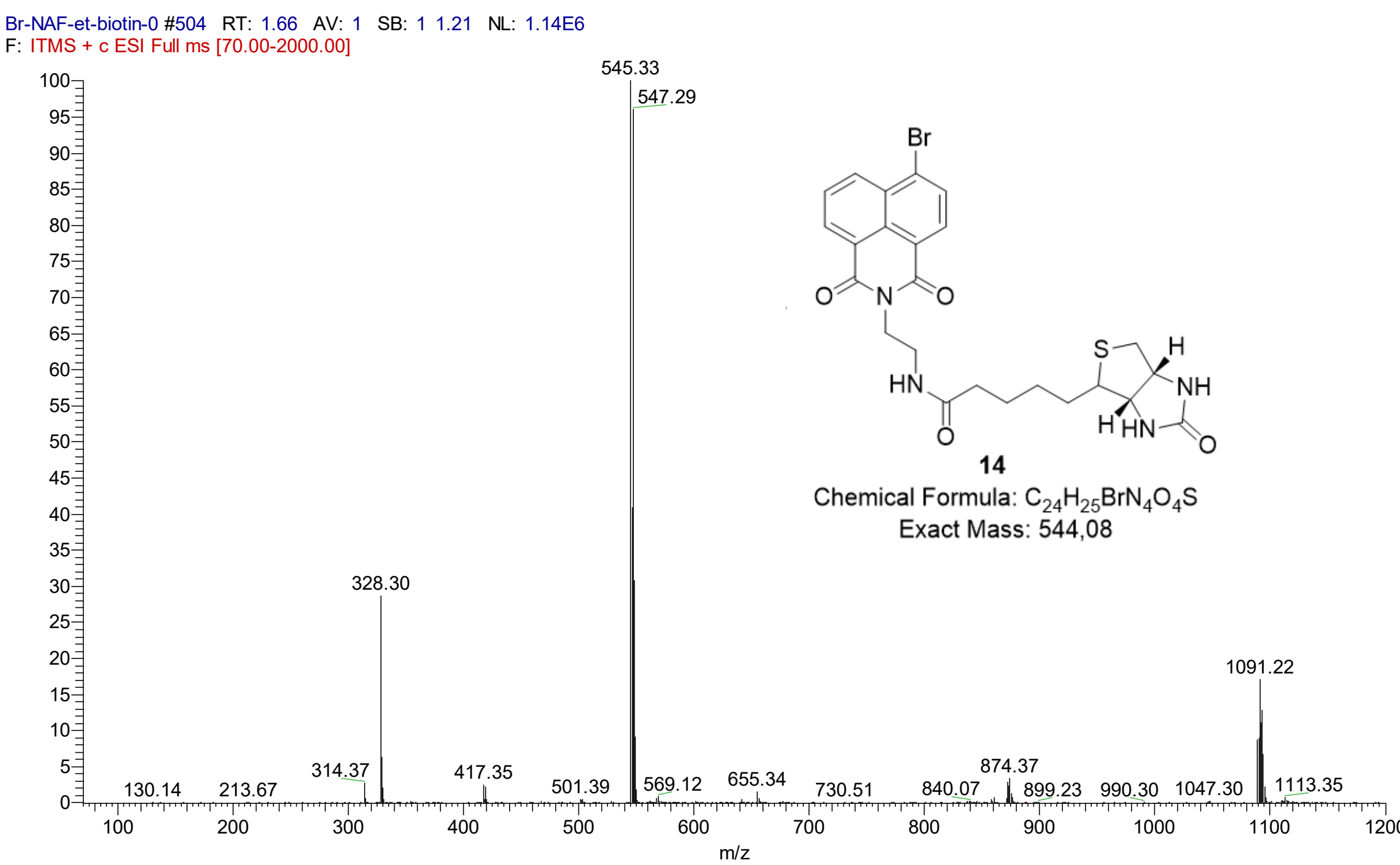


**Figure S31. UHPLC MS/MS in positive mode of compound 14.**

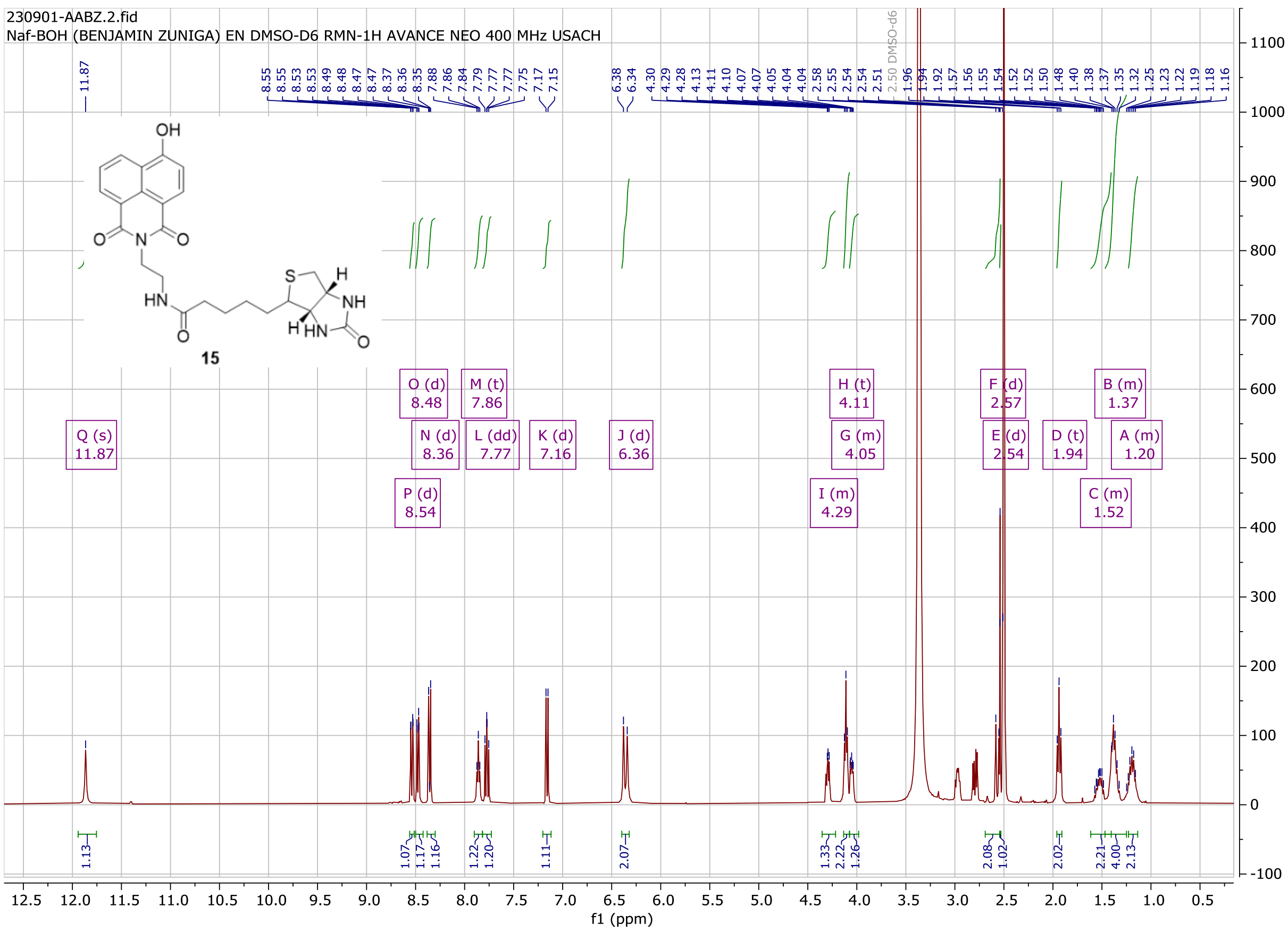


**Figure S32.** $^1$**H-NMR in DMSO-d$_6$ of compound 15.**

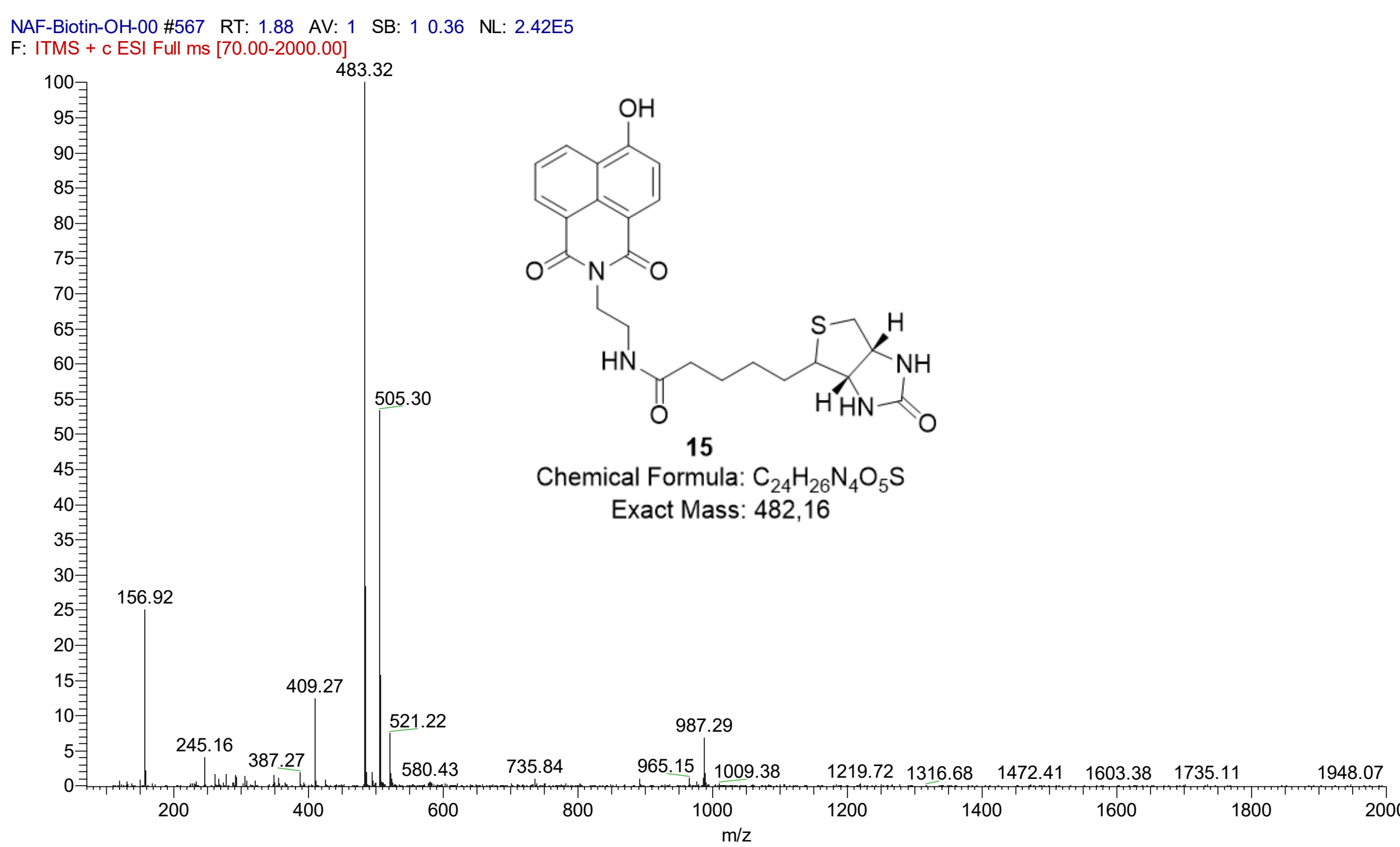


**Figure S33. UHPLC MS/MS in positive mode of compound 15.**

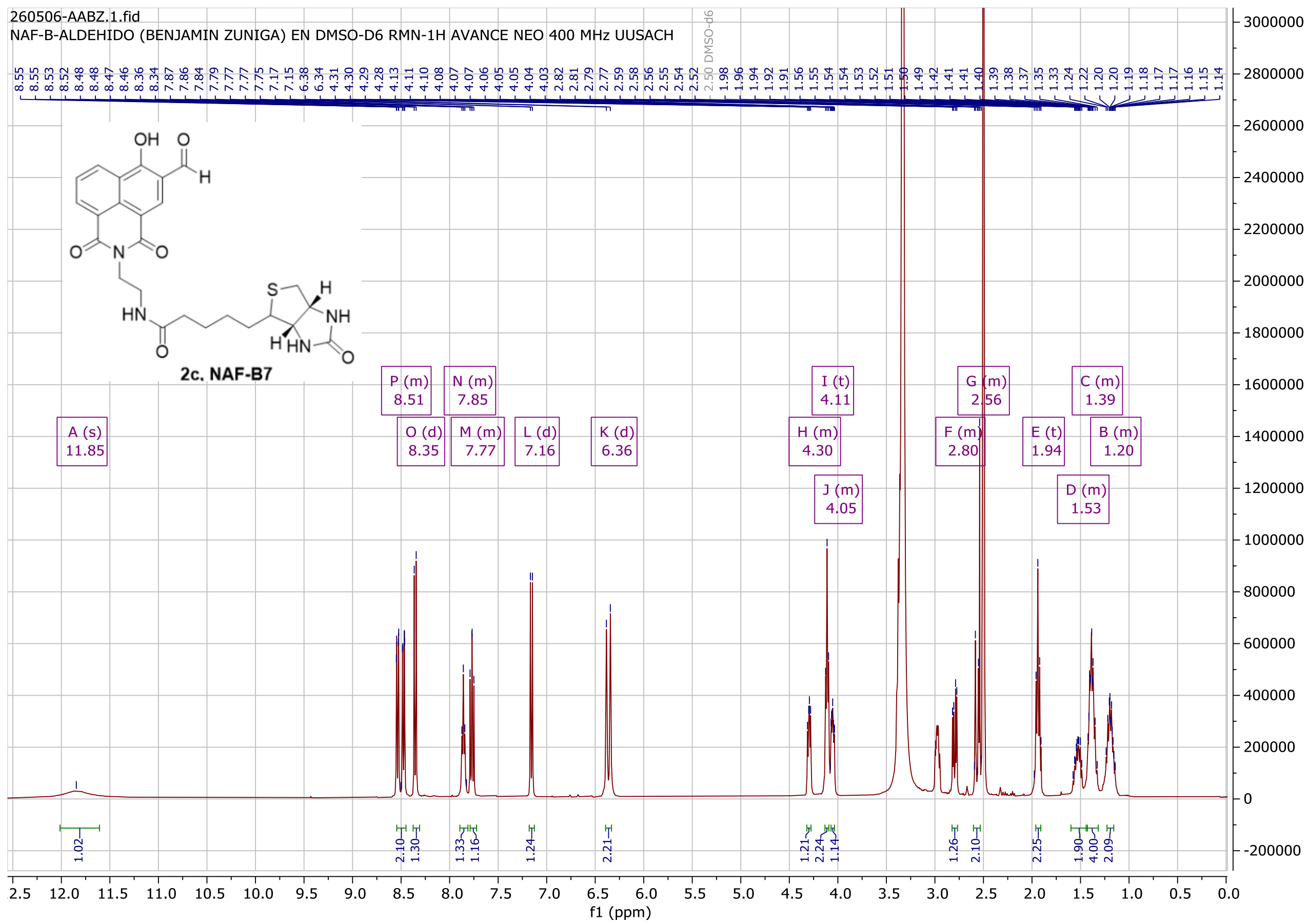


**Figure S34. 1H NMR in DMSO-d6 of compound 2c, NAF-B7.**

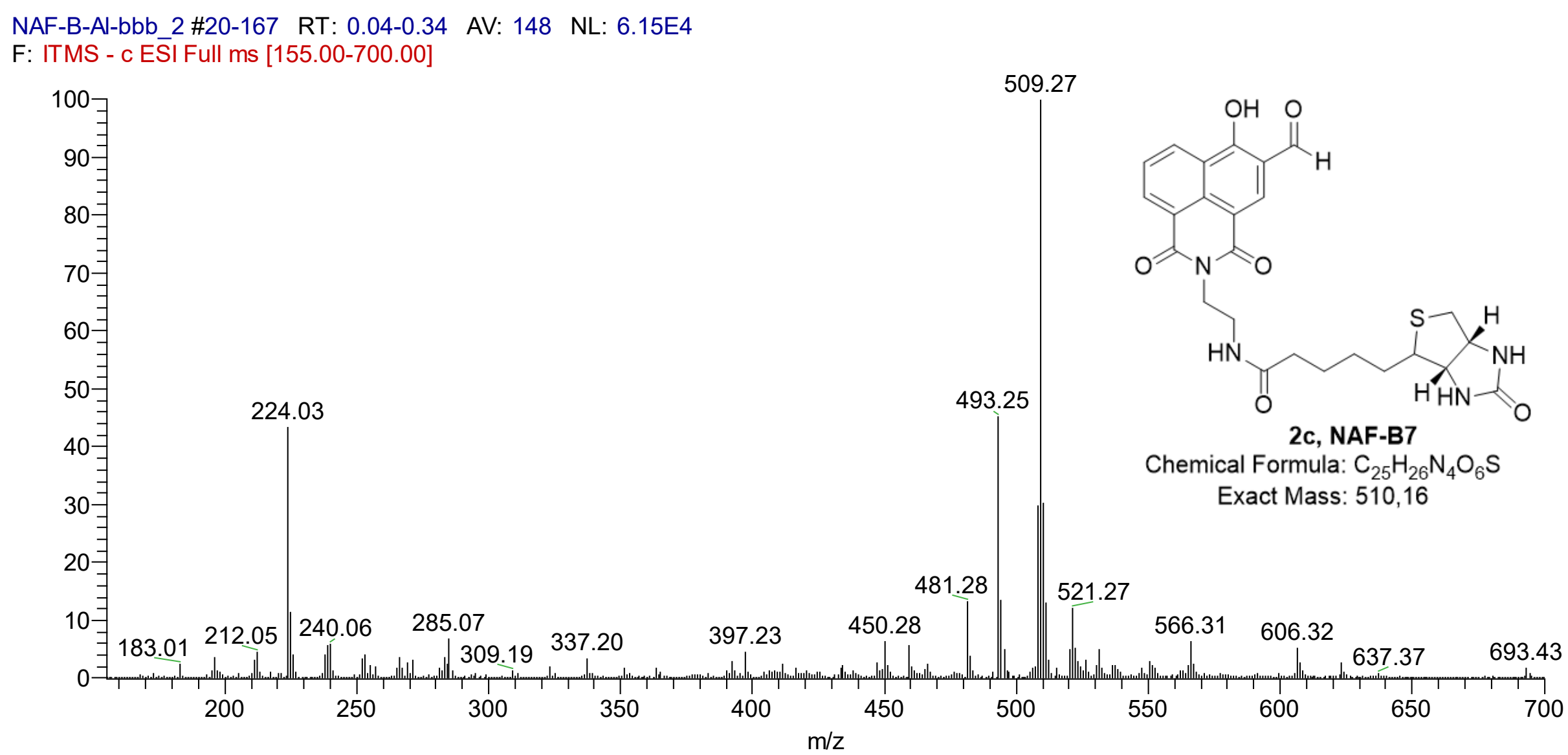


**Figure S35. UHPLC MS/MS in negative mode of compound 2c, NAF-B7.**

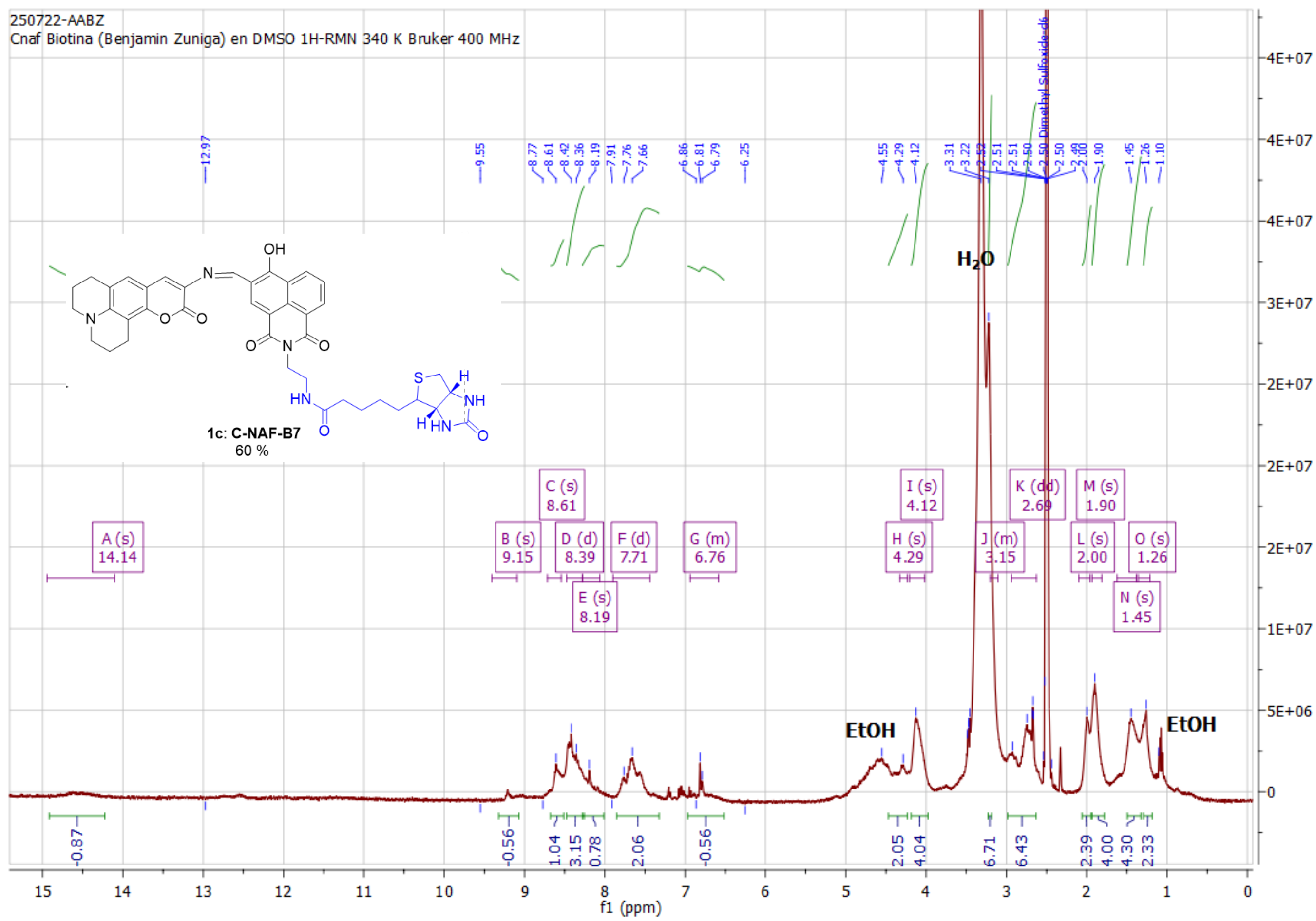


**Figure S36.** **$^{1}$H-NMR in DMSO-d$_6$ of compound 1c (C-NAF-B7).**

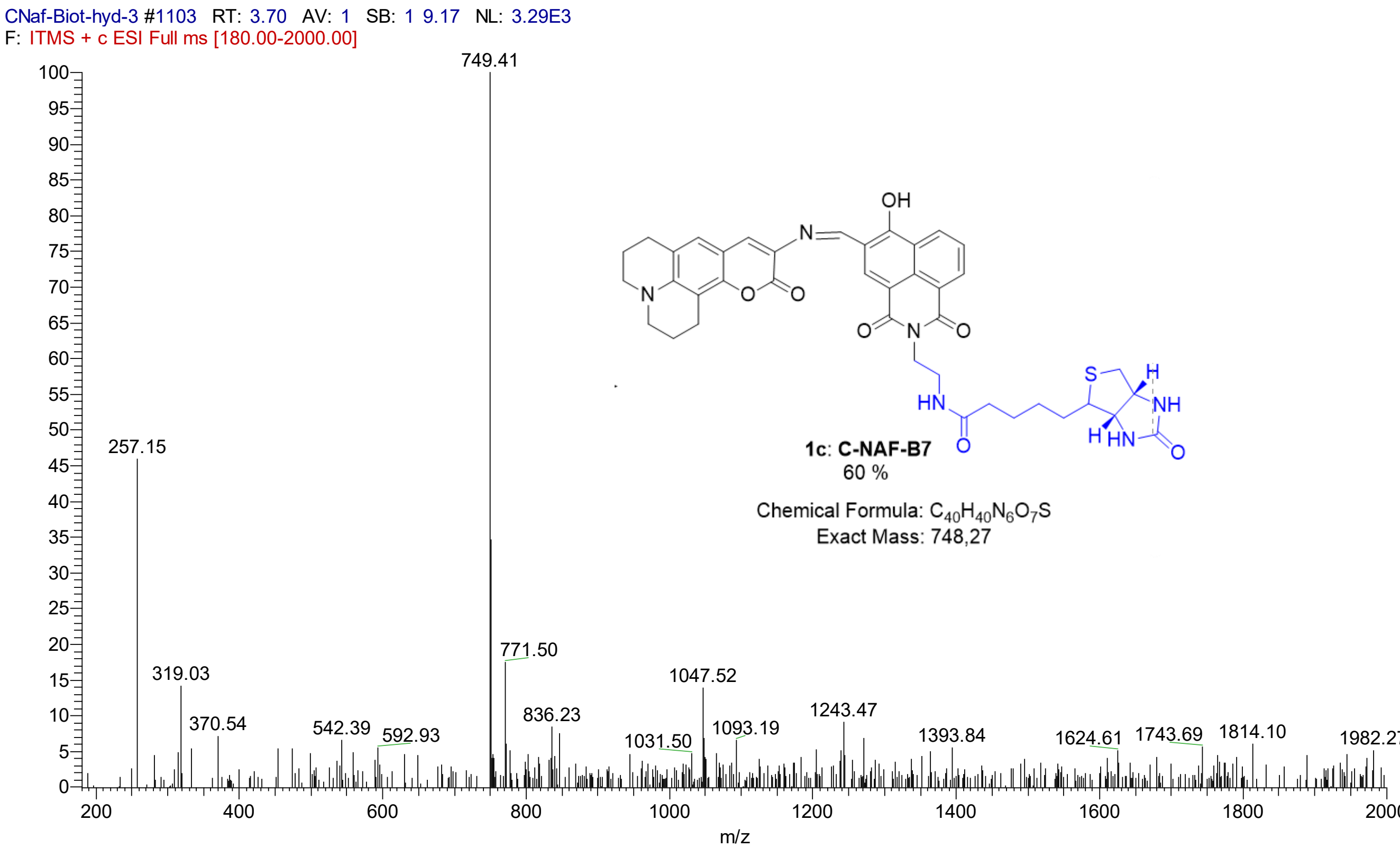


**Figure S38. UHPLC MS/MS in positive mode of compound 1c (C-NAF-B7).**

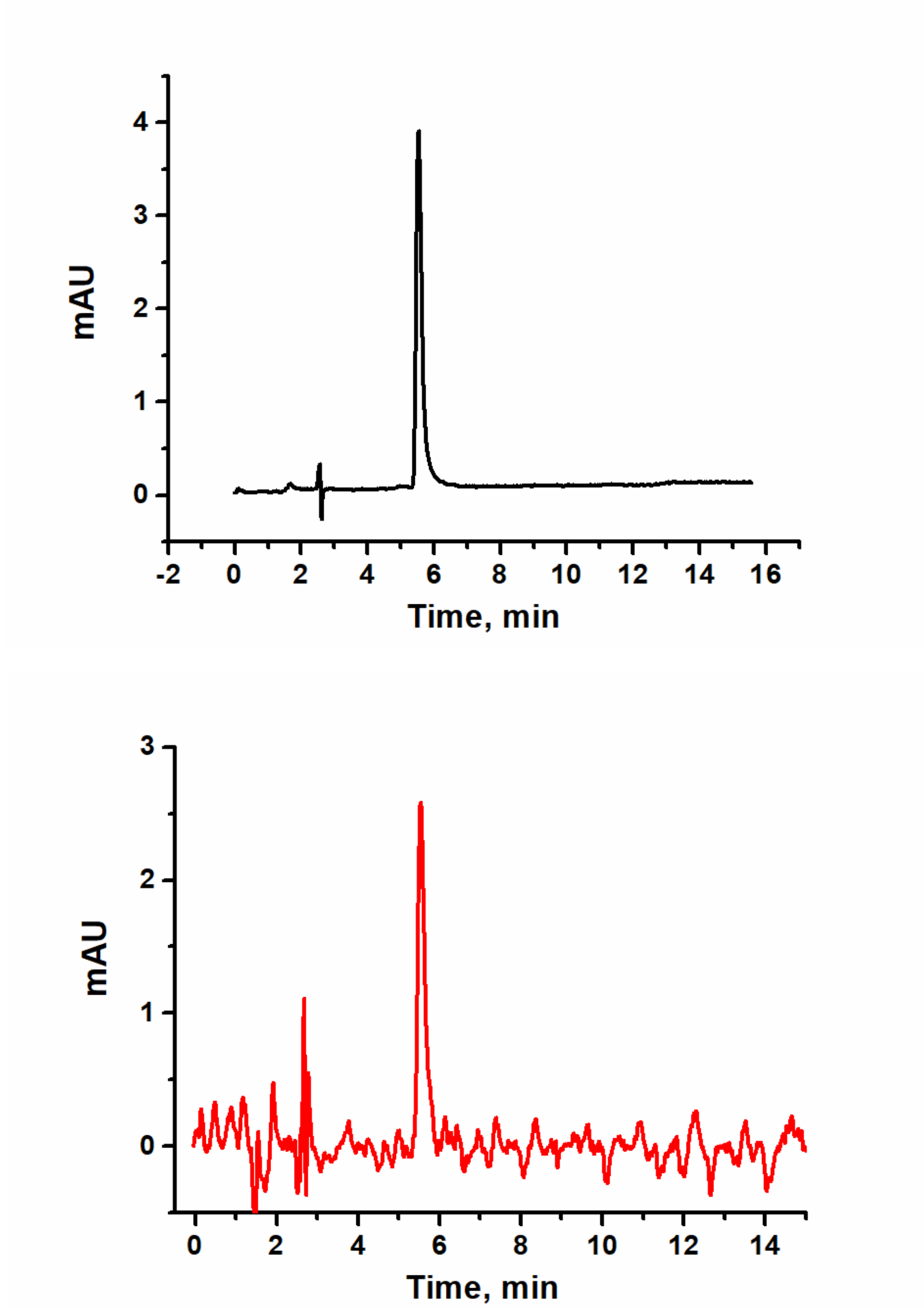


**Figure S39. Chromatogram of C-NAF-B7 obtained by HPLC-DAD. The sample was thermostated at 25 °C and analyzed under isocratic conditions using 100% acetonitrile as the mobile phase at a flow rate of 1.0 mL min$^{-1}$. Top: detection at 550 nm; bottom: detection at 220 nm.**

## S3. Additional electronic and photophysical properties

### S3.1 Molecular orbital frontiers of C-NAF and C-NAF-M

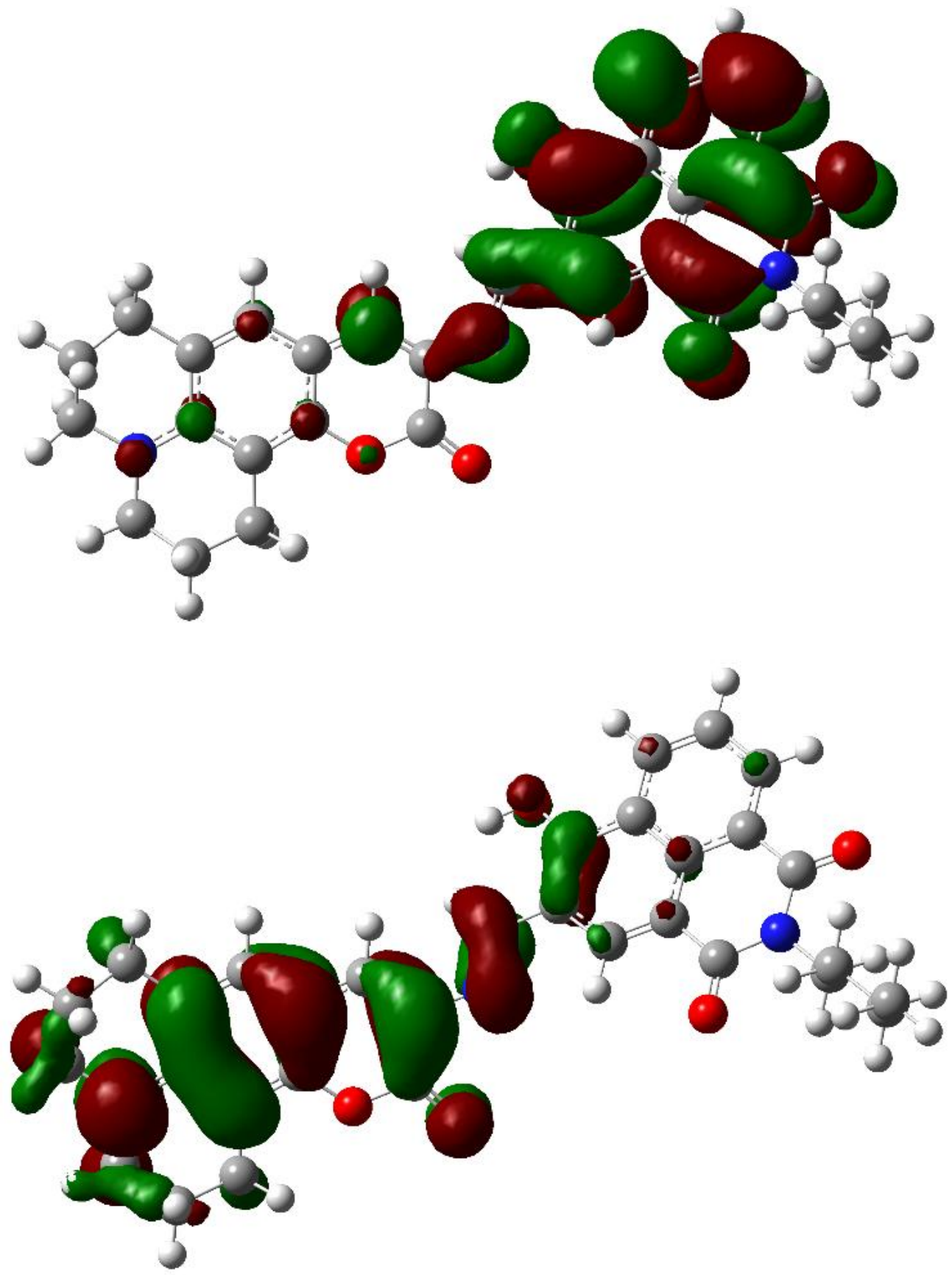

A

B

**Figure S40. Molecular orbital surfaces of the LUMO (A) and HOMO (B) of C-NAF, calculated at the B3LYP/6-31G(d,p) level of theory in methanol. Solvent effects were modeled using the C-PCM approach.**

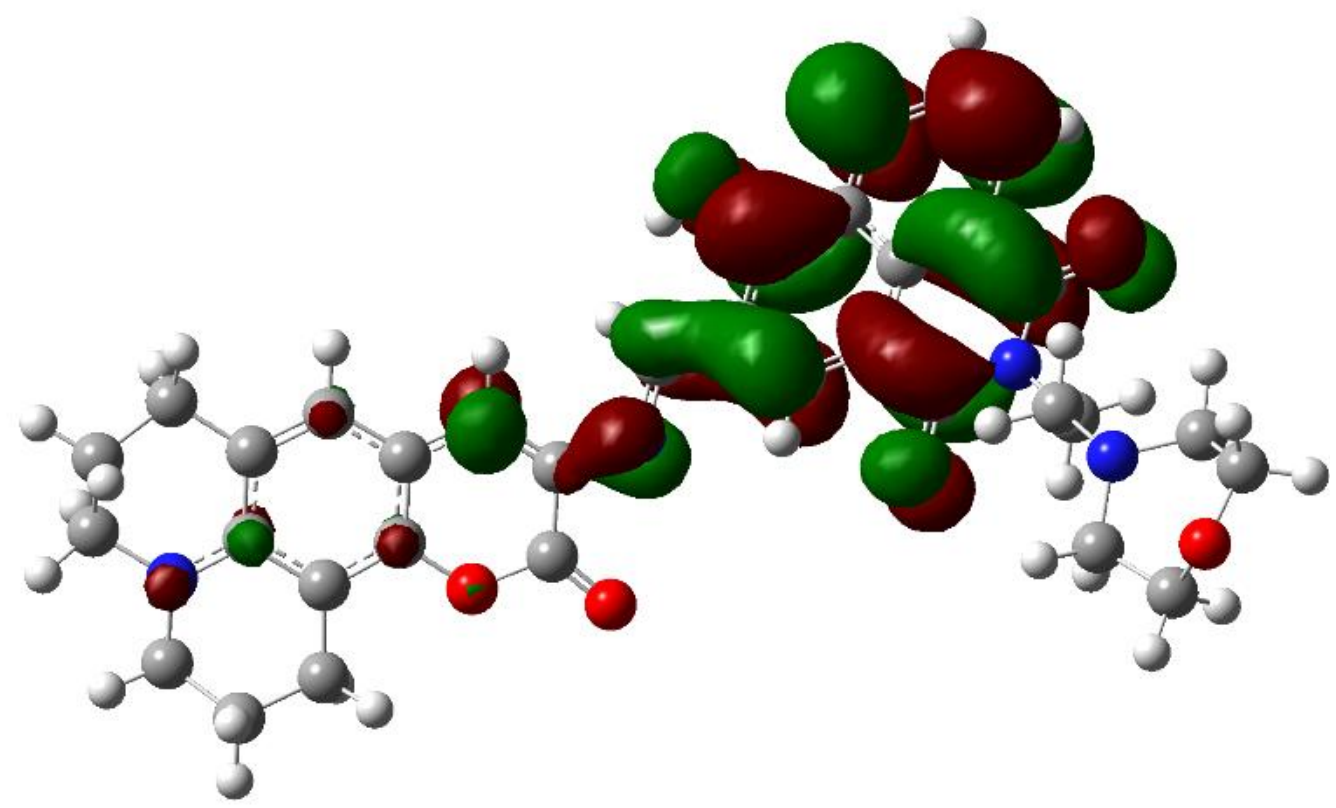

A

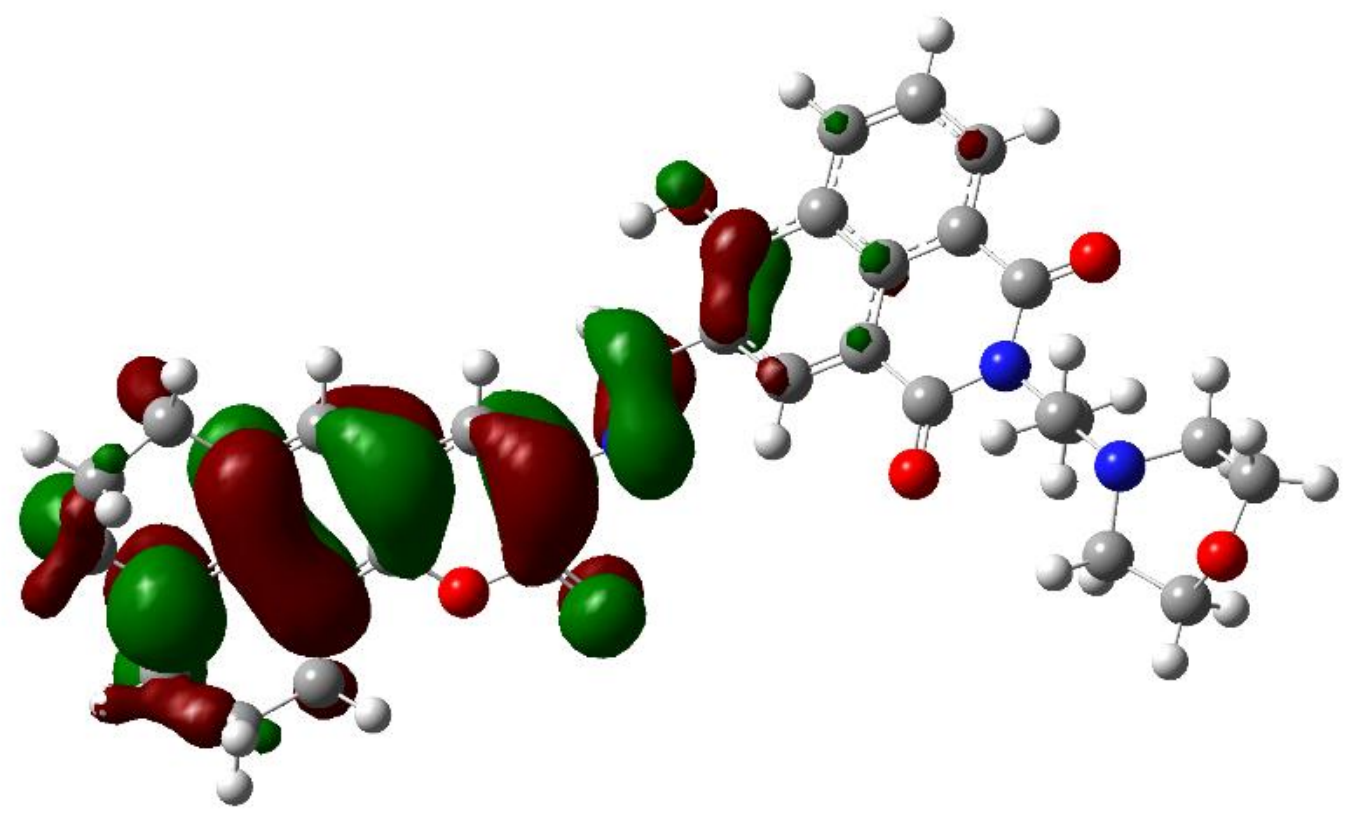

B

**Figure S41. Molecular orbital surfaces of the LUMO (A) and HOMO (B) of C-NAF-M, calculated at the B3LYP/6-31G(d,p) level of theory in methanol. Solvent effects were modeled using the C-PCM approach.**

**S3.2 Absorption spectra of NAF derivatives and Coumarin amine**

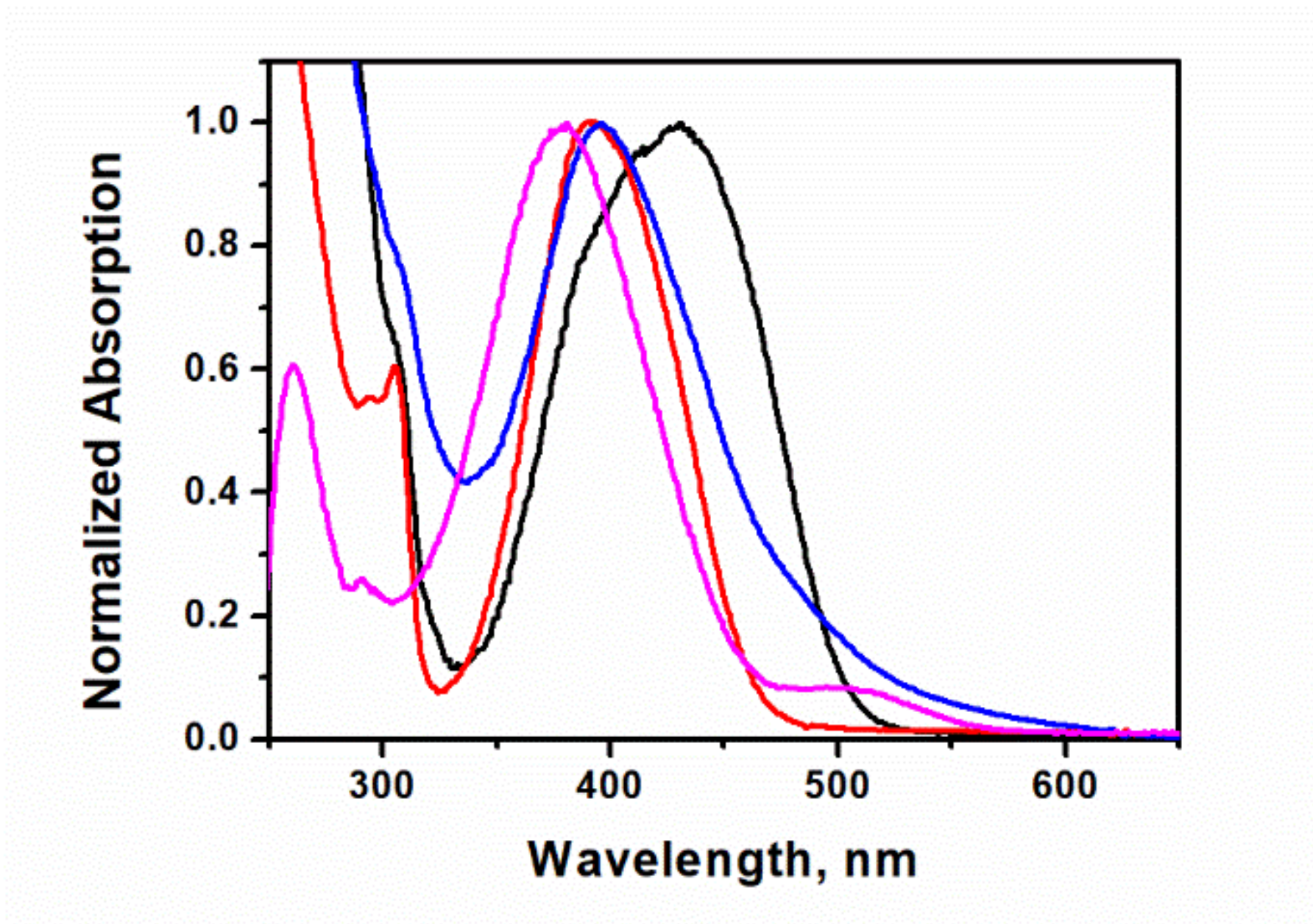


**Figure S42. Normalized absorption spectra of NAF derivatives and coumarin amine in methanol. NAF (-), C-NAF-M (-), C-NAF-B7 (-) and C-$NH_2$ (-).**

# S4. Identification of hydrolysis products by UHPLC-MS/MS and kinetics studies

## S4.1 Mass Spectrum of hydrolysis of C-NAF

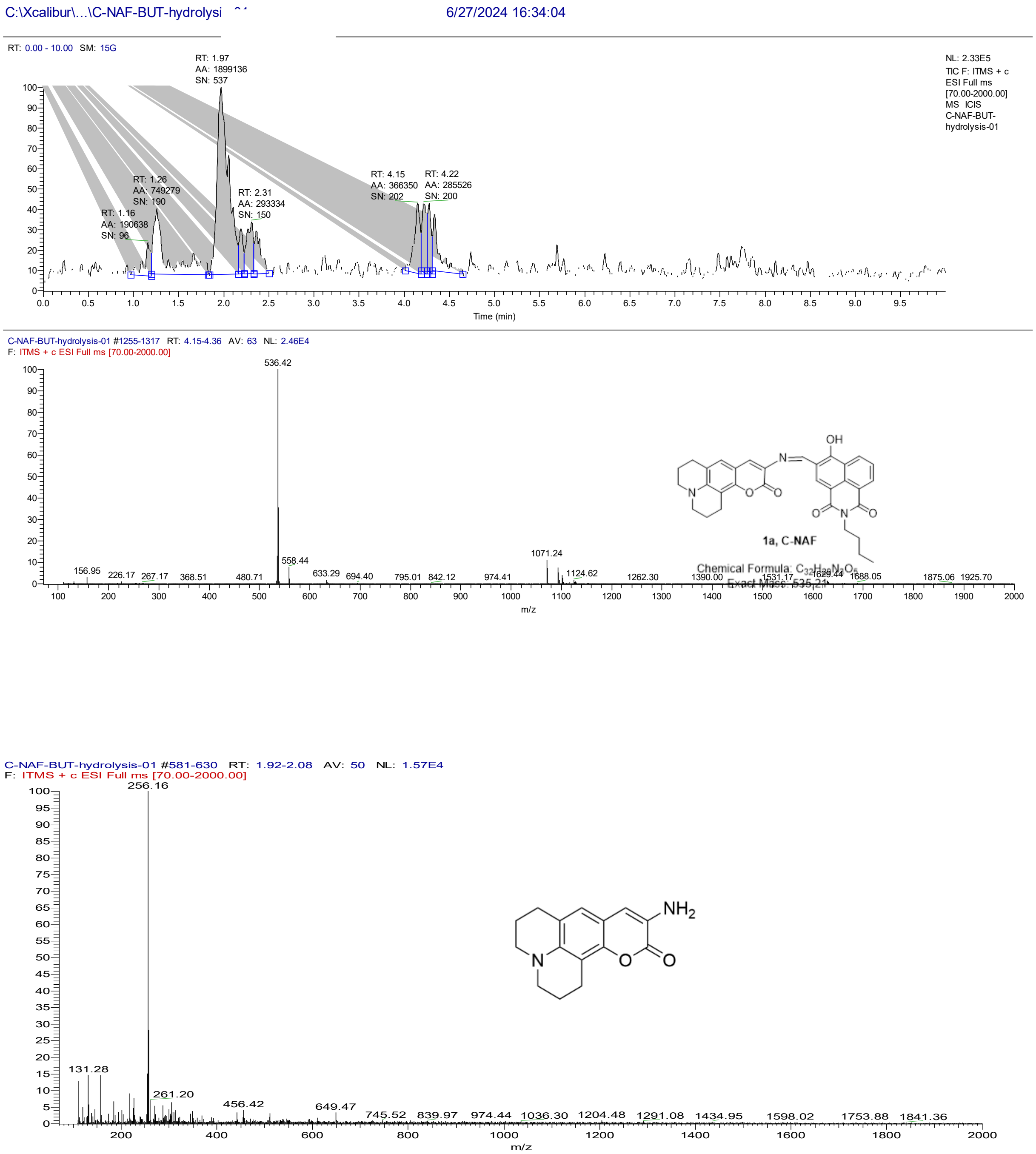


**Figure S43. Chromatogram and mass spectrum of C-NAF hydrolysis in methanol determined by UHPLC MS/MS, positive mode.**

**S4.2 Mass Spectrum of hydrolysis of C-NAF-M**

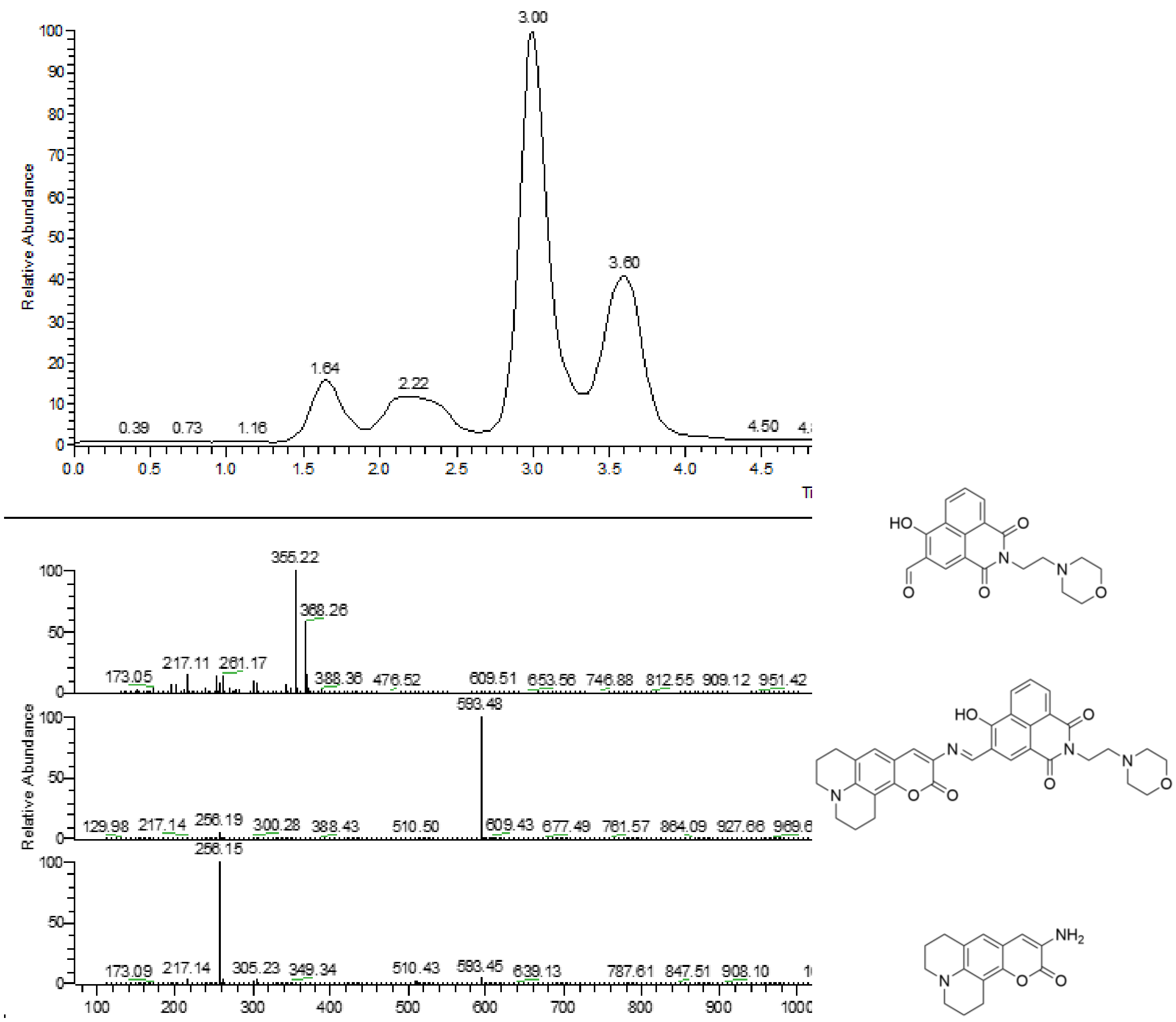


**Figure S44. Chromatogram and mass spectrum of C-NAF-M hydrolysis in methanol determined by UHPLC MS/MS, positive mode.**

### S4.3 Mass Spectrum of hydrolysis of C-NAF-B7

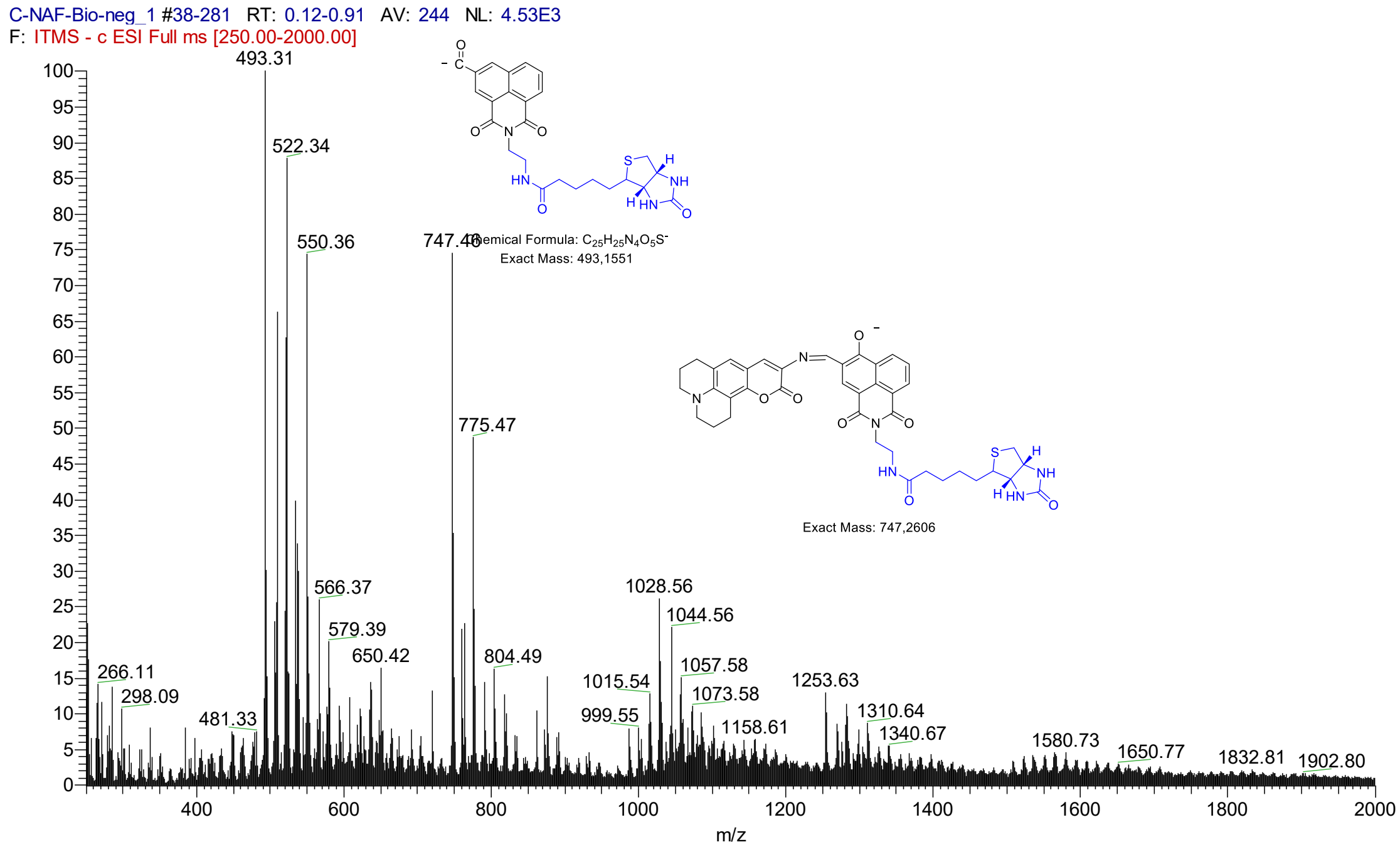


**Figure S45. Mass spectrum of C-NAF-B7 hydrolysis in methanol determined by direct injection in UHPLC MS/MS, positive mode.**

**S4.4. Hydrolysis studies and kinetic analysis**

**Table S1. Observed pseudo-first-order rate constants ($k_{obs}$), hydrolysis half-lives ($t_{1/2}$), and coefficients of determination obtained from the nonlinear fitting of the time-dependent UV–Vis absorption data for the C-NAF derivatives.**

| Compound | $K_{obs}$ ($min^{-1}$) | $t_{1/2}$ (min) | $R^2$ |
|---|---|---|---|
| C-NAF | 0.078 | 8.89 | 0.9998 |
| C-NAF-M | 0.054 | 12.74 | 0.9986 |
| C-NAF-B7 | 0.047 | 14.52 | 0.9995 |

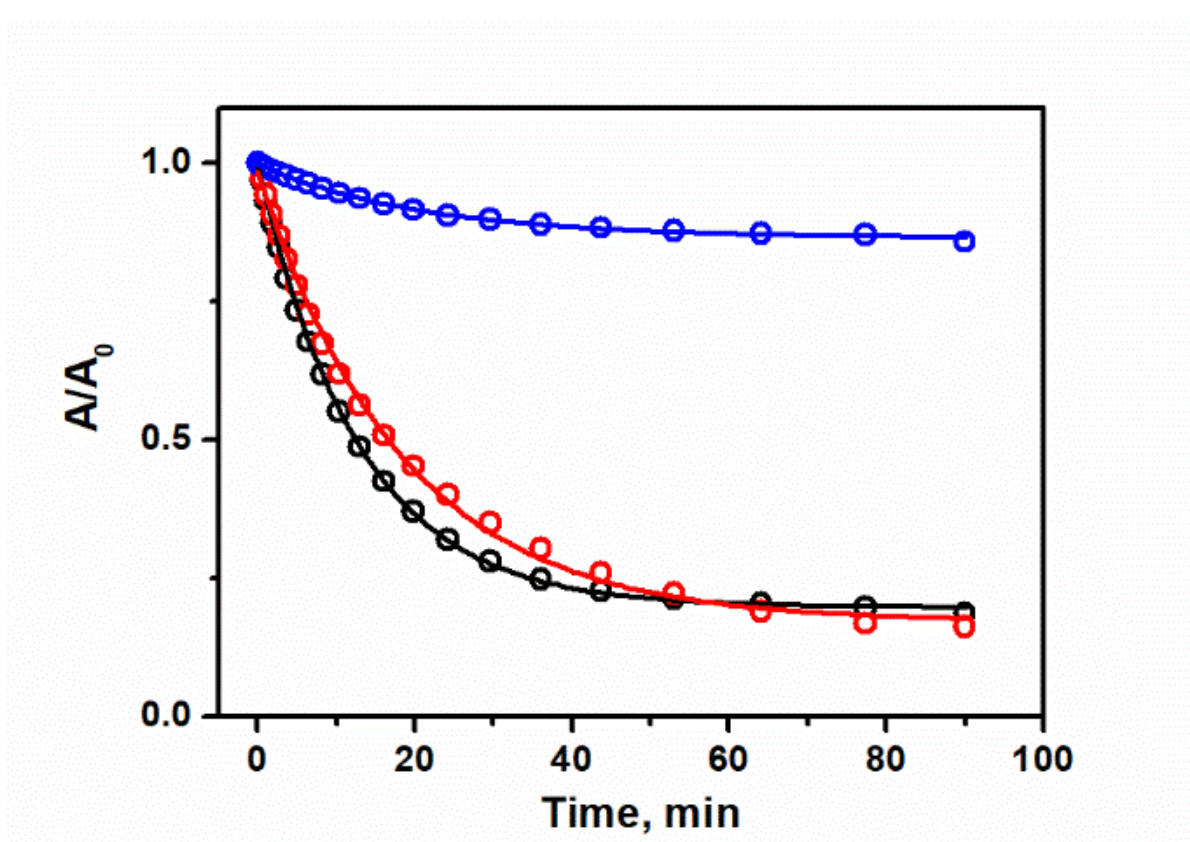


**Figure S46. Normalized time-dependent hydrolysis profiles of C-NAF, C-NAF-M, and C-NAF-B7 in methanol containing 0.02% water under aerobic conditions, monitored by UV–Vis absorption spectroscopy at 530–540 nm. Absorbance values were normalized to the initial absorbance of each derivative ($A_t/A_0$). Symbols represent experimental data, while solid lines correspond to nonlinear fits using a pseudo-first-order monoexponential decay model.**

## S5. Singlet oxygen generation and ROS-related experiments

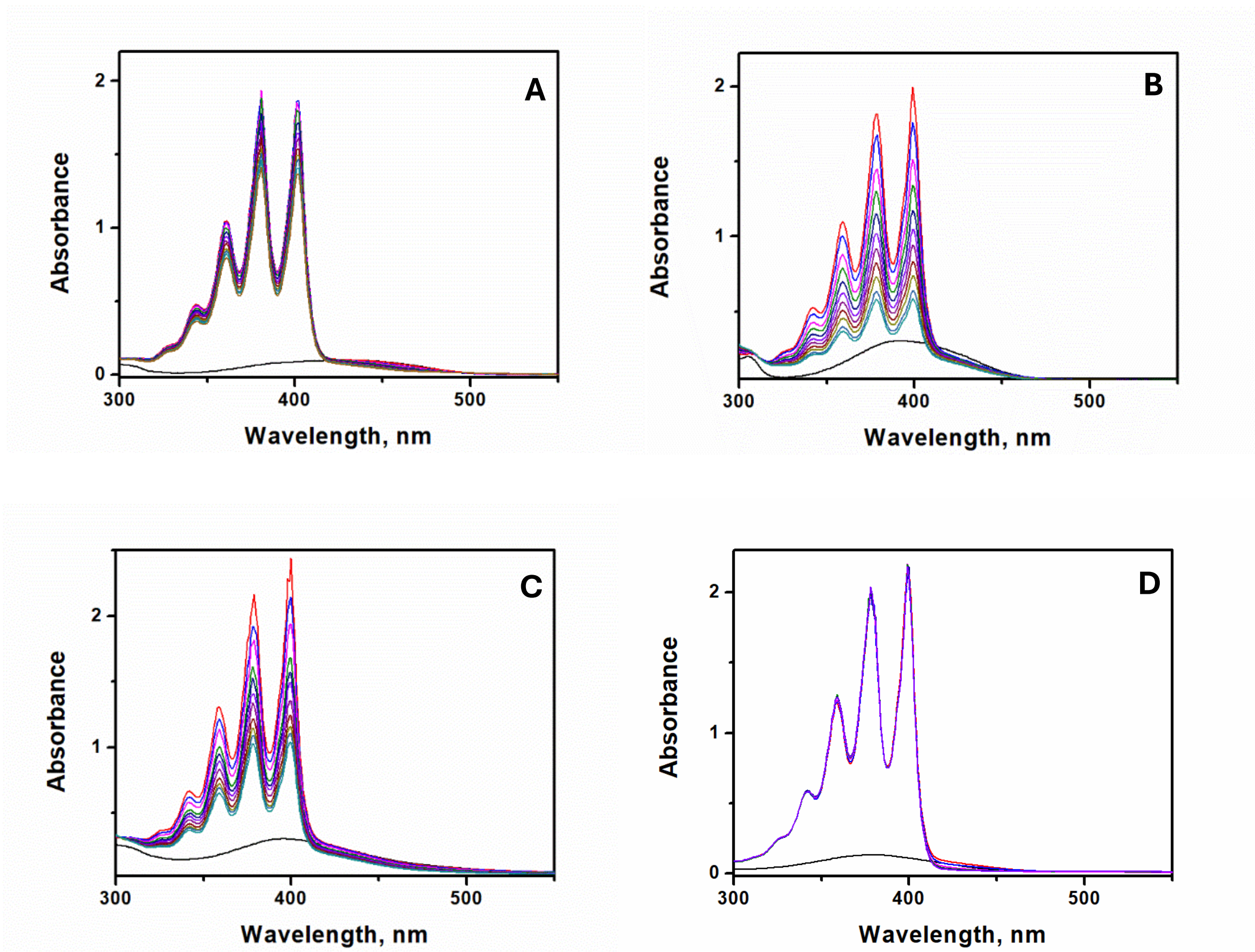


**Figure S47. Consumption of ABMA (200 mM) by irradiation LED of NAF derivatives (2.5 μM) at 460 nm in ultrapure water. NAF (A), NAF-M (B), NAF-B7 (C) and C-$NH_2$ (D)**

## S6. Stability under biologically relevant conditions

### S6.1 Fluorescence stability in DMEM conditions

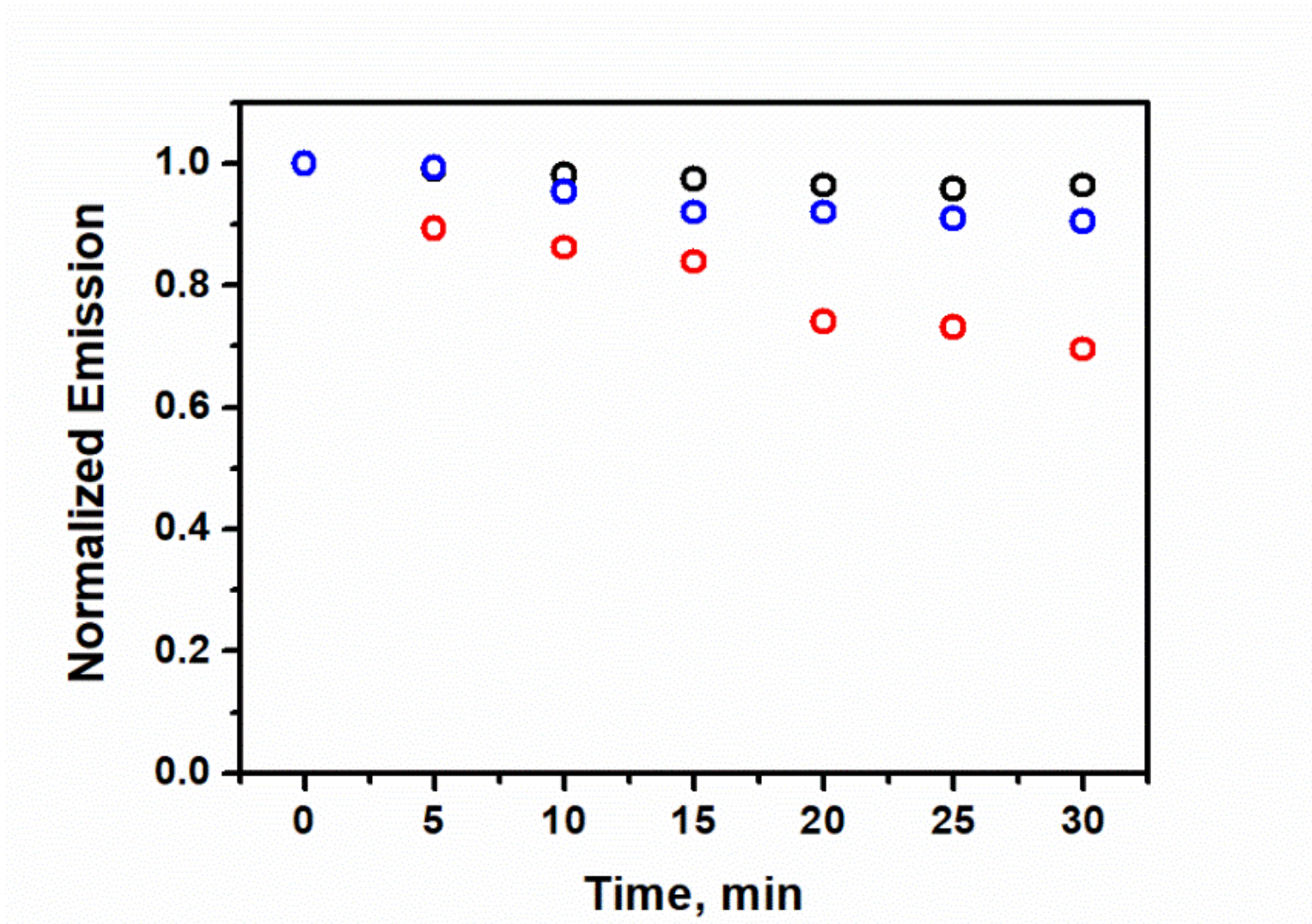


**Figure S48. Normalized fluorescence emission intensity ($I/I_0$) of C-NAF (black), C-NAF-M (red), and C-NAF-B7 (blue) in DMEM under continuous 540 nm irradiation for 30 min. Fluorescence intensity was normalized to the initial value ($I_0$). The gradual decrease in emission intensity indicates photoinduced hydrolytic degradation of the C-NAF derivatives under prolonged irradiation.**

**S6.2 Evaluation of the spectral stability of the C-NAF derivatives at different pH values**

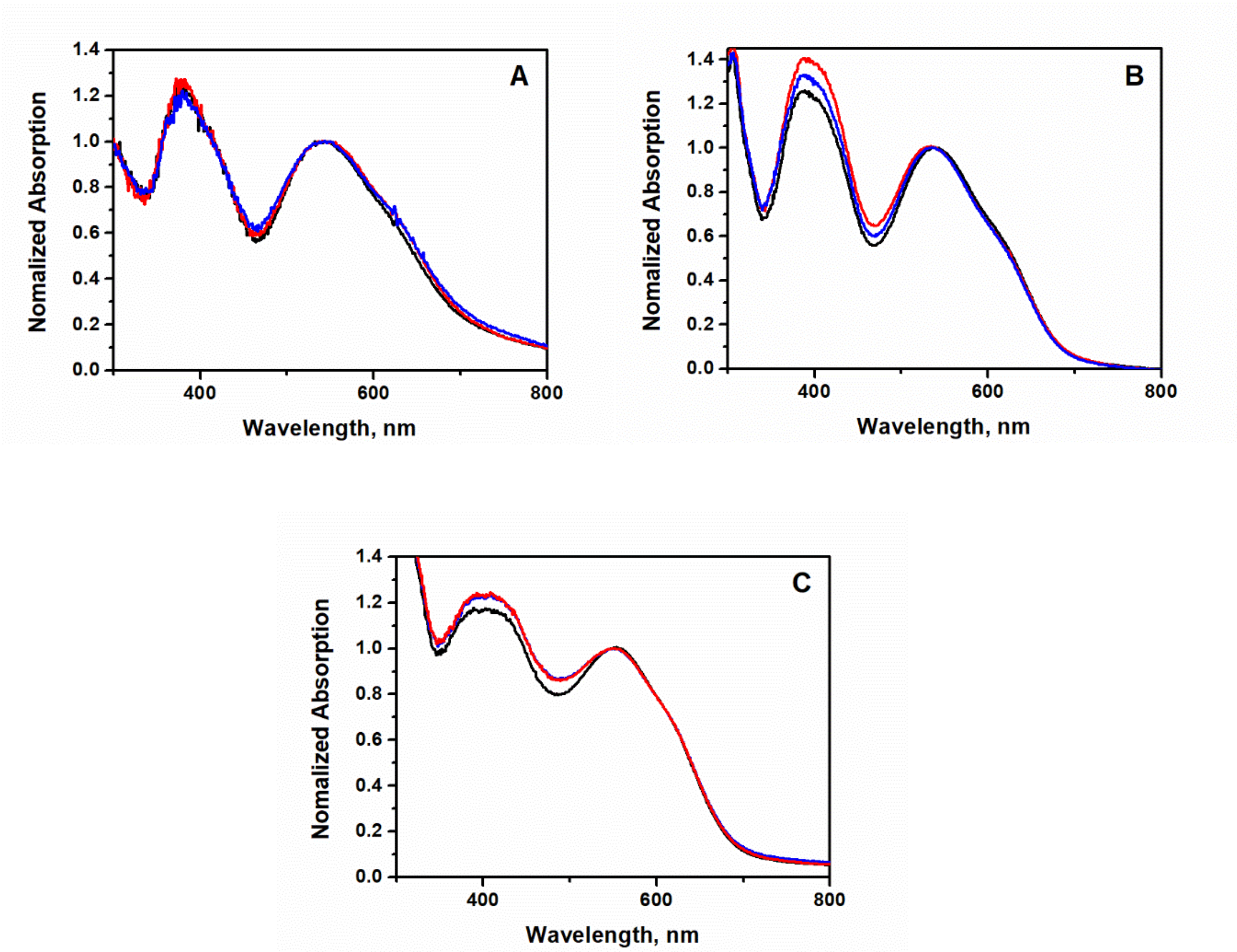


**Figure S49. Effect of pH on the absorption spectra of the C-NAF derivatives. Normalized UV–Vis absorption spectra of (A) C-NAF, (B) C-NAF-M, and (C) C-NAF-B7 recorded at pH 5.0 (black), pH 7.4 (red), and pH 8.0 (blue).**

## S7. In vitro cytotoxicity and phototoxicity controls of C-NAF derivatives in MCF-7

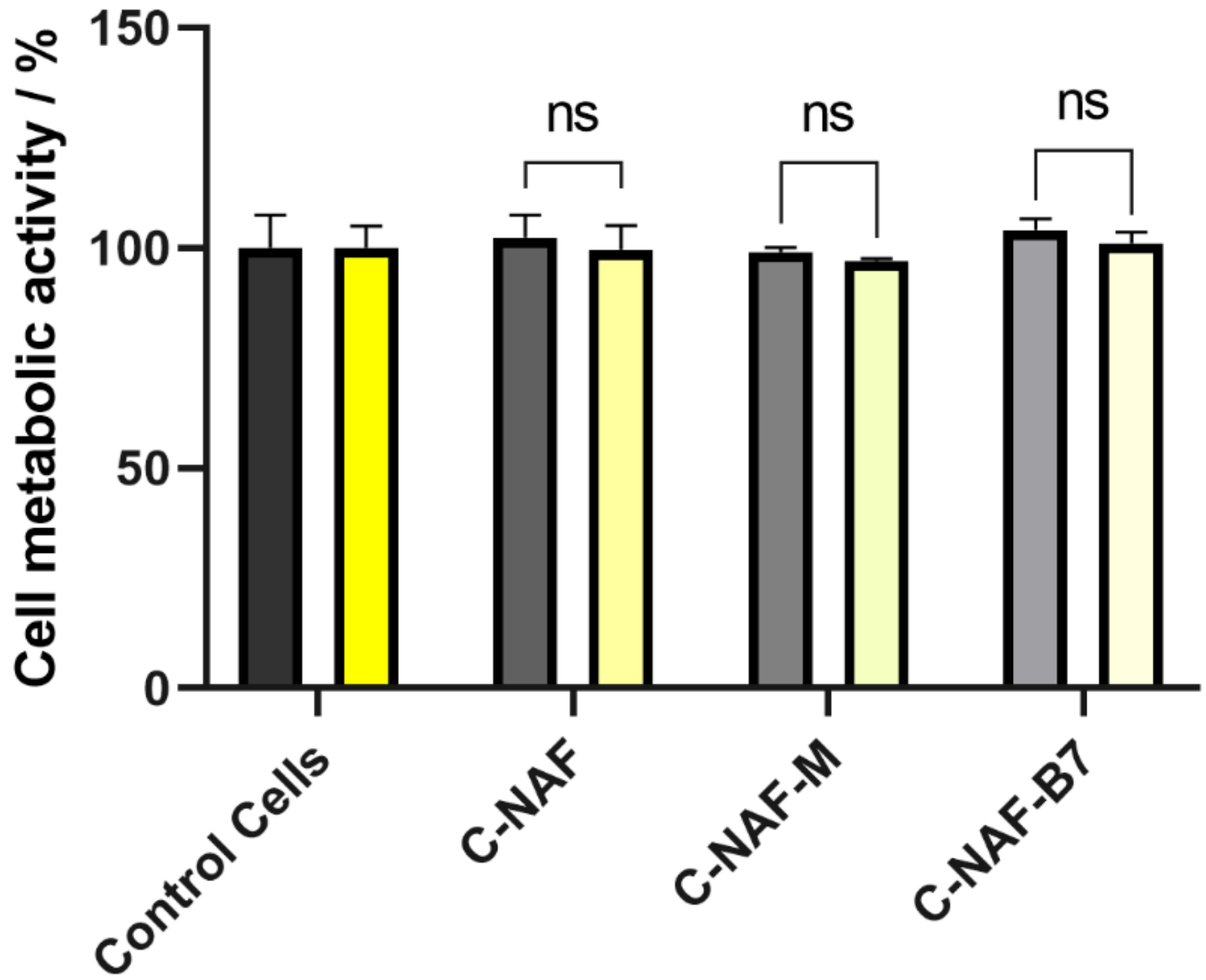


**Figure S50. Dark toxicity (grey) and phototoxicity (yellow) of C-NAF derivatives (1.0 μM) on MCF-7 cells. Incubation for 90 min followed by irradiation with 540 nm LEDs for 60 min. NS: no significance.**

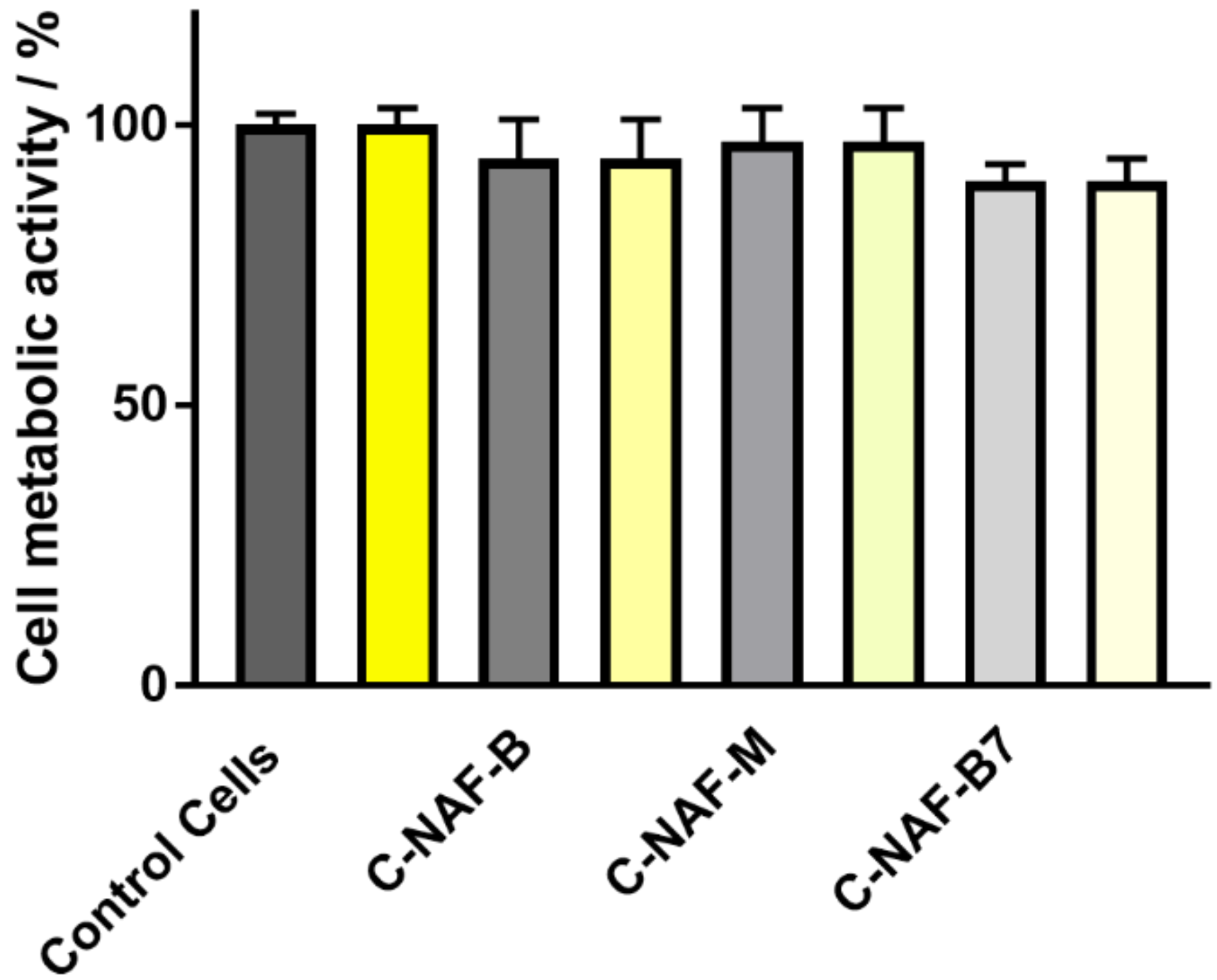


**Figure S51. Dark toxicity (grey) and phototoxicity (yellow) of C-NAF derivatives (1.0 μM) on MCF-7 cells. Incubation for 15 min followed by irradiation with 460 nm LEDs for 60 min. Statistical significance of *p* < 0.0001 (****).**
